# Phenological Flexibility in Nesting Grassland Birds Over a Half Century Informs Timing of Management

**DOI:** 10.1111/gcb.71128

**Published:** 2026-10-12

**Authors:** Kristen S. Ellis, Michael J. Anteau, Lawrence D. Igl, Garrett J. MacDonald, Neal D. Niemuth, Aaron T. Pearse, Jill A. Shaffer, Christine A. Ribic, Josh L. Vest, Masanori Abe, Daniel S. Ackerman, Marissa A. Ahlering, Johanna N. Alpert, Anahit Amiri, Kent Andersson, Jacqueline K. Augustine, Kristel K. Bakker, Jeffrey L. Beck, Adam C. Behney, Jacy S. Bernath‐Plaisted, Todd R. Bogenschutz, Eric K. Bollinger, Lauren E. Bortolotti, Andy J. Boyce, Jeffrey D. Brawn, Fred C. Bryant, Andrew J. Campomizzi, Jay D. Carlisle, J. Matthew Carroll, Rachel Carroll, John P. Carroll, Justin R. Clarke, Michael R. Conover, Reesa Y. Conrey, Jaime J. Coon, Kyle A. Cutting, Stephen K. Davis, Dawn M. Davis, Duane R. Diefenbach, Jonathan B. Dinkins, R. Andrew Dreelin, Victoria J. Dreitz, Courtney J. Duchardt, Michael W. Eichholz, Susan N. Ellis‐Felege, Kevin S. Ellison, Bradley C. Fedy, Samantha R. Fino, Robert J. Fletcher, Dillon T. Fogarty, Samuel D. Fuhlendorf, Pamela R. Garrettson, Robert J. Gates, Erin L. Gelling, James J. Giocomo, Alex Glass, Cheri L. Gratto‐Trevor, Kirsty E. B. Gurney, Ryan D. Haffele, Christian A. Hagen, Vanessa B. Harriman, Samuel W. H. Harryman, David A. Haukos, Robert T. Haynam, Heather A. Herakovich, James R. Herkert, Fidel Hernández, Jason M. Hill, H. Jeffrey Homan, Torre J. Hovick, Jonathan A. Jenks, William E. Jensen, Aaron N. Johnston, Sydney‐Marie Jones, Carly S. Kelly, Kaylan M. Kemink, Silka L. F. Kempema, Dylan C. Kesler, Christopher P. Kirol, Robert W. Klaver, Page E. Klug, Jesse L. Kolar, Nicola Koper, Kyle J. Kuechle, Jonathan D. Lautenbach, Zoe M. Lebrun‐Southcott, David W. Londe, Scott R. Loss, Cullen M. Mackenzie, Patrick A. Magee, Douglas L. Manzer, John P. McCarty, Gwyn McKee, Ann E. McKellar, Lance B. McNew, James R. Miller, Megan C. Milligan, Ashlee K. Minor, Rocco J. Murano, Les D. Murray, David E. Naugle, Clayton K. Nielsen, Christopher J. Norment, Sarah B. Ogden, Lindsey A. Parsons, Brian D. Peer, Noah G. Perlut, James C. Pitman, Christopher D. Pollentier, Larkin A. Powell, Aaron C. Pratt, John P. Pulliam, Nancy M. Raginski, Dan L. Reinking, Kaitlyn M. Reintsma, Terry Z. Riley, Kevin M. Ringelman, Joseph R. Robb, Scott K. Robinson, Andres N. Rosales, Charlotte L. Roy, Kayla A. Ruth, Brett K. Sandercock, Kevin K. Sedivec, Steve K. Sherrod, Justin J. Shew, Mason L. Sieges, Jenny L. Sika, Susan K. Skagen, Cassandra G. Skaggs, Joseph Smith, Jennifer A. Smith, Kurt T. Smith, Alex J. Solem, Joshua D. Stafford, Glenn E. Stauffer, Matthew D. Stephenson, Lee Sutcliffe, Amber E. Swicegood, Jason Tack, Evan P. Tanner, Catrina V. Terry, Wendy P. Tori, Ashley M. Unger, Kelly R. VanBeek, William E. Vetter, Jay A. VonBank, Brett L. Walker, Michael D. Whitson, Maiken Winter, Kimberly A. With, Donald H. Wolfe, Amy A. Yackel Adams, Benjamin Zuckerberg

**Affiliations:** ^1^ Northern Prairie Wildlife Research Center U.S. Geological Survey Jamestown North Dakota USA; ^2^ Retired, Northern Prairie Wildlife Research Center U.S. Geological Survey Jamestown North Dakota USA; ^3^ Retired, Habitat and Population Evaluation Team U.S. Fish and Wildlife Service Bismarck North Dakota USA; ^4^ Retired, Department of Forest and Wildlife Ecology University of Wisconsin‐Madison Madison Wisconsin USA; ^5^ Prairie Pothole Joint Venture U.S. Fish and Wildlife Service Great Falls Montana USA; ^6^ Western Riverside County, MSHCP Biological Monitoring Program Riverside California USA; ^7^ Meadowlark Environmental LLC Mandan North Dakota USA; ^8^ The Nature Conservancy Minneapolis Minnesota USA; ^9^ Natural Resources Institute University of Manitoba Winnipeg Manitoba Canada; ^10^ School of Biological Sciences Southern Illinois University Carbondale Carbondale Illinois USA; ^11^ Department of Natural Resource Ecology and Management Oklahoma State University Stillwater Oklahoma USA; ^12^ Audubon of Kansas Manhattan Kansas USA; ^13^ College of Arts and Sciences Dakota State University Madison South Dakota USA; ^14^ Department of Ecosystem Science and Management University of Wyoming Laramie Wyoming USA; ^15^ Colorado Parks and Wildlife Fort Collins Colorado USA; ^16^ Department of Forest and Wildlife Ecology University of Wisconsin‐Madison Madison Wisconsin USA; ^17^ Bird Conservancy of the Rockies Fort Collins Colorado USA; ^18^ Iowa Department of Natural Resources Boone Iowa USA; ^19^ Department of Biological Sciences Eastern Illinois University Charleston Illinois USA; ^20^ Institute for Wetland and Waterfowl Research Ducks Unlimited Canada Stonewall Manitoba Canada; ^21^ Smithsonian's National Zoo and Conservation Biology Institute Washington, DC USA; ^22^ Department of Natural Resources and Environmental Sciences University of Illinois Urbana‐Champaign Urbana Illinois USA; ^23^ Caesar Kleberg Wildlife Research Institute Texas A&M University‐Kingsville Kingsville Texas USA; ^24^ Bird Ecology and Conservation Ontario Toronto Ontario Canada; ^25^ Intermountain Bird Observatory Boise State University Boise Idaho USA; ^26^ Department of Biological Sciences Murray State University Murray Kentucky USA; ^27^ Jones College of Science, Engineering and Technology Murray State University Murray Kentucky USA; ^28^ School of Natural Resources University of Nebraska‐Lincoln Lincoln Nebraska USA; ^29^ School of Natural Resource Sciences North Dakota State University Fargo North Dakota USA; ^30^ Central Grasslands Research Extension Center North Dakota State University Streeter North Dakota USA; ^31^ Wildland Resources Department Utah State University Logan Utah USA; ^32^ Department of Biology Earlham College Richmond Indiana USA; ^33^ Department of Environmental Sustainability Earlham College Richmond Indiana USA; ^34^ Red Rock Lakes National Wildlife Refuge U.S. Fish and Wildlife Service Lima Montana USA; ^35^ Canadian Wildlife Service Regina Saskatchewan Canada; ^36^ U.S. Fish and Wildlife Service Bend Oregon USA; ^37^ Pennsylvania Cooperative Fish and Wildlife Research Unit Pennsylvania State University University Park Pennsylvania USA; ^38^ Department of Animal and Rangeland Sciences Oregon State University Corvallis Oregon USA; ^39^ Department of Biological Sciences Northern Illinois University Dekalb Illinois USA; ^40^ Avian Science Center University of Montana Missoula Montana USA; ^41^ School of Natural Resources and the Environment University of Arizona Camp Verde Arizona USA; ^42^ Department of Biology University of North Dakota Grand Forks North Dakota USA; ^43^ American Bird Conservancy Bozeman Montana USA; ^44^ School of Environment, Resources and Sustainability University of Waterloo Waterloo Ontario Canada; ^45^ South Dakota State University Brookings South Dakota USA; ^46^ Department of Zoology, Conservation Research Institute University of Cambridge Cambridge UK; ^47^ U.S. Fish and Wildlife Service Laurel Maryland USA; ^48^ School of Environment and Natural Resources The Ohio State University Columbus Ohio USA; ^49^ American Bird Conservancy Durand Illinois USA; ^50^ Lees‐McRae College Banner Elk North Carolina USA; ^51^ Science and Technology Branch Environment and Climate Change Canada Saskatoon Saskatchewan Canada; ^52^ Wisconsin Department of Natural Resources Wisconsin Rapids Wisconsin USA; ^53^ Department of Fisheries, Wildlife, and Conservation Sciences Oregon State University Corvallis Oregon USA; ^54^ Texas Parks & Wildlife Department Lubbock Texas USA; ^55^ Kansas Cooperative Fish and Wildlife Research Unit U.S. Geological Survey Manhattan Kansas USA; ^56^ Northern Rocky Mountain Science Center U.S. Geological Survey Bozeman Montana USA; ^57^ National Great Rivers Research and Education Center East Alton Illinois USA; ^58^ Retired Illinois Endangered Species Protection Board Springfield Illinois USA; ^59^ Vermont Center for Ecostudies White River Junction Vermont USA; ^60^ Retired, National Wildlife Research Center U.S. Department of Agriculture Bismarck North Dakota USA; ^61^ Emporia State University Emporia Kansas USA; ^62^ University of Saskatchewan Saskatoon Saskatchewan Canada; ^63^ Western EcoSystems Technology Inc. Laramie Wyoming USA; ^64^ Ducks Unlimited Inc. Bismarck North Dakota USA; ^65^ South Dakota Department of Game, Fish, and Parks Pierre South Dakota USA; ^66^ The Institute for Bird Populations San Diego California USA; ^67^ Kirol Applied Ecological Research Sheridan Wyoming USA; ^68^ Iowa Cooperative Fish and Wildlife Research Unit U.S. Geological Survey Ames Iowa USA; ^69^ National Wildlife Research Center USDA‐APHIS‐Wildlife Services Fargo North Dakota USA; ^70^ North Dakota Game and Fish Department Bismarck North Dakota USA; ^71^ Faculty of Environment University of Northern British Columbia Prince George British Columbia Canada; ^72^ U.S. Fish and Wildlife Service Indiahoma Oklahoma USA; ^73^ Department of Natural and Environmental Sciences Western Colorado University Gunnison Colorado USA; ^74^ Alberta Conservation Association Blairmore Alberta Canada; ^75^ Department of Biology University of Nebraska at Omaha Omaha Nebraska USA; ^76^ Great Plains Wildlife Consulting Inc. Banner Wyoming USA; ^77^ Environment and Climate Change Canada Saskatoon Saskatchewan Canada; ^78^ Department of Animal and Range Sciences Montana State University Bozeman Montana USA; ^79^ U.S. Geological Survey, Alaska Cooperative Fish and Wildlife Research Unit University of Alaska Fairbanks Fairbanks Alaska USA; ^80^ Division of Science and Engineering Penn State University—Abington Abington Pennsylvania USA; ^81^ Franke College of Forestry and Conservation University of Montana Missoula Montana USA; ^82^ Center for Wildlife Sustainability Research Southern Illinois University Carbondale Carbondale Illinois USA; ^83^ Department of Environmental Science and Ecology SUNY Brockport Brockport New York USA; ^84^ Bowman Consulting Group Ltd. Missoula Montana USA; ^85^ Department of Biology Stephen F. Austin State University Nacogdoches Texas USA; ^86^ School of Marine and Environmental Programs University of New England Biddeford Maine USA; ^87^ Ducks Unlimited Inc. Emporia Kansas USA; ^88^ George Miksch Sutton Avian Research Center Bartlesville Oklahoma USA; ^89^ Warnell School of Forestry and Natural Resources University of Georgia Athens Georgia USA; ^90^ North American Grouse Partnership Fort Collins Colorado USA; ^91^ Department of Wildlife, Fish, and Conservation Biology University of California Davis Davis California USA; ^92^ Big Oaks and Muscatatuck National Wildlife Refuge Complex, U.S. Fish and Wildlife Service Madison Indiana USA; ^93^ Florida Museum of Natural History and the University of Florida Gainesville Florida USA; ^94^ Minnesota Department of Natural Resources Grand Rapids Minnesota USA; ^95^ Norwegian Institute for Nature Research Trondheim Norway; ^96^ U.S. Fish and Wildlife Service Helena Montana USA; ^97^ Retired, Fort Collins Science Center U.S. Geological Survey Fort Collins Colorado USA; ^98^ Louisiana State University AgCenter Baton Rouge Louisiana USA; ^99^ Department of Zoology and Physiology University of Wyoming Laramie Wyoming USA; ^100^ South Dakota Cooperative Fish and Wildlife Research Unit U.S. Geological Survey Brookings South Dakota USA; ^101^ Iowa State University Ames Iowa USA; ^102^ Habitat and Population Evaluation Team U.S. Fish and Wildlife Service Missoula Montana USA; ^103^ U.S. Fish and Wildlife Service Madison Wisconsin USA; ^104^ Precision Wildlife Resources LLC Gillette Wyoming USA; ^105^ Delta Waterfowl Foundation Bismarck North Dakota USA; ^106^ Department of Natural Resources Texas Tech University Lubbock Texas USA; ^107^ WissenLeben Raisting Germany; ^108^ Department of Biology, Laboratory for Landscape and Conservation Ecology Kansas State University Manhattan Kansas USA; ^109^ Retired George Miksch Sutton Avian Research Center Bartlesville Oklahoma USA; ^110^ Fort Collins Science Center U.S. Geological Survey Fort Collins Colorado USA

**Keywords:** grassland birds, grassland management, nest initiation dates, nesting period, phenology, quantile regression, vegetation green‐up

## Abstract

The timing of biological events for wildlife species reflects and influences ecological and evolutionary processes and helps guide management actions to meet conservation objectives. Climate change may cause phenological shifts across trophic levels and ecosystems, which can have broad ecological impacts. Shifts may also result in mistiming between management practices based on historical conditions and current biological events, reducing the effectiveness of natural resource management and conservation efforts. We analyzed nesting phenology patterns over recent decades across North America for 44 species of grassland‐nesting birds, including waterfowl, upland gamebirds, shorebirds, and passerines, many of which have experienced precipitous population declines. Understanding nesting phenologies of these species is important to their conservation, as grassland management activities often conflict with reproductive periods leading to high nest and young mortality. We compiled a dataset of over 172,000 nest records spanning five decades and evaluated environmental factors influencing long‐term shifts and short‐term flexibility in nesting phenology. We found considerable variation in responses among species, with differences in both direction and magnitude of spatiotemporal effects on nest initiation dates. Notably, the community‐level nesting season lengthened over time, but shifts exhibited spatial heterogeneity within this community of grassland birds. Nesting dates shifted asymmetrically across the breeding season with differential changes in early, peak, and late nesting dates whereas long‐term changes were mediated by weather and geographic gradients. For example, nest initiation was earlier in warmer and wetter years. By applying a quantile‐based analysis, we provide estimates of the full nesting period that could serve as a flexible tool for scheduling monitoring efforts and balancing management and human activities with periods of reduced nesting activity. Our collaborative effort illustrates the challenges and opportunities of collecting long‐term data on avian communities necessary for answering questions across large regions but highlights the value of research coordination for bird conservation.

## Graphical abstract

Using > 170,000 nest records from 44 North American grassland bird species, we quantified how temperature, precipitation, and vegetation phenology shape early, peak, and late nesting across broad spatial gradients. Spatial predictions identify geographic variation in breeding phenology that can inform the timing of management actions under changing climates.

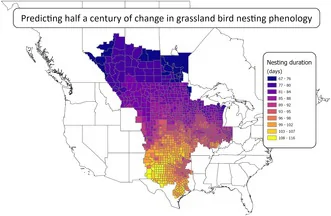

## 1 Introduction

Shifts in phenology are among the most widely observed biological responses to changing global climates (Cohen et al. 2018; Inouye 2022; Thackeray et al. 2016). The magnitude, direction, and ecological consequences of these phenological shifts vary across trophic levels and ecosystems, reflecting complex interactions between environmental drivers and species‐specific life‐history strategies (Cohen et al. 2018; Parmesan 2007). Phenological shifts can lead to desynchronization between a species' annual cycle events and its environment, and such mismatches have the potential to alter species interactions, demographic performance, and population trajectories (Miller‐Rushing et al. 2010; Visser and Gienapp 2019). In addition to ecological mismatches, phenological changes can undermine the effectiveness of natural resource management and conservation actions (Weiskopf et al. 2020). For example, shifts in phenology can affect when wildlife surveys are conducted, when seasonal restrictions or disturbance avoidance periods are implemented, and when habitat management activities are scheduled to minimize impacts on sensitive life stages. Further, shifts in breeding phenology could also have implications for seasonal wildlife regulations, including the timing of harvest seasons that are intended to minimize overlap with reproduction and maximize juvenile recruitment. Therefore, management practices based on historical phenological patterns may become misaligned with biological events if the timing of such events has shifted, potentially reducing the effectiveness of management or causing unintended negative consequences (Enquist et al. 2014; McGowan et al. 2021; Weiskopf et al. 2020). Despite well‐documented connections between phenology and climate change, implications of phenological shifts for natural resource conservation and management remain poorly understood, and implementing shifts in the timing of such activities may be an important component of climate adaptation (Bernath‐Plaisted et al. 2025).

A common approach in phenological studies is to characterize the onset or peak timing of key events (CaraDonna and Inouye 2015; Inouye 2022). However, species phenological responses are not always uniform and metrics such as the onset, peak, and duration of events may potentially shift across space and time or in response to changing climates at different rates or even in contrasting directions (CaraDonna and Inouye 2015; Neate‐Clegg and Tingley 2023). Variability in responses highlights the value of comprehensive approaches that examine a full distribution of phenological change rather than relying on isolated indicators (Inouye 2022). In addition, certain metrics, such as median or mean dates, may not fully capture relevant information about the timing of events if phenological patterns are skewed (e.g., due to infrequent breeding attempts occurring late in the season; Anteau et al. 2023). A complete understanding of phenological patterns would be particularly useful for informing management and conservation strategies, as managers often rely on estimates of the timing and duration of biological events to schedule monitoring efforts, identify periods when disturbance should be minimized, and determine when habitat management actions can be implemented to reduce risk to wildlife.

For species of conservation and management concern, identifying spatial and temporal trends in phenological patterns is increasingly important amidst challenges posed by changing environmental conditions. Breeding birds nesting in grasslands and associated shrubland habitats have experienced steep population declines in North America as a result of extensive habitat loss, degradation, and fragmentation of native prairies (Douglas et al. 2023; North American Bird Conservation Initiative 2025; Rosenberg et al. 2019; With et al. 2008). Grassland‐nesting birds breed in seasonal landscapes, where nest timing is expected to respond to vegetation phenology and annual weather conditions, yet the magnitude and direction of the relationships remain poorly quantified across the full breeding period and at broad geographic extents. Phenological flexibility in nest initiation within years may allow individuals to track local environmental conditions and could be adaptive if the nesting period maintains synchrony with peak resources (Bowers et al. 2016; McGowan et al. 2021; Messmer et al. 2021). However, long‐term directional shifts across time may facilitate adaptation as a reflection of sustained environmental change (Visser et al. 2012) or increase the risk of ecological mismatch if trophic interactions shift at different rates (Bates et al. 2023; Belitz et al. 2025). The timing of bird nesting has consistently been shown to influence reproductive success through its dependence on resource availability, exposure to extreme weather, predation risk, and overlap with anthropogenic disturbance (Grant and Shaffer 2012; Perlut et al. 2006; Zuckerberg et al. 2018). As a result, even subtle shifts in nesting phenology may have disproportionate demographic consequences (Both and Visser 2005; Reséndiz‐Infante and Gauthier 2020). Quantifying how the nesting period has changed across broad geographic and environmental gradients and over multiple decades is therefore useful for assessing population trends and informing conservation timing in declining grassland bird communities.

In grassland ecosystems, management practices such as prescribed fire, livestock grazing, and mowing are often deemed necessary for the economic productivity of working lands, or to mimic natural disturbance patterns, maintain forage productivity, prevent woody encroachment, control invasive plant species, and maintain habitat heterogeneity for grassland‐dependent species (Cutting et al. 2024; Fuhlendorf et al. 2006; MacDougall and Turkington 2007; Rinella and Hileman 2009; Shaffer and DeLong 2019). Additionally, grasslands are often managed for multiple objectives balancing activities like haying and cattle production with the habitat needs of grassland species. Such management practices are widespread in grassland ecosystems in the midcontinent of North America, where extensive conversion of native grasslands to croplands and the suppression of natural disturbance regimes from grazing by free‐ranging herbivores and wildfires have disrupted ecological processes that historically maintained diverse grassland composition and structure (Bernath‐Plaisted et al. 2023). The timing and frequency of grassland management practices can influence plant community responses (Rinella and Hileman 2009) and have implications for vertebrate species that breed in grasslands (Igl and Johnson 2016; Put et al. 2020). Grassland management actions during avian nesting seasons can negatively affect nesting birds by killing adults or young, destroying nests, reducing food availability, or altering vegetation structure in ways that affect reproductive success (Igl and Johnson 2016; Perlut et al. 2006; Wolcott et al. 2023). Agencies such as the US Farm Service Agency designate primary nesting seasons to guide implementation of conservation practices and agricultural management on working lands, while the Bureau of Land Management uses seasonal timing restrictions for activities including oil and gas development to minimize disturbance to breeding species, including greater sage‐grouse ( *Centrocercus urophasianus* ). Consequently, understanding the patterns and drivers of breeding phenology for grassland‐nesting birds is essential for balancing the timing of grassland management for maintaining agricultural production and the ecological integrity of grassland systems while minimizing harm to nesting birds.

Identifying phenological trends across species and regions necessitates extensive long‐term data collection, yet such datasets remain limited, particularly examples encompassing multiple species, habitats, and environmental gradients (Cohen et al. 2018). Phenological shifts often occur gradually and may only become detectable over multi‐decadal timescales (Parmesan 2007). Furthermore, phenological patterns within taxa exhibit spatial heterogeneity, complicating the extrapolation of localized trends to broader scales (de Keyzer et al. 2017). While the increasing availability of large‐scale data from remote sensing and community science platforms (e.g., USA National Phenology Network: https://www.usanpn.org) have improved researchers' ability to quantify broad patterns, obtaining information on the timing and duration of avian nesting is challenging because intensive field‐based data collection is required, and these data are rarely published (Anteau et al. 2023). Using a long‐term dataset of nest initiation dates, our objective was to evaluate temporal and spatial trends in nesting phenology for 44 species of grassland‐nesting birds over five decades across the midcontinental region of North America. A key prediction of our study was that environmental drivers would not influence all portions of the nesting season equally. Instead, we expected early and late nesting dates to exhibit distinct sensitivities to climate and phenological cues. We expected that early nesting dates would be more closely associated with spring temperature and timing of green‐up, which represent important environmental cues for the onset of breeding conditions (Forrest and Miller‐Rushing 2010). Conversely, we expected later nesting dates would show stronger associations with interannual weather variability, including precipitation, that may be associated with nesting success, renesting opportunities, and habitat conditions (Maresh Nelson et al. 2024; Saether et al. 2004). We further predicted that phenological responses would vary among species and across geographic gradients due to differences in seasonal climate and resource availability (Chmura et al. 2019; Tavera et al. 2024; Thackeray et al. 2016). Lastly, we predicted that nesting phenology would advance over time in association with long‐term changes in spring climate and advancing vegetation green‐up (Bates et al. 2023; Hällfors et al. 2020; Halupka and Halupka 2017), with stronger responses expected for the earliest portion of the nesting season. Our study provides contemporary estimates of early, median, and late nesting dates for 44 grassland bird species that can inform optimal timing of monitoring efforts and management strategies across the midcontinent of North America, such as avoiding disturbance under changing environmental conditions.

## 2 Methods

### 2.1 Data Pooling and Formatting

We pooled individual nesting datasets through coordination among hundreds of researchers and biologists who have completed field studies of grassland‐nesting birds. The effort resulted in a combined dataset of over 172,000 nest records that we used to evaluate patterns of grassland‐bird nesting phenology while allowing for species‐specific trends across time and environmental conditions. Nest initiation dates were collected by requesting data from grassland bird researchers and biologists as well as through an existing waterfowl nesting database (see Supporting Information for details). We assembled datasets for a suite of grassland bird species across North America collected between 1969 and 2023 using various data collection methods. Focal species in our study include several species of conservation concern, including at state, regional (e.g., Wightman et al. 2022), national (e.g., U.S. Fish and Wildlife Service 2021), and continental scales (North American Bird Conservation Initiative 2025). Data included species names, nest initiation dates (day that the first egg was laid), year, and approximate or site‐level geographic coordinates. In some cases, exact nest initiation dates were unknown or not recorded but either exact or estimated hatch dates or nest ages were known. If nest ages were recorded via egg flotation (Liebezeit et al. 2007) or candling (Lokemoen and Koford 1996), we back calculated from the dates when ages were measured to estimate nest initiation dates. If nests were located during the egg‐laying period, we assumed an egg‐laying rate of 1 egg per day to back calculate to nest initiation dates. If only hatch dates were known, we back calculated to nest initiation dates using species‐specific values of the average total length of the nesting cycle (inclusive of egg‐laying and incubation periods; table 1 in Anteau et al. 2023). We then converted all nest initiation dates to ordinal dates (e.g., January 1 = 1) to allow us to merge individual datasets (Figure S1 and Table S1).

### 2.2 Predictor Variables

To assess factors contributing to variation in nesting phenology, we derived predictor variables representing vegetation phenology and weather conditions. We used a measure of vegetation phenology based on the 2‐band Enhanced Vegetation Index (EVI) from the Moderate Resolution Imaging Spectroradiometer Land Cover Dynamics MCD12Q2 product, at 500 m spatial resolution (Friedl et al. 2022). We obtained MCD12Q2 layers from 2001 to 2022 and extracted the green‐up midpoint of the first growing cycle (mid green‐up day), representing the day of the year at which the EVI amplitude was estimated to be one‐half of its maximum. Mid green‐up dates have been used to describe the arrival of spring and the availability of resources for breeding birds and represent a spatially comparable index of spring phenology (Mayor et al. 2017; Robertson et al. 2024; Youngflesh et al. 2021). We were unable to use year‐specific estimates of mid green‐up dates because nesting dates in our dataset began in 1969 and MCD12Q2 layers are available starting in 2001. We instead calculated a climatological, contemporary (2001–2022) estimate of vegetation phenology using the median mid green‐up date across annual values. The metric captures spatial variation in the timing of vegetation phenology among locations and was included to explain variation in nesting phenology beyond that associated with broad geographic gradients (latitude and longitude). We excluded low‐quality mid green‐up values and only retained those that were ranked as “Best” or “Good” according to the MCD12Q2 quality‐assurance ranking system. We extracted the long‐term mid green‐up date at nesting site coordinates. We additionally included longitude and latitude as predictor variables to account for differences in nesting phenology across spatial gradients that may not be fully captured by long‐term green‐up dates. Elevation can additionally influence nesting phenology through local effects on temperature and vegetation phenology. However, given the limited elevational variation in our dataset and the inclusion of green‐up dates as a direct measure of vegetation phenology, we did not include elevation as a separate predictor in the analysis.

To generate predictor variables characterizing annual weather conditions, we acquired TerraClimate layers (1958–2023) which included monthly temperature and precipitation accumulation at a 4‐km resolution (Abatzoglou et al. 2018). We averaged weather layers across months (March–June) as indices of broad‐scale spring conditions that may influence nesting phenology while maintaining comparability among species with different nesting schedules and across the north–south gradient of the study area. We then quantified interannual weather variability by detrending gridded temperature and precipitation time series at the pixel level. For each 4‐km pixel, we fit a linear regression of annual weather values by year (1958–2023) and extracted the residuals. The residuals represent deviations from each pixel's long‐term mean and temporal trend, thereby decomposing spatial differences in baseline climate and local long‐term trends. Detrended temperature and precipitation values were extracted at nest locations for each corresponding year. The anomaly terms were interpreted as within‐location, interannual variation in weather conditions and the estimated effects reflect phenological responses to unusually warm/cool or wet/dry years relative to expectations for a given location and the long‐term trajectory. We therefore included year as a predictor variable to evaluate long‐term trends in nesting phenology. Despite differences in spatial scale and temporal extents of mid green‐up and weather predictors, we are confident that scaling dissimilarities do not meaningfully bias inference because weather covariates were expressed as interannual variation within sites and the broad spatial and multi‐decadal temporal scope of our nesting dataset. The covariate for year was centered at 1996 (the midpoint between 1969 and 2023) and scaled by 10 years, such that slope coefficients represent the estimated change in nest initiation date per decade. All other covariates were scaled to have a mean of 0 and standard deviation of 1. Mid green‐up dates and longitude were moderately correlated with latitude (*r* = 0.50 and 0.52, respectively), but all predictors were retained in the models as none had correlation values > |0.6|. In addition, we assessed multicollinearity among predictor variables by fitting a model with only fixed effects and calculating variance inflation factors (VIF). All predictor variables had VIF values < 2, indicating low collinearity among covariates (Graham 2003). A summary of predictor variables is provided in Table S2.

### 2.3 Analysis

We used quantile regression to evaluate spatial and temporal patterns in nest initiation dates across species. Quantile regression provides a flexible approach to modeling the relationship between predictor variables and different points of the response distribution (nest initiation dates in our case), rather than focusing solely on a single statistic such as the mean of a response (Barton and Sandercock 2018; Cade and Noon 2003; Williams et al. 2023). Quantile regression is particularly useful for analyzing long‐term changes in phenology, as environmental factors may have different effects on the beginning and end of key periods for seasonal life‐history events. We implemented the analysis in a Bayesian setting which minimizes an asymmetric loss function, which is useful when response variables are skewed or contain long tails (Williams et al. 2023; Yu and Moyeed 2001). To assess different portions of nesting phenology distributions and evaluate if environmental factors differentially influenced early and late nesting species, we fit seven identical quantile regression models, corresponding to seven quantiles, *τ*, of nesting initiation dates (0.05, 0.10, 0.25, 0.50, 0.75, 0.90, and 0.95). For example, setting *τ* = 0.95 yields predictions for the date when 95% of nests have been initiated. We chose these seven quantiles to characterize the full nesting distribution while avoiding instability associated with modeling more extreme tails. Quantiles were estimated across years and species as conditional quantiles of nest initiation dates given annual and spatial predictor variables described above, rather than calculated separately within each breeding season. Use of quantiles allowed us to evaluate long‐term shifts in different portions of the nesting season and assess whether phenological changes were uniform across the nesting period.

Our general strategy was to model all species together, but with species‐specific intercepts and slopes. Joint modeling allowed for inference on general patterns across all species, while accounting for differing phenologies and responses to predictor variables among species. To avoid overfitting, we restricted species‐specific slope estimation to species with at least 200 total nest records. Bird species with fewer than 200 nest records were included in the analysis with shared global slopes. This approach allowed rare or sparsely studied species to contribute information to overall trends without introducing instability due to insufficient data. We ran seven models setting the skew parameter, *τ*, at 0.05, 0.10, 0.25, 0.50, 0.75, 0.90, and 0.95 to estimate quantiles of nest initiation dates. Nest initiation dates $y_{\mathrm{ist}}$, for nest *i*, species *s*, and year *t*, were modeled as a random variable following the asymmetric Laplace distribution (ALD; Yu and Moyeed 2001), $y_{\mathrm{ist}}\sim \mathrm{ALD}\left(\mu_{\mathrm{ist}}^{\left(\tau \right)},\sigma_{\tau },\tau \right)$, where $\sigma_{\tau }$ is the scale parameter. $\mu_{\mathrm{ist}}^{\left(\tau \right)}$ represents the *τ*th conditional quantile of initiation dates and was modeled as a linear function of temperature, precipitation, year, mid green‐up dates, latitude, and longitude:

$$\begin{array}{c} \mu_{\mathrm{ist}}^{\left(\tau \right)}=\varepsilon_{s}^{\left(\tau \right)}+\beta_{1,s}^{\left(\tau \right)}\cdot \text{Temperature}+\beta_{2,s}^{\left(\tau \right)}\cdot \text{Precipitation}+\beta_{3,s}^{\left(\tau \right)}\cdot \text{Year}\\ +\beta_{4,s}^{\left(\tau \right)}\cdot \text{Greenup}+\beta_{5,s}^{\left(\tau \right)}\cdot \text{Latitude}+\beta_{6,s}^{\left(\tau \right)}\cdot \text{Longitude}. \end{array}$$

To estimate species‐specific intercepts $\varepsilon_{s}^{\left(\tau \right)}$, and species‐specific slopes, $\beta_{1,s}^{\left(\tau \right)}$, $\beta_{2,s}^{\left(\tau \right)}$, $\beta_{3,s}^{\left(\tau \right)}$, $\beta_{4,s}^{\left(\tau \right)}$, $\beta_{5,s}^{\left(\tau \right)}$, and $\beta_{6,s}^{\left(\tau \right)}$, for temperature, precipitation, year, mid green‐up date, latitude, and longitude, respectively, we used non‐centered parameterizations: $\beta_{s}=\beta +z_{s}\cdot \sigma_{\mathrm{\beta s}}$, $z_{s}\sim N\left(0,1\right)$ for slopes and $\varepsilon_{s}=\alpha +z_{s}\cdot \sigma_{\varepsilon }$, $z_{s}\sim N\left(0,1\right)$ for intercepts. This parameterization improves sampling efficiency and posterior stability (Monnahan et al. 2017), particularly because sample sizes among species were unequal. Overall slopes, β, represent average effects across species, while $\sigma_{s}$ captures the magnitude of variation in predictor effects among species. We used weakly informative normal priors for all global slopes, β, with mean 0 and standard deviation 10, which provides mild regularization by constraining effect sizes to biologically plausible ranges relative to the length of the nesting season while remaining diffuse enough to allow the data to dominate inference. For the variance parameter priors $\sigma_{\mathrm{\beta s}}$ and $\sigma_{\varepsilon }$, we used a gamma distribution with shape 2 and rate 0.1 that places most prior mass on moderate values but has a right‐skewed tail allowing larger among‐species differences if supported by the data. This prior reflects the expectation that species differ in their phenological responses to environmental conditions, while avoiding overly restrictive constraints on interspecific variation. We used a weakly informative normal prior for the overall intercept, α, with means corresponding with observed quantiles in the data (5% = 26 April, 10% = 4 May, 25% = 15 May, 50% = 28 May, 75% = 9 June, 90% = 20 June, and 95% = 27 June) and standard deviation of 15 days to allow wide but biologically plausible values.

We quantified distributional asymmetry for each species using a quantile‐based measure of skewness. For each posterior predictive distribution of nesting dates, we calculated skewness as $\left(\varepsilon_{s}^{\left(0.75\right)}-2\varepsilon_{s}^{\left(0.50\right)}+\varepsilon_{s}^{\left(0.25\right)}\right)/\left(\varepsilon_{s}^{\left(0.75\right)}-\varepsilon_{s}^{\left(0.25\right)}\right)$ (Ekström and Jammalamadaka 2012), where positive values indicate right‐skew with more early nesting dates whereas negative values indicate left‐skew with more late nesting dates. Last, we evaluated the sensitivity of our results to the predominance of waterfowl records in the combined dataset by repeating the analyses after excluding all waterfowl species and refitting models for early (*τ* = 0.05), median (*τ* = 0.50), and late (*τ* = 0.95) nesting quantiles.

We constructed models using the software Stan (Carpenter et al. 2017) via the *CmdStanR* package (Gabry et al. 2025) in R (R Core Team 2025). See Supporting Information for a more detailed explanation of the models, evaluation of model fit, and pseudo‐code describing the implementation of the ALD. Posterior predictive checks indicated that our models were well‐calibrated across all seven quantile levels for all species and years, with the proportion of observed values below the predicted values closely matching *τ* values (0.05 for *τ* = 0.05, 0.11 for *τ* = 0.10, 0.26 for *τ* = 0.25, 0.49 for *τ* = 0.50, 0.74 for *τ* = 0.75, 0.89 for *τ* = 0.90, and 0.94 for *τ* = 0.95; Figure S3). We present posterior medians and 90% Bayesian credible intervals.

### 2.4 Predicting Nesting Dates in Midcontinent Grasslands

We used quantile regression models to make predictions across space describing grassland bird nesting seasons. We were interested in making predictions about the start of nest initiation as well as potential end dates, and therefore we added species‐specific values of the total duration of a nesting cycle including the egg‐laying, incubation, and nestling periods (Table 1 in Anteau et al. 2023) to the 50th, 75th, 90th, and 95th percentiles of predicted nest initiation dates. We considered end dates as the latest date when the stated percentages of initiated nests would be finished. Estimated end dates do not account for failed nests that would not reach hatching or fledging, so we are not interpreting this date as the completion date for unsuccessful nests. Double‐brooding and renesting attempts were not modeled separately, and late‐season predicted dates reflect a mixture of all nesting attempts and therefore represent overall nesting activity toward the end of the season rather than first‐nest end dates. We generated predictions including dates when 5%, 10%, 25%, and 50% of nests were initiated and maximum end dates for 50%, 75%, 90%, and 95% of nests. We calculated the potential nesting duration for each species using the number of days between the 5th percentile of nest initiation and 95th percentile of potential end dates.

The spatial unit and extent of predictions were US counties and Canadian census divisions (hereafter referred to as units) within midcontinent grasslands of North America (MacDonald et al. 2024) defined using seven Bird Conservation Regions associated with grassland ecosystems (North American Bird Conservation Initiative 2014). Within each unit, we calculated the median mid green‐up date (from 2001 to 2022) and the latitude and longitude of the unit centroid. We predicted nesting season dates in each unit under variable weather conditions using the linear predictor described above. Specifically, we calculated unit‐level dates when minimum temperature deviations were average, cool (1.5°C below average), and warm (1.5°C above average). We standardized unit‐level predictor values using the same global means and standard deviation applied during model fitting to ensure consistency between model‐estimated coefficients and predictor values when projecting nesting dates across space. If 95% credible intervals for species‐specific random slopes overlapped zero, we did not include that additional effect in the predictions. Last, predictions were referenced to the year 2023 so that projected nesting dates reflect contemporary conditions rather than historical averages.

To generate overall predictions of nesting phenology and exclude species‐specific predictions of phenology in units where species do not occur, we used relative abundance products from the eBird Status and Trends project (Fink et al. 2024). We retrieved relative abundance maps for each species using predictions for 2023 via the *ebirdst* package (Strimas‐Mackey et al. 2023) in R. Within each unit, if a species' relative abundance was predicted to be > 0 during the species' breeding season, we included that species' phenology estimates in the unit predictions. This approach does not account for species abundances; therefore, predictions of overall nesting phenology treat estimates from rare and abundant species equally rather than proportional to their relative spatial abundance. We also restricted predictions by taxonomic group to states or provinces with more than one nest per group in our dataset to avoid extrapolating outside of areas where data collection was concentrated (Figure S1). Last, we combined species‐level predictions at the county level into taxonomic groups to allow for generalizations across broad taxonomic groups in conservation and management applications.

## 3 Results

Our total dataset included 172,158 nest initiation dates for 44 avian species representing waterfowl (8 spp., 73% of nesting data), passerines (20 spp., 20% of nesting data), upland gamebirds (7 spp., 6% of nesting data), shorebirds and a heron (7 spp., 1% of nesting data), and raptorial birds (2 spp., < 1% of nesting data; Figure S1). We included data collected between 1969 and 2023 in our analysis (Figure S1 and Table S1). We did not estimate species‐specific responses for species with < 200 nest records, but their data contributed to overall responses (*n* = 7; Table S1). Most species (77%) had > 20 individual years of nest records (median = 27.5 years, range = 7–55 years) covering multiple US states and Canadian provinces (median = 7 states or provinces, range = 3–17 states or provinces).

### 3.1 Overall Patterns of Nest Timing

The overall estimated period of nest initiation across all species ranged from 5 May (*τ* = 0.05) to 1 July (*τ* = 0.95). The estimated scale parameter varied across quantiles, with the greatest values observed at the median (*τ* = 0.50) and lower values at both extremes (*τ* = 0.05 and 0.95; Table S3). This pattern indicates that estimated nest initiation dates were more variable in the middle of the breeding season, becoming increasingly concentrated toward the earliest and latest portions of the nesting window.

Across all species, shifts in nest timing across the 55‐year study period showed quantile‐dependent patterns where the global slopes were negative at lower quantiles (*τ* = 0.05–0.25) and positive at upper quantiles (*τ* = 0.75–0.95). Initiation dates of the earliest 5% of nests advanced through time at a rate of 1.64 days per decade (90% CI = 0.81–2.50; 100% of posterior distribution < 0), whereas initiation dates for the latest 5% of nests were delayed at a rate of 0.62 days per decade (CI = −0.31 to 1.56; 86% of posterior distribution > 0), indicating a widening of the nest initiation period (Figure 1A,B). Across our entire study period (1969–2023), early nests (*τ* = 0.05) advanced by 8.9 days (CI = 4.4–13.5) and late nests (*τ* = 0.95) were delayed by 3.4 days (CI = −1.7 to 8.4), resulting in an expansion of the nest initiation period (days between 5% and 95% of nests initiated) from 43.3 days (CI = 35.1–51.8) in 1969 to 55.6 days (CI = 48.0–63.3) in 2023 (Figure 1B).

**FIGURE 1:**
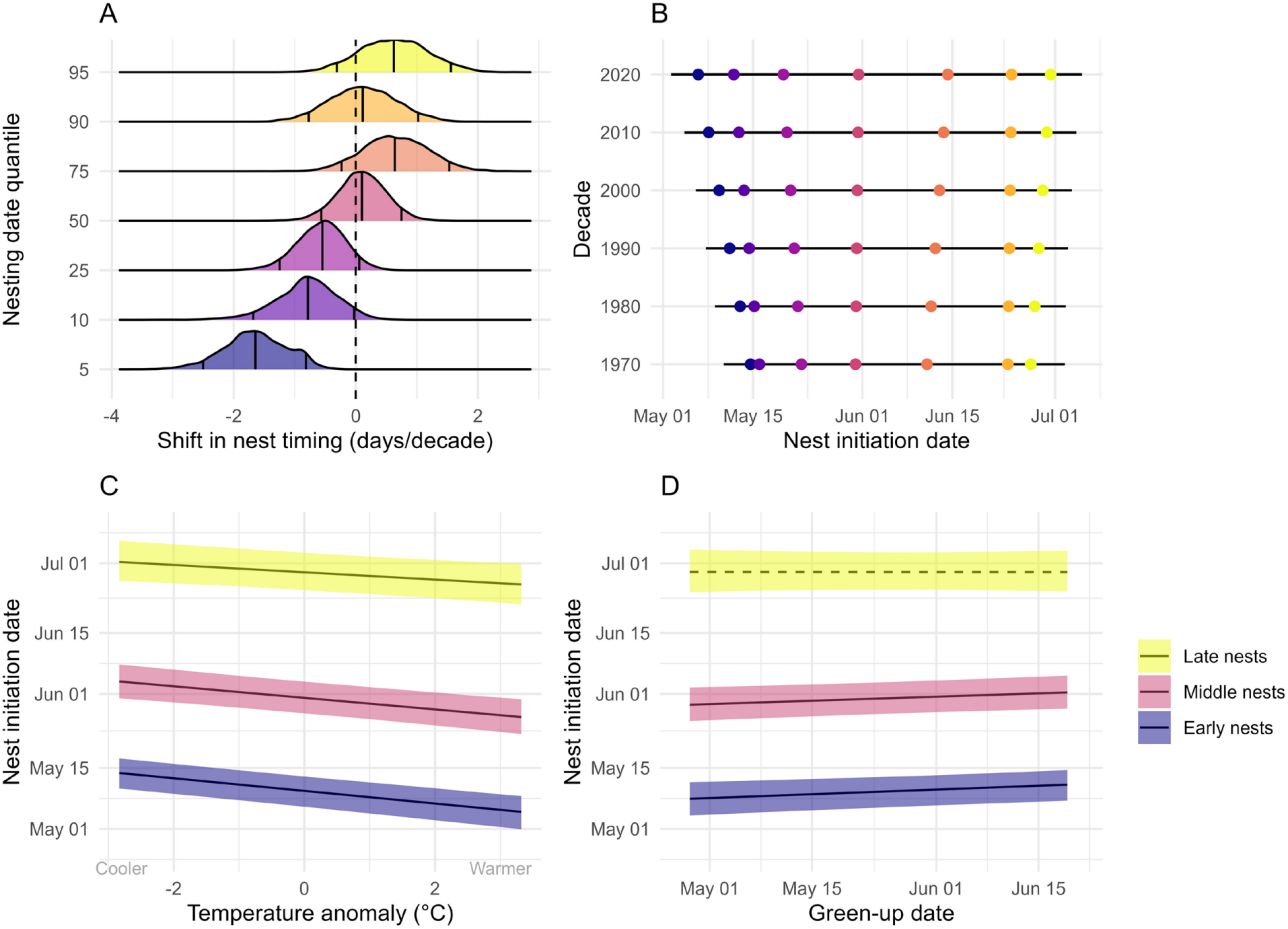
Quantile‐specific global shifts and environmental relationships with grassland bird nest timing. Posterior distributions in Panel A illustrate decadal shifts in nest initiation dates across nesting date quantiles (days per decade) with vertical bars showing medians and 90% credible intervals. Negative values indicate earlier nest initiation, positive values indicate later nest initiation, and the dashed vertical line at zero indicates no temporal shift. Panel B presents estimated nest initiation dates (median ± 90% credible intervals) across decades for seven quantiles (*τ* = 0.05, 0.10, 0.25, 0.50, 0.75, 0.90, and 0.95 from left to right). Panels C and D show predicted relationships between nest initiation dates and spring temperatures (°C deviation from the long‐term mean; Panel C) and green‐up timing (Panel D) for early (*τ* = 0.05), middle (*τ* = 0.50), and late (*τ* = 0.95) nests. The dashed line in Panel D indicates no effect as 90% credible intervals overlapped 0. Partial predictions were derived using the full distribution of posterior estimates of the overall intercept and predictor effects, while holding other covariates at their mean values and show mean effects with 90% credible intervals.

Temperature and precipitation in spring were both predictors of nest initiation date, with nests initiated earlier in warmer and wetter years across all quantiles, and temperature had a greater and more consistent influence across nesting dates than precipitation (Figure 1C and Table S3). Temperature effects were greater at lower quantiles (Figure 1C), indicating spring temperatures exerted a stronger influence on early nests than on clutches initiated later in the season. For example, when temperatures were 2°C above average, early nests (*τ* = 0.05) were 5.8 days (CI = 4.68–7.03) earlier on average than when temperatures were 2°C below average, whereas late nests (*τ* = 0.95) were 3.4 days (CI = 1.1–5.5) earlier under those same conditions. Conversely, precipitation effects were weaker at the tails and stronger during the middle of the nesting season (Table S3).

As expected, nest initiation dates were later at higher latitudes and when mid green‐up dates were later (Figures 1D and 2). Accordingly, the duration of the nesting season (5% of nests initiated to 95% of nests complete) decreased from south to north (Figure 2). Latitude had a greater effect on nest initiation dates than mid green‐up dates across all quantiles; however, the magnitude of latitude effects decreased at later portions of the nesting season (Table S3). Longitude had a positive effect on nest timing during the middle of the nesting season, indicating that median nest initiation dates were later in the east and earlier in the west. Longitude had only a weak negative effect on the timing of the end of the nesting season (Figure 2 and Table S3).

**FIGURE 2:**
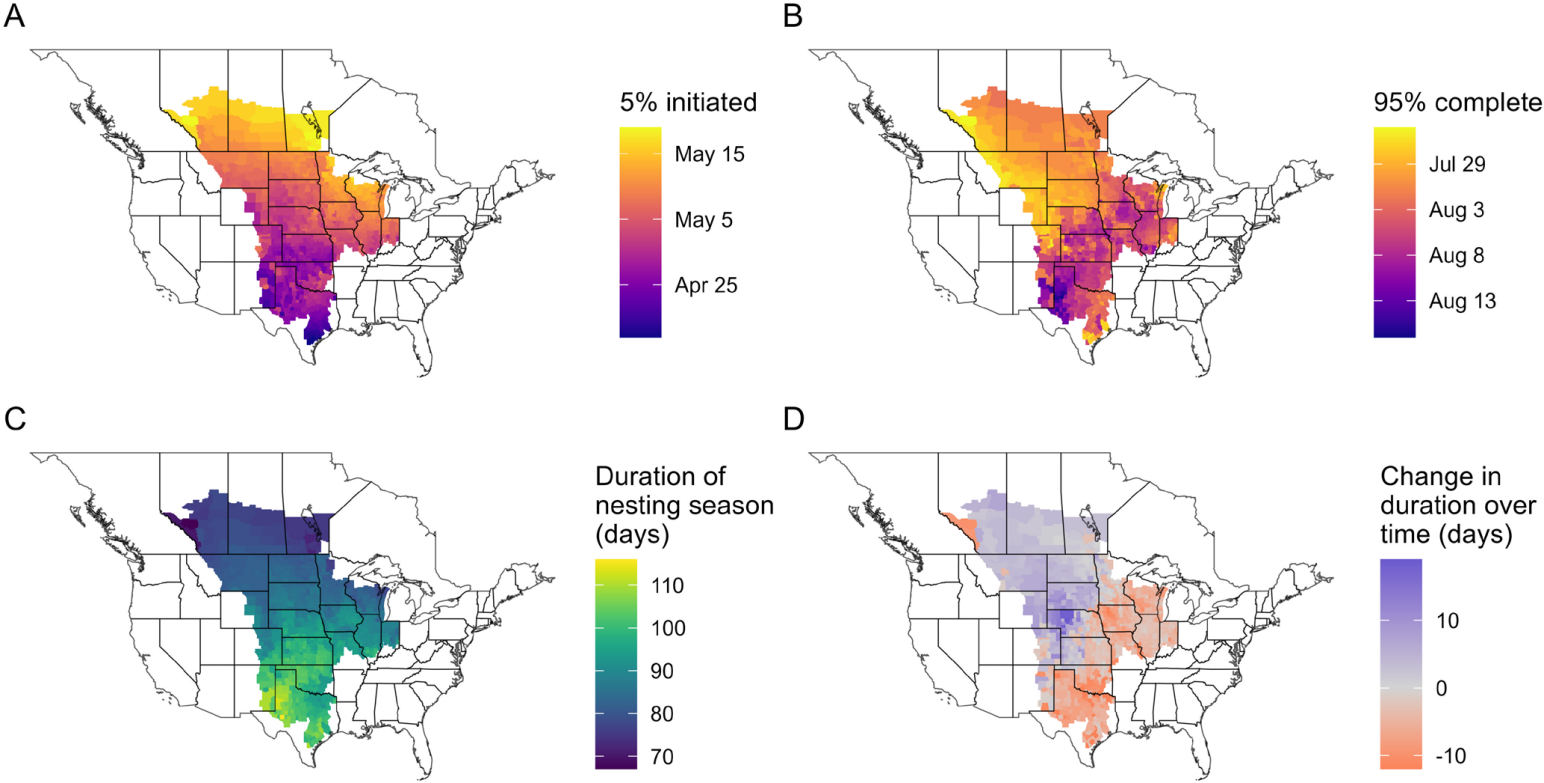
Predictive maps of nesting phenology in North American midcontinent grasslands across all bird species showing mean dates when 5% of nests were initiated (A), dates when 95% of initiated nests would be complete, based on mean nesting cycle length of successful nests (B), nesting season duration (C), and change in nesting season duration from 1969 to 2023 (D). Duration of nesting season was calculated as the number of days between the 5% and 95% quantiles (A, B).

Our total dataset was dominated by information from waterfowl, but restricting our analyses to only data from the remaining groups produced global parameter estimates that remained consistent with those from the full dataset (Table S4), indicating that community‐level patterns were robust to the taxonomic composition of the dataset. For example, initiation dates of the earliest 5% of non‐waterfowl nests advanced through time at a rate of 2.0 days per decade (CI = 1.0–3.0), whereas initiation dates for the latest 5% of nests were delayed at a rate of 0.6 days per decade (CI = −0.5 to 1.8; Table S4).

### 3.2 Species‐ and Group‐Specific Patterns of Nest Timing

Phenological responses showed substantial species‐specific variation across time, with species often differing in both the direction and magnitude of estimated responses (Figure 3). We lacked sufficient data to estimate species‐specific responses for seven species with < 200 nests (Table S1), including the single heron and both raptor and owl species. Waterfowl and shorebird species were generally consistent in advancement of early nesting (median responses = −0.3 and −2.7 days per decade for waterfowl and shorebirds for *τ* = 0.05) with more species indicating advancement (e.g., 3 of 5 shorebird and 4 of 8 waterfowl species for *τ* = 0.05; Table S5). Initiation dates for early nests trended later for most passerine and upland gamebirds (11 of 18 passerine and 4 of 6 upland gamebird species with median responses = 0.9 and 1.2 days per decade for *τ* = 0.05; Figure 3B). At later stages of the nesting season (*τ* = 0.95), waterfowl nesting dates consistently shifted later over time (e.g., 88% of species had positive slopes and median response = 1.2 days per decade), the remaining groups showed mixed responses in late nesting dates among species. For example, of the five shorebird species, two shifted later and one shifted earlier over time for *τ* = 0.95. Half of the passerine species (9 of 18) shifted earlier while three species shifted later. Lastly, among six upland gamebird species, two shifted later and three shifted earlier over time.

**FIGURE 3:**
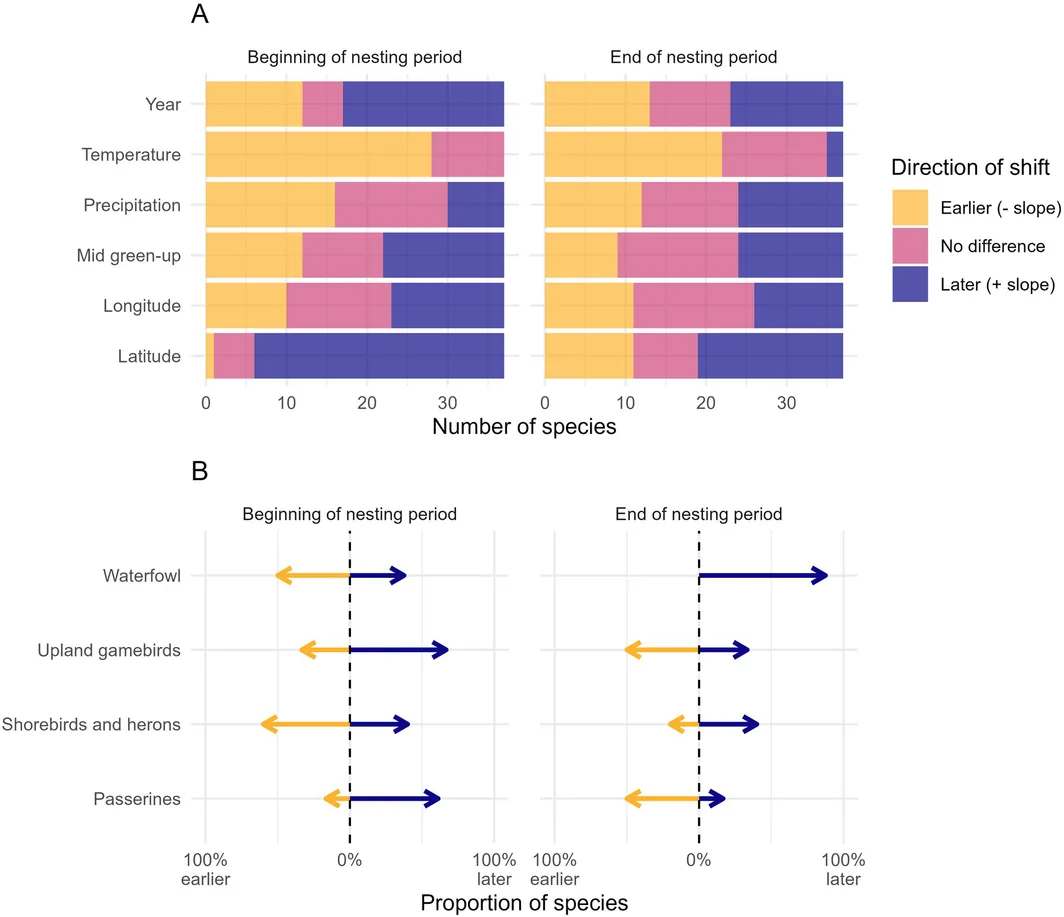
Counts of species‐specific responses for six predictor variables at the beginning (*τ* = 0.05) and end (*τ* = 0.95) of the nest initiation period across all species (Panel A). For each predictor variable, bars indicate the number of species with earlier nesting (orange; negative slopes), no effect (pink; 90% credible intervals overlapping zero), or later nesting (purple; positive slopes). Directional arrows represent the proportion of species within each bird group with earlier (negative slopes; orange arrows) or later (positive slopes; purple arrows) shifts in nest initiation over time (i.e., year effect) at the beginning and end of the nest initiation period (Panel B).

The direction and magnitude of species‐level responses were relatively consistent across gradients of latitude and temperature, particularly for early nests (Figure 3A). Within species, nesting dates were generally later at higher latitudes and earlier with warmer temperature anomalies. In addition, temperature and latitude had the lowest number of non‐directional effects (90% CI overlapped zero) among predictor effects, reflecting stronger signals across multiple species (Table S5). Most species also aligned with global trends of earlier nesting in wetter years (Table S5). However, waterfowl nesting dates deviated from these patterns by shifting later at upper quantiles in wetter years where 88% of species had positive slopes for *τ* = 0.90 and 0.95. Along the longitudinal gradient of our study area, 38% of species were consistent with global trends of later median nest initiation dates in the east and earlier in the west, whereas 27% showed the opposite response. Notably, waterfowl nesting dates were later in the east and earlier in the west throughout the nesting season. Last, species‐level responses to mid green‐up dates consistently leaned positive, indicating that later green‐up generally was associated with later nesting, especially by the middle of the nesting season when 54% species had positive slopes.

Species varied in their predicted nest initiation dates, and the breadth between early (*τ* = 0.05) and late (*τ* = 0.95) quantiles indicated differences in the overall length of species' nesting periods (Figure 4). At a group level, the nest initiation period (number of days between *τ* = 0.05 and *τ* = 0.95) was more synchronous for shorebirds and a heron species (median = 35 days) and longer for raptorial birds and upland gamebirds (median = 62 and 61 days; Figure 4). The length of the nest initiation period at a species level was more strongly correlated with late nesting dates (*r* = 0.68) than early nesting dates (*r* = −0.34), indicating that differences in the nest initiation period among species were driven primarily by variation in the timing of the latest nesting attempts. Distributions of nest initiation dates were approximately symmetrical or right skewed for most species (mean skew coefficient across all species = 0.16), meaning many nests were initiated before the average nest initiation date due to long tails of initiation dates for later nests (Figure S2).

**FIGURE 4:**
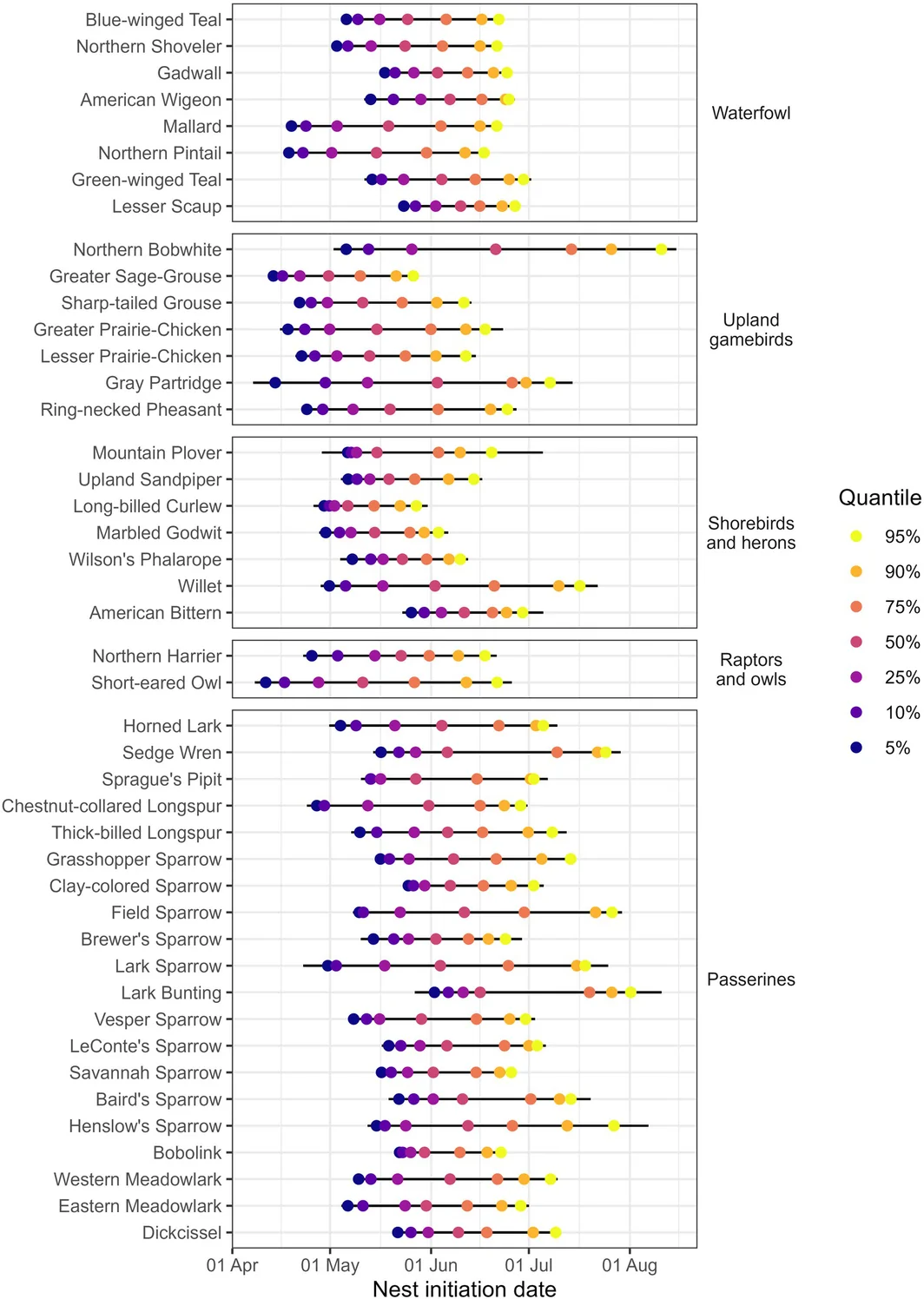
Posterior estimates of species‐specific nest initiation dates across seven quantiles (*τ* = 0.05, 0.10, 0.25, 0.50, 0.75, 0.90, and 0.95). Points represent posterior means with 90% credible interval lines. Estimates were derived using species‐specific mean values for latitude, longitude, and mid green‐up effects. These estimates represent contemporary nesting timing within each species' typical geographic distribution under average weather conditions (where both temperature and precipitation was set to 0 and year was set to 2023). Species are listed in taxonomic order and organized by taxonomic groups (Chesser et al. 2025).

## 4 Discussion

Our results reveal clear patterns of variation in nesting dates with implications for the ecology and management of declining grassland birds across midcontinent North America. A novel finding of our collaborative study was that different stages of the nesting season exhibit distinct responses to environmental gradients and long‐term changes. We evaluated the full distribution of timing of nesting for 44 species over more than five decades and discovered asymmetric shifts that would be overlooked by focusing solely on peak or mean nesting dates. The work also informs a predictive model and web tool that can be used to plan monitoring and research activities and better inform decisions about timing grassland management actions in such a way to reduce disturbance to nesting birds (U.S. Geological Survey 2026). Our modeling framework has the capacity to predict nest initiation and completion timing under variable weather conditions associated with warmer or wetter spring conditions, which enables more flexible and condition‐specific scheduling of management activities that will be better aligned with local nesting phenology.

Understanding the effects of environmental change on grassland bird nesting phenology is critical for climate adaptation of management and conservation programs, spatial prioritization of conservation efforts, and future‐focused projections of population trends. We found asymmetric shifts in nest initiation timing across species and years, indicating that not all parts of the nesting season change equally in response to environmental variability. Shifts in phenology can also have important demographic consequences. For instance, early‐season nests are often found to contribute disproportionately more to annual reproductive output in waterfowl (Dzus and Clark 1998; Elmberg et al. 2005; Krapu et al. 2000) and multi‐brooded species of songbirds (Hochachka 1990); therefore, changes in the timing or success of early nests may have greater effects on population trajectories. Phenological shifts may also alter interactions with brood parasites if host and parasite nesting phenologies change at different rates (Boldrick et al. 2026). We found that the timing of early nests responded more strongly to temperature than late nests. Both temperature and precipitation contributed to variation in nesting phenology and have also been linked to changes in demographic rates of grassland‐nesting birds (Maresh Nelson et al. 2024). Our analysis revealed subtle shifts in the earliest and latest portions of the nesting season across time, which clarifies findings from a recent meta‐analysis where long‐term changes in peak fledging dates were not detected across 36 grassland‐nesting bird species (Anteau et al. 2023). However, the meta‐analysis was restricted to mean and median dates due to limited reporting in the literature and was unable to consider trends in early and late nesting that were evaluated in the current study. Although the community‐wide (global) effect of year in our analysis indicated earlier nest initiation of early nests, many species‐specific responses indicated later initiation. Our results indicate that heterogeneous species responses can occur around a common community trend, reflecting the hierarchical weighting of the model where a global pattern is estimated directly from all nesting data across species and reflects a trend of community‐wide nesting dates, rather than an approximate average of species‐specific slopes. Community‐wide nesting patterns at the lowest quantiles are driven by species with abundant early nests that may be more sensitive to early‐season environmental shifts. Therefore, species‐level slopes capture within‐species temporal changes, whereas the global trends capture community‐level advancement in the earliest nesting dates and delaying of the latest nesting dates. Importantly, the removal of all waterfowl nest records (73% of the combined dataset) produced similar community‐level parameter estimates indicating that our main conclusions about shifts in nesting phenology were shared across waterfowl and non‐waterfowl taxa. Because temperature and precipitation were detrended prior to analysis, year effects capture long‐term temporal change independent of interannual weather variability. In contrast, temperature and precipitation capture annual weather anomalies, while green‐up represents spatial variation in spring phenology. Consequently, year effects likely integrate multiple, unmeasured processes, including long‐term environmental change, land use change, and population dynamics. As a result, the global effect of year may mask heterogeneity across species, with some responding primarily to weather cues and others influenced by other ecological or demographic constraints. Further, the greater conditional variability and spread of nesting dates around the median nesting period may reflect broader species heterogeneity or flexibility in mid‐season nesting dates, when weather and habitat conditions are likely to be more favorable, and more nests can be initiated. In contrast, the reduced dispersion at the earliest and latest quantiles likely reflects stronger ecological constraints on the timing of nest initiation, such as temperature limitations early in the season or declining habitat quality or daylight hours, and seasonal constraints on fledging success later in the season (Krapu et al. 2000; Raquel et al. 2019).

Our findings align with established spatial patterns in nesting phenology, with birds nesting progressively later at higher latitudes across North America. The effect of latitude was more pronounced on early and peak nest initiation dates, with reduced effects on late nests, potentially indicating that the end of the nesting period is more constrained by seasonal resource decline, molt timing, or the need to complete breeding before migration, even at lower latitudes. A latitudinal gradient in nest timing is consistent with broad‐scale variation in climate and vegetation phenology. Green‐up is often used as a proxy for resource availability (Mayor et al. 2017; Youngflesh et al. 2021), and recent studies indicate that migration timing in many bird species may be more strongly related to long‐term average green‐up patterns than to interannual variation, resulting in potential mismatches in warm or early springs (Mayor et al. 2017; Robertson et al. 2024). In contrast, Arctic breeding birds advanced their arrival across time and in response to earlier snowmelt, but egg‐laying dates were less responsive to temporal shifts among species (Lameris et al. 2025). While we did not test relationships between nesting phenology and interannual variation in green‐up dates, we found that nest initiation timing was responsive to annual weather conditions, indicating grassland birds may align the onset of nesting with environmental cues including temperature or vegetation growth. This distinction highlights an ecological nuance that although certain migratory species may arrive to breeding areas based on fixed migratory schedules tied to photoperiod and historical seasonality, nesting decisions may retain some flexibility to adjust to local weather conditions after accounting for the broad geographic constraints of latitude. However, our results revealed that this flexibility was not uniform across species or across the entire breeding season, indicating varying degrees of phenological sensitivity to spatial and temporal changes in spring phenology. Predicting these patterns is essential for anticipating where and when mismatches between bird reproduction and resource availability are most likely to occur.

The widening of the nesting phenology distribution across the community of species in our study, where the earliest and latest nests occurred farther apart over time while median nest initiation dates remained relatively unchanged, could have potential consequences for life‐history variation. Extended periods of favorable environmental conditions may increase opportunities for multiple brooding or renesting attempts after failure, potentially increasing reproductive output (Arnold et al. 2010). However, longer breeding seasons may also alter species interactions or energetic costs of prolonged reproductive effort (Barve et al. 2024; Fletcher Jr. et al. 2024). Individuals initiating nests earlier may experience greater negative effects from extreme weather events occurring in early spring (Maresh Nelson et al. 2024) but could potentially benefit from reduced competition for nest sites and optimal resource peaks during warmer springs (Blums et al. 2002). Our results revealed spatial heterogeneity in long‐term changes in nesting duration, with nesting seasons lengthening in waterfowl‐dominated regions but shortening in areas where passerines and upland gamebirds are more prevalent. The observed pattern contrasts with findings of widespread advances in breeding onset yet reduced breeding duration for about one‐third of 73 boreal bird species in Finland (Hällfors et al. 2020). Together, these two studies demonstrate that the duration of nesting seasons can vary markedly across space, with local community composition shaping how phenological windows lengthen or contract in response to changing environmental conditions.

Our findings raise several key research gaps to inform the optimal timing of management and demographic consequences of climate‐driven changes in nest timing including an understanding of long‐term changes in the contributions of early versus late nests to annual production, seasonal nesting intensity measured as the number of active nests per day, and frequency and reproductive value of renesting or double brooding across the community of grassland‐nesting birds. Further, variation in phenological plasticity across species and populations may lead to reduced interspecific competition or changes in predator pressure. These dynamics could complicate conservation and management decisions about when to conduct potentially disruptive activities. Understanding not just the phenology of when birds nest but which portions of the nesting season are most productive could collectively help managers prioritize periods of greater sensitivity.

As with most field studies on nesting birds, our data and results were constrained by the timing and duration of field efforts, which varied across studies, sites, and years. Nesting studies generally aim to identify all nests throughout the nesting season. However, financial and logistical challenges could limit field effort at the beginning and end of the nesting season so that the earliest and latest nests go unmonitored, resulting in an underestimate of the full duration of the nesting season. The extent that the true first and last nests of a given nesting season are found was unclear as few studies explicitly include full temporal coverage as an objective. For these reasons, we focused on the core portion of the nesting season, between 5th and 95th percentiles, which we think reduces sensitivity to variation in timing of field work. In addition, longer‐surviving nests are more likely to be detected, which may introduce bias in nest timing data if survival rates change during the season. Due to limited information from individual studies, we could not address this issue. The extent and direction of such bias may vary because seasonal trends in nest survival often differ among species and depend on site‐specific habitat conditions (Davis et al. 2016; Emery et al. 2005; Grant and Shaffer 2012). Although the use of a large, aggregated dataset provides broad inference across taxa and regions, uneven representation among species represents an important source of uncertainty. Because waterfowl comprised the majority of nest records, we evaluated whether overall patterns were sensitive to their disproportionate contribution. We found that our results were consistent in magnitude and direction after censoring waterfowl records from the data, suggesting that our conclusions were not solely driven by this group. However, taxonomic differences in phenological responses remain an important consideration when interpreting aggregated trends. Last, while our analysis included data on weather, green‐up dates, latitude, and longitude as drivers of phenological patterns, we did not incorporate land use or vegetation types. Habitat quality can also influence nest initiation timing and nesting habitat suitability (Greenwood et al. 1995; Pearse et al. 2022; Swicegood et al. 2023), potentially weakening or confounding spatial relationships in nesting patterns we observed. However, we have no information that these biases have changed through time in a way that would impart a directional shift in our estimates. Instead, consistency across many species indicates that broad trends in nesting phenology across spatial gradients and under variable weather conditions are robust. Future efforts that incorporate information on nest survival and land use or management practices would further strengthen predictive estimates and ecological insights for grassland‐nesting birds.

The nesting‐season predictions can serve as a valuable resource for navigating inherent tradeoffs in scheduling grassland management activities, particularly in finding optimal periods that accommodate necessary management interventions while minimizing impacts on active nests. In addition to supporting grassland birds, conservation lands are managed for multiple goals, including soil and water conservation, other wildlife species, pollinators, and biodiversity (Zhao et al. 2020). Maintaining grassland ecosystem services can require periodic disturbances such as grazing, mowing, and burning with prescribed fires. The timing, duration, and intensity of these actions vary widely, affecting other annual functions including the availability of suitable habitat for nesting birds. By using species‐ and guild‐specific predictions of nest timing, land managers can more accurately assess windows of vulnerability and schedule activities or develop recommendations for timing of management activities on agricultural lands or pastures as part of a conservation program to benefit nesting grassland birds. For example, most nests (5th percentile of initiation) are predicted to be initiated after 7 May in Kidder County, North Dakota and 95% of nests will likely be completed by 30 July when spring weather conditions are 1.5°C warmer than the long‐term average. A manager scheduling haying operations could use these estimates to avoid disturbance during this locally predicted nesting period rather than relying on state‐wide and fixed calendar guidelines. Further, managers could use the quantile‐based estimates to evaluate the proportion of nests likely to be active at a given time. Scheduling management or land use activities after the 90th percentile date would imply that approximately 10% of nests remain active, whereas waiting until after the 95th percentile would reduce nests at risk by roughly 50%. This framework makes the trade‐off between timing of management activities and biological impact more explicit. For instance, in Riley County, Kansas, the interval between the 5th and 10th percentile predicted initiation dates spans 5 days whereas the difference between the 90th and 95th percentile end dates is 11 days. The predicted timing differences illustrate how adjustment in timing of management activities could influence risks to active nests at either end of the nesting season.

Our study provides a multispecies evaluation of nesting phenology in grassland‐nesting birds across the midcontinental region of North America and reveals both long‐term shifts and interannual variability based on seasonal conditions. Our results indicate that the tails of the nesting window are expanding across this community of grassland birds, which may have implications for reproductive success, renesting opportunities, and synchrony with food availability, all of which may influence population trajectories. Importantly, this work provides a predictive framework that land managers can use to anticipate periods of nesting activity and adjust the timing of grassland management practices to minimize disturbances to nesting birds. However, estimates of nest timing produced from our study do not incorporate the post‐fledging period, where young may have limited mobility and may still be vulnerable to management actions (Berkeley et al. 2007; Hovick et al. 2011; Kershner et al. 2004; Suedkamp Wells et al. 2007; Wolcott et al. 2023; Yackel Adams et al. 2001). Further, although our models generate spatially continuous predictions, estimated nest initiation dates beyond the geographic and environmental range of our study data should be interpreted cautiously. For example, predictions in shrub‐steppe systems used by sagebrush specialists may be less reliable where breeding elevation, vegetation structures, and disturbance regimes differ from the midcontinent grassland systems evaluated in our analysis. As climate variability continues to alter environmental cues that birds rely on for breeding, incorporating spatially explicit predictions of nesting phenology into conservation planning may aid in the alignment of management actions and long‐term ecological requirements of grassland birds. Future work that links phenological patterns to fitness outcomes and integrates land use and management actions could help further our understanding of the consequences of altered nest timing for grassland‐nesting birds.

## Author Contributions

**Kristen S. Ellis:** data curation, writing – review and editing, investigation. **Michael J. Anteau:** conceptualization, writing – review and editing, funding acquisition. **Lawrence D. Igl:** conceptualization, writing – review and editing. **Garrett J. MacDonald:** conceptualization, writing – review and editing. **Neal D. Niemuth:** conceptualization, writing – review and editing. **Aaron T. Pearse:** conceptualization, writing – review and editing, funding acquisition. **Jill A. Shaffer:** conceptualization, writing – review and editing. **Christine A. Ribic:** conceptualization, writing – review and editing, investigation, data curation. **Josh L. Vest:** conceptualization, writing – review and editing, funding acquisition. **Masanori Abe:** writing – review and editing, data curation, investigation. **Daniel S. Ackerman:** writing – review and editing, data curation, investigation. **Marissa A. Ahlering:** writing – review and editing, data curation, investigation. **Johanna N. Alpert:** writing – review and editing, data curation, investigation. **Anahit Amiri:** data curation, writing – review and editing, investigation. **Kent Andersson:** writing – review and editing, data curation, investigation. **Jacqueline K. Augustine:** writing – review and editing, data curation, investigation. **Kristel K. Bakker:** writing – review and editing, data curation, investigation. **Jeffrey L. Beck:** writing – review and editing, data curation, investigation. **Adam C. Behney:** writing – review and editing, data curation, investigation. **Jacy S. Bernath‐Plaisted:** writing – review and editing, data curation, investigation. **Todd R. Bogenschutz:** data curation, writing – review and editing, investigation. **Eric K. Bollinger:** writing – review and editing, data curation, investigation. **Lauren E. Bortolotti:** writing – review and editing, data curation, investigation. **Andy J. Boyce:** investigation, writing – review and editing, data curation. **Jeffrey D. Brawn:** writing – review and editing, data curation, investigation. **Fred C. Bryant:** data curation, writing – review and editing, investigation. **Andrew J. Campomizzi:** writing – review and editing, data curation, investigation. **Jay D. Carlisle:** data curation, writing – review and editing, investigation. **J. Matthew Carroll:** writing – review and editing, data curation, investigation. **Rachel Carroll:** data curation, writing – review and editing, investigation. **John P. Carroll:** writing – review and editing, data curation, investigation. **Justin R. Clarke:** data curation, writing – review and editing, investigation. **Michael R. Conover:** writing – review and editing, data curation, investigation. **Reesa Y. Conrey:** data curation, writing – review and editing, investigation. **Jaime J. Coon:** writing – review and editing, data curation, investigation. **Kyle A. Cutting:** writing – review and editing, data curation, investigation. **Stephen K. Davis:** data curation, writing – review and editing, investigation. **Dawn M. Davis:** writing – review and editing, data curation, investigation. **Duane R. Diefenbach:** data curation, writing – review and editing, investigation. **Jonathan B. Dinkins:** writing – review and editing, data curation, investigation. **R. Andrew Dreelin:** data curation, writing – review and editing, investigation. **Victoria J. Dreitz:** writing – review and editing, data curation, investigation. **Courtney J. Duchardt:** data curation, writing – review and editing, investigation. **Michael W. Eichholz:** writing – review and editing, data curation, investigation. **Susan N. Ellis‐Felege:** writing – review and editing, data curation, investigation. **Kevin S. Ellison:** writing – review and editing, data curation, investigation. **Bradley C. Fedy:** writing – review and editing, data curation, investigation. **Samantha R. Fino:** writing – review and editing, data curation, investigation. **Robert J. Fletcher Jr.:** investigation, writing – review and editing, data curation. **Dillon T. Fogarty:** writing – review and editing, data curation, investigation. **Samuel D. Fuhlendorf:** data curation, writing – review and editing, investigation. **Pamela R. Garrettson:** writing – review and editing, data curation, investigation. **Robert J. Gates:** writing – review and editing, data curation, investigation. **Erin L. Gelling:** data curation, writing – review and editing, investigation. **James J. Giocomo:** writing – review and editing, data curation, investigation. **Alex Glass:** data curation, writing – review and editing, investigation. **Cheri L. Gratto‐Trevor:** writing – review and editing, data curation, investigation. **Kirsty E. B. Gurney:** writing – review and editing, data curation, investigation. **Ryan D. Haffele:** data curation, writing – review and editing, investigation. **Christian A. Hagen:** writing – review and editing, data curation, investigation. **Vanessa B. Harriman:** data curation, writing – review and editing, investigation. **Samuel W. H. Harryman:** writing – review and editing, data curation, investigation. **David A. Haukos:** data curation, writing – review and editing, investigation. **Robert T. Haynam:** writing – review and editing, data curation, investigation. **Heather A. Herakovich:** data curation, writing – review and editing, investigation. **James R. Herkert:** writing – review and editing, data curation, investigation. **Fidel Hernández:** data curation, writing – review and editing, investigation. **Jason M. Hill:** writing – review and editing, data curation, investigation. **H. Jeffrey Homan:** data curation, writing – review and editing, investigation. **Torre J. Hovick:** writing – review and editing, data curation, investigation. **Jonathan A. Jenks:** data curation, writing – review and editing, investigation. **William E. Jensen:** writing – review and editing, data curation, investigation. **Aaron N. Johnston:** data curation, writing – review and editing, investigation. **Sydney‐Marie Jones:** writing – review and editing, data curation, investigation. **Carly S. Kelly:** data curation, writing – review and editing, investigation. **Kaylan M. Kemink:** writing – review and editing, data curation, investigation. **Silka L. F. Kempema:** data curation, writing – review and editing, investigation. **Dylan C. Kesler:** writing – review and editing, data curation, investigation. **Christopher P. Kirol:** writing – review and editing, data curation, investigation. **Robert W. Klaver:** data curation, writing – review and editing, investigation. **Page E. Klug:** writing – review and editing, data curation, investigation. **Jesse L. Kolar:** data curation, writing – review and editing, investigation. **Nicola Koper:** writing – review and editing, data curation, investigation. **Kyle J. Kuechle:** data curation, writing – review and editing, investigation. **Jonathan D. Lautenbach:** writing – review and editing, data curation, investigation. **Zoe M. Lebrun‐Southcott:** data curation, writing – review and editing, investigation. **David W. Londe:** writing – review and editing, data curation, investigation. **Scott R. Loss:** writing – review and editing, data curation, investigation. **Cullen M. Mackenzie:** data curation, writing – review and editing, investigation. **Patrick A. Magee:** writing – review and editing, data curation, investigation. **Douglas L. Manzer:** writing – review and editing, data curation, investigation. **John P. McCarty:** data curation, writing – review and editing, investigation. **Gwyn McKee:** writing – review and editing, data curation, investigation. **Ann E. McKellar:** data curation, writing – review and editing, investigation. **Lance B. McNew:** writing – review and editing, data curation, investigation. **James R. Miller:** data curation, writing – review and editing, investigation. **Megan C. Milligan:** writing – review and editing, data curation, investigation. **Ashlee K. Minor:** data curation, writing – review and editing, investigation. **Rocco J. Murano:** writing – review and editing, data curation, investigation. **Les D. Murray:** data curation, writing – review and editing, investigation. **David E. Naugle:** writing – review and editing, data curation, investigation. **Clayton K. Nielsen:** data curation, writing – review and editing, investigation. **Christopher J. Norment:** writing – review and editing, data curation, investigation. **Sarah B. Ogden:** data curation, writing – review and editing, investigation. **Lindsey A. Parsons:** writing – review and editing, data curation, investigation. **Brian D. Peer:** data curation, writing – review and editing, investigation. **Noah G. Perlut:** writing – review and editing, data curation, investigation. **James C. Pitman:** writing – review and editing, data curation, investigation. **Christopher D. Pollentier:** data curation, writing – review and editing, investigation. **Larkin A. Powell:** writing – review and editing, data curation, investigation. **Aaron C. Pratt:** data curation, writing – review and editing, investigation. **John P. Pulliam:** writing – review and editing, data curation, investigation. **Nancy M. Raginski:** data curation, writing – review and editing, investigation. **Dan L. Reinking:** writing – review and editing, data curation, investigation. **Kaitlyn M. Reintsma:** data curation, writing – review and editing, investigation. **Terry Z. Riley:** writing – review and editing, data curation, investigation. **Kevin M. Ringelman:** data curation, writing – review and editing, investigation. **Joseph R. Robb:** investigation, writing – review and editing, data curation. **Scott K. Robinson:** data curation, writing – review and editing, investigation. **Andres N. Rosales:** writing – review and editing, data curation, investigation. **Charlotte L. Roy:** data curation, writing – review and editing, investigation. **Kayla A. Ruth:** writing – review and editing, data curation, investigation. **Brett K. Sandercock:** data curation, writing – review and editing, investigation. **Kevin K. Sedivec:** investigation, writing – review and editing, data curation. **Steve K. Sherrod:** data curation, writing – review and editing, investigation. **Justin J. Shew:** writing – review and editing, data curation, investigation. **Mason L. Sieges:** data curation, writing – review and editing, investigation. **Jenny L. Sika:** writing – review and editing, data curation, investigation. **Susan K. Skagen:** data curation, writing – review and editing, investigation. **Cassandra G. Skaggs:** writing – review and editing, data curation, investigation. **Joseph Smith:** data curation, writing – review and editing, investigation. **Jennifer A. Smith:** writing – review and editing, data curation, investigation. **Kurt T. Smith:** data curation, writing – review and editing, investigation. **Alex J. Solem:** writing – review and editing, data curation, investigation. **Joshua D. Stafford:** data curation, writing – review and editing, investigation. **Glenn E. Stauffer:** writing – review and editing, data curation, investigation. **Matthew D. Stephenson:** data curation, writing – review and editing, investigation. **Lee Sutcliffe:** writing – review and editing, data curation, investigation. **Amber E. Swicegood:** data curation, writing – review and editing, investigation. **Jason Tack:** writing – review and editing, data curation, investigation. **Evan P. Tanner:** data curation, writing – review and editing, investigation. **Catrina V. Terry:** writing – review and editing, data curation, investigation. **Wendy P. Tori:** data curation, writing – review and editing, investigation. **Ashley M. Unger:** writing – review and editing, data curation, investigation. **Kelly R. VanBeek:** data curation, writing – review and editing, investigation. **William E. Vetter:** writing – review and editing, data curation, investigation. **Jay A. VonBank:** investigation, writing – review and editing, data curation. **Brett L. Walker:** writing – review and editing, data curation, investigation. **Michael D. Whitson:** writing – review and editing, data curation, investigation. **Maiken Winter:** data curation, writing – review and editing, investigation. **Kimberly A. With:** writing – review and editing, data curation, investigation. **Donald H. Wolfe:** data curation, writing – review and editing, investigation. **Amy A. Yackel Adams:** writing – review and editing, data curation, investigation. **Benjamin Zuckerberg:** data curation, writing – review and editing, investigation.

## Funding

Funding for this study was provided by the U.S. Fish and Wildlife Service (Mountain Prairie Region: Migratory Birds/Science Applications, Refuges, Partners for Fish and Wildlife Program) and U.S. Department of Agriculture's Farm Service Agency. In‐kind internal support was provided by the U.S. Geological Survey's Northern Prairie Wildlife Research Center, U.S. Fish and Wildlife Service's Habitat and Population Evaluation Team, and the Prairie Pothole Joint Venture. Funding of data collection from individual studies is available by contacting authors directly. We gratefully acknowledge the numerous agencies, organizations, and institutions that supported the collection of data used in this study (see Supporting Information). External funders were not involved in decisions about study design, data collection, analysis and interpretation, or publication.

## Ethics Statement

We synthesized data from hundreds of independent studies conducted by numerous researchers, institutions, and agencies, each conducted under appropriate permits and approvals. Given the scope of this compilation, we acknowledge the original investigators and their supporting organizations for these contributions. Study‐specific information on animal care and use compliance, permissions, and permits can be requested by contacting the authors directly.

## Conflicts of Interest

The authors declare no conflicts of interest.

## Supporting information


**Figure S1:** Number of nests in each taxonomic group from 1969 to 2023 (left column), and site‐ or nest‐level location data in each group across all years (right column). States and provinces containing data from each group are shown in gray.
**Figure S2:** Species‐specific estimates of skew in nest initiation dates where positive values indicate the distribution is right‐skewed (more early nesting dates) and negative values indicate the distribution is left‐skewed (more late nesting dates). Points show median estimates with error bars showing 66% (thick bars) and 90% (thin bars) credible intervals. Species are listed in taxonomic order and organized by taxonomic groups (Chesser et al. 2025).
**Figure S3:** Species‐specific model validation results for seven quantile regression models. Each panel corresponds to a modeled quantile, with the proportion of observed nest initiation dates falling below the predicted quantile. Perfect calibration would result in values aligning with the expected quantile (red dashed line), with deviations indicating over‐ or underestimation of nest initiation timing for individual species. Species are listed in taxonomic order and organized by taxonomic groups (Chesser et al. 2025).
**Table S1:** Geographic and temporal coverage for each species included in the nesting phenology analysis. Columns include species common and scientific names, total nest count, summary of years in which data were collected (range, mean, and standard deviation), and two‐letter abbreviations of US states and Canadian provinces where data were collected. Species are listed in taxonomic order and organized by taxonomic groups (Chesser et al. 2025).
**Table S2:** Summary of predictor variables used in quantile regression models for nest initiation dates including temperature anomaly (°C, deviation from long‐term average), precipitation anomaly (mm; deviation from long‐term average), year, mid green‐up dates (day of year; January 1 = day 1), latitude, and longitude.
**Table S3:** Posterior estimates (mean and 90% credible intervals) of standardized global model parameters across all species for seven quantile regression models. The intercept represents average nest initiation dates (day of year; January 1 = day 1) at varying percentiles and estimates for year represent days per decade.
**Table S4:** Posterior estimates (mean and 90% credible intervals) of standardized global model parameters across non‐waterfowl species for three quantile regression models. The intercept represents average nest initiation dates (day of year; January 1 = day 1) at varying percentiles and estimates for year represent days per decade.
**Table S5:** Posterior estimates (mean and 90% credible intervals) of standardized species‐specific intercepts (day of year) and slopes associated with effects of year (days per decade), latitude, longitude, mid green‐up dates, precipitation, and temperature for seven quantile regression models (*τ* = 0.05, 0.10, 0.25, 0.50, 0.75, 0.90, and 0.95). Because quantile‐specific parameters were estimated independently, a small number of species exhibited minor deviations from strictly ordered intercepts across quantiles (e.g., higher quantiles occurring slightly earlier than adjacent lower quantiles); these were rare and did not affect interpretation, and predicted nesting dates in the covariate space retained the expected monotonic ordering across quantiles. Species are arranged alphabetically by common names. Species with less than 200 nest records contributed to global responses, but species‐specific slopes were not estimated for American Bittern, Gray Partridge, Short‐eared Owl, Sedge Wren, LeConte's Sparrow, Northern Harrier, and Marbled Godwit. Species are listed in taxonomic order (Chesser et al. 2025).

## Data Availability

The dataset analyzed for this study can be found at U.S. Geological Survey (2026), Grassland bird nesting phenology for the midcontinent of North America: U.S. Geological Survey Data Release, https://doi.org/10.5066/P1HZGNNQ.
