# Supplementary material for: Phenological Flexibility in Nesting Grassland Birds Over a Half Century Informs Timing of Management
Source: Glob Chang Biol. 2026 Oct 12;32(10):e71128. doi: 10.1111/gcb.71128 (PMC13655638; doi:10.1111/gcb.71128)
Supplement: Supplementary file 1 [file GCB-32-e71128-s001.docx]

**Supplementary materials for ‘Phenological flexibility in nesting grassland birds over a half century informs timing of management’**

*Corresponding author: kellis@usgs.gov

1. Compiling nest initiation dates

Non-waterfowl nesting data

Our goal was to assemble nest initiation dates of grassland bird species in North America. To do this, K. Ellis, M. Anteau, L. Igl, G. MacDonald, N. Niemuth, A. Pearse, J. Shaffer, C. Ribic, and J. Vest contacted researchers and biologists across the grassland-nesting bird research community to request data from individual studies. We offered co-authorship on the resulting publication to collaborators that were willing to share their data. Our initial round of researcher contacts stemmed from a list of corresponding authors from studies identified in a literature review of grassland bird nesting phenology in North America (Anteau et al., 2023). We then identified species and regional data gaps and pursued an expansive list of authors that had published nesting studies on species that were included in our list of focal grassland bird species (Table S1). We identified biologists from federal, state, and non-profit organizations that have been or are currently involved in grassland bird research and management. This overall effort was primarily accomplished through email correspondence but also included working group and conference presentations. Information that we requested from collaborators included species names, nest initiation dates (day first egg was laid), year, and approximate or site-level GPS coordinates (Figure S1). We did not request that collaborators send nest-specific GPS coordinates out of consideration for time constraints, threatened or endangered species, and private landowners, although some did. We additionally identified published datasets that included nest initiation dates for our focal species and we incorporated those into our analysis (Boyle, 2023; Buhl & Koford, 2023; Burr et al., 2017; DeVries et al., 2010; Fidino, 2022; Jensen & Sandercock, 2020; Ribic & Herkert, 2023; Rumble et al., 2011; Sinnott et al., 2023; Weigand, 1980).

Waterfowl nesting data

For waterfowl nest initiation dates, we primarily focused on combining data from the U.S. and Canadian portions of the Prairie Pothole Region (Figure S1). U.S. data came from a long-term nesting database that is managed by USGS Northern Prairie Wildlife Research Center (Austin et al., 2017; Klett et al., 1988). This database originated as an effort to store nesting information from numerous field studies across time into a central database using a standardized format. Waterfowl nesting data from Canada were collected and managed by Ducks Unlimited Canada. Similar to the non-waterfowl data, we extracted and used species names, nest initiation dates, year, and site- or nest-level GPS coordinates as pieces of information for our analysis.

2. Model validation

To assess the accuracy of quantile regression models and the reliability of predictions, we conducted a suite of model validation steps. We performed posterior predictive checks to evaluate how well the model replicated quantiles of the observed data. We extracted posterior samples of the linear predictor and scale parameter and simulated nest initiation dates from the asymmetric Laplace distribution. We simulated posterior predictive distributions of each observation across posterior draws and compared the resulting predicted initiation dates to observed data (Figure S3). We evaluated model fit visually and by calculating the proportion of observed values falling below the predicted quantile. For a well-calibrated model, the observed coverage should match the quantile level.

3. Stan model code

This model was implemented in Stan (Carpenter et al., 2017) using custom functions for the asymmetric Laplace distribution likelihood (Yu & Moyeed, 2001) to estimate conditional quantiles. The data inputs included *N* number of nest records, *N_species* is the number of unique species, and *N_species_slopes* is the number of unique species for which species-specific slopes were estimated. Each observation included covariates for year, temperature, precipitation, green-up dates, latitude, and longitude, along with the response variable, *init* (nest initiation dates). Two indexing variables were used: *species*, which maps each observation to a species-level intercept, and *species_est*, which maps observations to species eligible for random slopes (with 0 indicating shared fixed effects only). The quantile of interest was set with *tau* and the overall intercept prior mean was defined as *MB0* using observed quantiles in the data.

functions {

real rho_quantile(real y, real quantile) {

if (y < 0) {

return y * (quantile - 1);

} else {

return y * quantile;

}

}

real asym_laplace_lpdf(real y, real mu, real sigma, real quantile) {

return log(quantile * (1 - quantile)) -

log(sigma) -

rho_quantile((y - mu) / sigma, quantile);

}

real asym_laplace_lcdf(real y, real mu, real sigma, real quantile) {

if (y < mu) {

return log(quantile) + (1 - quantile) * (y - mu) / sigma;

} else {

return log1m((1 - quantile) * exp(-quantile * (y - mu) / sigma));

}

}

real asym_laplace_lccdf(real y, real mu, real sigma, real quantile) {

if (y < mu) {

return log1m(quantile * exp((1 - quantile) * (y - mu) / sigma));

} else {

return log1m(quantile) - quantile * (y - mu) / sigma;

}

}

}

data {

int<lower=1> N;

int<lower=1> N_species;

int<lower=1> species[N];

int<lower=1> N_species_slopes;

int<lower=0, upper=N_species_slopes> species_est[N];

vector[N] year;

vector[N] temp;

vector[N] ppt;

vector[N] greenup;

vector[N] lat;

vector[N] lon;

vector[N] init; // Response variable (nest initiation date)

real<lower=0, upper=1> tau; // Quantile of interest

int<lower=1> MB0; //Observed quantile date for intercept prior

}

parameters {

// Fixed effects

real beta0;

real b_year;

real b_temp;

real b_ppt;

real b_greenup;

real b_lat;

real b_lon;

// Quantile scale

real<lower=0.001> sigma;

// Species-level random effect deviations

vector[N_species] z_sp;

vector[N_species_slopes] z_year;

vector[N_species_slopes] z_temp;

vector[N_species_slopes] z_ppt;

vector[N_species_slopes] z_greenup;

vector[N_species_slopes] z_lat;

vector[N_species_slopes] z_lon;

// Hierarchical SDs

real<lower=0> sigma_sp;

real<lower=0> sigma_year;

real<lower=0> sigma_temp;

real<lower=0> sigma_ppt;

real<lower=0> sigma_greenup;

real<lower=0> sigma_lon;

real<lower=0> sigma_lat;

}

transformed parameters {

vector[N_species] eta_sp = beta0 + sigma_sp * z_sp;

vector[N_species_slopes] eta_year_sp = sigma_year * z_year;

vector[N_species_slopes] eta_temp_sp = sigma_temp * z_temp;

vector[N_species_slopes] eta_ppt_sp = sigma_ppt * z_ppt;

vector[N_species_slopes] eta_greenup_sp = sigma_greenup * z_greenup;

vector[N_species_slopes] eta_lat_sp = sigma_lat * z_lat;

vector[N_species_slopes] eta_lon_sp = sigma_lon * z_lon;

}

model {

//Priors

beta0 ~ normal(MB0, 15);

b_year ~ normal(0, 10);

b_temp ~ normal(0, 10);

b_ppt ~ normal(0, 10);

b_greenup ~ normal(0, 10);

b_lat ~ normal(0, 10);

b_lon ~ normal(0, 10);

sigma ~ gamma(1, 0.1);

sigma_sp ~ gamma(0.1, 0.1);

sigma_year ~ gamma(1, 0.1);

sigma_temp ~ gamma(1, 0.1);

sigma_ppt ~ gamma(1, 0.1);

sigma_greenup ~ gamma(1, 0.1);

sigma_lat ~ gamma(1, 0.1);

sigma_lon ~ gamma(1, 0.1);

z_sp ~ std_normal();

z_year ~ std_normal();

z_temp ~ std_normal();

z_ppt ~ std_normal();

z_greenup ~ std_normal();

z_lat ~ std_normal();

z_lon ~ std_normal();

for (i in 1:N) {

real slope_year = b_year;

real slope_temp = b_temp;

real slope_ppt = b_ppt;

real slope_greenup = b_greenup;

real slope_lat = b_lat;

real slope_lon = b_lon;

if (species_est[i] > 0){

slope_year += eta_year_sp[species_est[i]];

slope_temp += eta_temp_sp[species_est[i]];

slope_ppt += eta_ppt_sp[species_est[i]];

slope_greenup += eta_greenup_sp[species_est[i]];

slope_lat += eta_lat_sp[species_est[i]];

slope_lon += eta_lon_sp[species_est[i]];

}

real mu = eta_sp[species[i]] +

slope_year * year[i] +

slope_temp * temp[i] +

slope_ppt * ppt[i] +

slope_greenup * greenup[i] +

slope_lat * lat[i] +

slope_lon * lon[i];

init[i] ~ asym_laplace(mu, sigma, tau);

}

}

4. Additional funding acknowledgments

Additional support and funding for data collection was provided by Environment and Climate Change Canada and Alberta North America Waterfowl Management Plan Biodiversity Fund with logistic support from Ducks Unlimited Canada (Alberta) and Eastern Irrigation District Alberta (C. Gratto-Trevor); USDA CSREES-NRI Managed Ecosystems Program Grant #2003-35101-13714 (K. With); Earlham College through Matthews Student/Faculty Research in Physics/Biological Science, Jim Fowler Wildlife Conservation Fund, James B. Cope Endowed Student-Faculty Vertebrate Zoology Field Research Fund and Alphaeus Test Research Fund (W. Tori); Bureau of Land Management, Wyoming Game & Fish Department, The Nature Conservancy, MPG Ranch, McDanel Land Foundation, Page Family Foundation, Meg and Bert Raynes Wildlife Fund, Wyoming Governor’s Big Game License Coalition, and US Fish and Wildlife Service (J. Carlisle); Peabody Powder River Mining LLC-North Antelope Rochelle Mine and USFS Thunder Basin National Grassland (G. McKee, W.E. Vetter, and D.W. Pellatz). The Grand River Grasslands project was supported by Iowa State Wildlife Competitive Grants Program in Cooperation with the U.S. Fish and Wildlife Service (State Wildlife Grant SWG-C #14CRDWBKReed-0011); the U.S. Department of Agriculture National Institute of Food and Agriculture (grant 2016-67019-25206 and Hatch projects ILLU-875-918 and 1026535), a graduate student grant from Sustainable Agriculture, Research, and Education, North-Central Region (grant GNC15-201); the Frances M. Peacock Scholarship for Native Bird Habitat; the Louis Aggasiz Fuertes Award from the Wilson Ornithological Society; and multiple Undergraduate Research Fellowships from the College of Agricultural, Consumer, and Environmental Sciences.

Figure S1. Number of nests in each taxonomic group from 1969 to 2023 (left column), and site- or nest-level location data in each group across all years (right column). States and provinces containing data from each group are shown in grey.

Figure S2. Species-specific estimates of skew in nest initiation dates where positive values indicate the distribution is right-skewed (more early nesting dates) and negative values indicate the distribution is left-skewed (more late nesting dates). Points show median estimates with error bars showing 66% (thick bars) and 90% (thin bars) credible intervals. Species are listed in taxonomic order and organized by taxonomic groups (Chesser et al., 2025).

Figure S3. Species-specific model validation results for seven quantile regression models. Each panel corresponds to a modeled quantile, with the proportion of observed nest initiation dates falling below the predicted quantile. Perfect calibration would result in values aligning with the expected quantile (red dashed line), with deviations indicating over- or underestimation of nest initiation timing for individual species. Species are listed in taxonomic order and organized by taxonomic groups (Chesser et al., 2025).

Table S1. Geographic and temporal coverage for each species included in the nesting phenology analysis. Columns include species common and scientific names, total nest count, summary of years in which data were collected (range, mean, and standard deviation), and two-letter abbreviations of U.S. states and Canadian provinces where data were collected. Species are listed in taxonomic order and organized by taxonomic groups (Chesser et al., 2025).

|  | Common name | Scientific name | Sample size | Study years | States and provinces |
| --- | --- | --- | --- | --- | --- |
| **Waterfowl** | | | | | |
|  | Blue-winged Teal | *Spatula discors* | 40,431 | 1969-2023 (1996, 13) | AB, MB, MN, MT, ND, SD, SK |
|  | Northern Shoveler | *Spatula clypeata* | 11,857 | 1969-2022 (1999, 11) | AB, MB, MN, MT, ND, SD, SK |
|  | Gadwall | *Mareca strepera* | 27,367 | 1969-2023 (1997, 11) | AB, MB, MN, MT, ND, SD, SK |
|  | American Wigeon | *Mareca americana* | 1,656 | 1969-2023 (1990, 10) | AB, MB, MN, MT, ND, SD, SK |
|  | Mallard | *Anas platyrhynchos* | 30,036 | 1969-2023 (1997, 11) | AB, IA, MB, MN, MT, ND, SD, SK |
|  | Northern Pintail | *Anas acuta* | 10,416 | 1969-2023 (1996, 13) | AB, MB, MN, MT, ND, SD, SK |
|  | Green-winged Teal | *Anas crecca* | 896 | 1969-2023 (1993, 11) | AB, MB, MN, MT, ND, SD, SK |
|  | Lesser Scaup | *Aythya affinis* | 2,502 | 1969-2023 (1995, 11) | AB, MB, MT, ND, SD, SK |
| **Upland gamebirds** | | | | | |
|  | Northern Bobwhite | *Colinus virginianus* | 934 | 1993-2023 (2011, 6) | CO, IA, IL, KS, MO, OH, OK, TX |
|  | Greater Sage-Grouse | *Centrocercus urophasianus* | 4,488 | 1992-2023 (2014, 6) | ID, MT, ND, SD, SK, WY |
|  | Sharp-tailed Grouse | *Tympanuchus phasianellus* | 1,271 | 1978-2023 (2007, 13) | AB, MT, ND, NE, SD, SK, WY |
|  | Greater Prairie-Chicken | *Tympanuchus cupido* | 1,113 | 1975-2023 (2011, 8) | KS, MN, MO, NE, OK, SD, WI |
|  | Lesser Prairie-Chicken | *Tympanuchus pallidicinctus* | 1,228 | 1977-2022 (2009, 8) | CO, KS, NM, OK, TX |
|  | Gray Partridge | *Perdix perdix* | 39 | 1969-2015 (1983, 9) | IA, MT, ND |
|  | Ring-necked Pheasant | *Phasianus colchicus* | 1,212 | 1978-2020 (2004, 10) | CO, IA, MN, SD |
| **Shorebirds and herons** | | | | | |
|  | Mountain Plover | *Anarhynchus montanus* | 359 | 2006-2017 (2012, 4) | CO, NE, WY |
|  | Upland Sandpiper | *Bartramia longicauda* | 819 | 1975-2023 (1997, 11) | AB, IA, IL, KS, MB, MN, ND, NE, OK, SD, SK, WI |
|  | Long-billed Curlew | *Numenius americanus* | 239 | 1997-2023 (2017, 5) | AB, ID, MT, ND, NE, NM, SD, SK, WY |
|  | Marbled Godwit | *Limosa fedoa* | 187 | 1982-2021 (1999, 7) | AB, MN, MT, ND, SD, SK |
|  | Wilson's Phalarope | *Phalaropus tricolor* | 268 | 1975-2023 (2000, 13) | ID, MT, ND, SD, SK |
|  | Willet | *Tringa semipalmata* | 420 | 1981-2023 (2000, 7) | AB, KS, MB, MT, ND, NE, SD, SK |
|  | American Bittern | *Botaurus lentiginosus* | 32 | 1976-2023 (1996, 15) | ND, NE, SD, SK |
| **Raptors and owls** | | | | | |
|  | Northern Harrier | *Circus hudsonius* | 143 | 1977-2012 (1994, 11) | AB, IA, ID, MN, ND, OK, SD, UT |
|  | Short-eared Owl | *Asio flammeus* | 58 | 1978-2011 (1996, 11) | AB, ID, ND, SD, UT |
| **Passerines** | | | | | |
|  | Horned Lark | *Eremophila alpestris* | 425 | 1991-2023 (2009, 12) | AB, CO, IL, KS, MT, ND, NE, SK, TX, WI |
|  | Sedge Wren | *Cistothorus stellaris* | 67 | 1993-2022 (2004, 7) | IA, IL, MN, ND, SD, WI |
|  | Sprague's Pipit | *Anthus spragueii* | 712 | 1996-2023 (2006, 6) | AB, MT, ND, SK |
|  | Chestnut-collared Longspur | *Calcarius ornatus* | 2,858 | 1992-2023 (2009, 9) | AB, MB, MT, ND, SD, SK |
|  | Thick-billed Longspur | *Rhynchophanes mccownii* | 529 | 2012-2021 (2018, 3) | CO, MT, SK |
|  | Grasshopper Sparrow | *Ammodramus savannarum* | 2,921 | 1991-2023 (2006, 8) | CO, IA, IL, KS, KY, MN, MT, ND, NE, OK, ON, PA, SD, SK, TX, VT, WI |
|  | Clay-colored Sparrow | *Spizella pallida* | 3,119 | 1992-2023 (2005, 8) | MB, MN, ND, SD, SK, WI |
|  | Field Sparrow | *Spizella pusilla* | 1,678 | 1989-2023 (2004, 8) | IA, IL, IN, KS, KY, MO, ND, OK, PA, WI |
|  | Brewer's Sparrow | *Spizella breweri* | 956 | 2003-2019 (2012, 6) | CO, MT, SK, WY |
|  | Lark Sparrow | *Chondestes grammacus* | 592 | 1992-2018 (2003, 5) | CO, IA, IL, KS, ND, NE, OK, TX, WY |
|  | Lark Bunting | *Calamospiza melanocorys* | 1,023 | 1991-2021 (2002, 5) | CO, KS, MT, ND, NE, SK, WY |
|  | Vesper Sparrow | *Pooecetes gramineus* | 1,744 | 1991-2019 (2007, 8) | AB, CO, IA, IL, KS, MB, MN, MT, ND, NE, PA, SK, WI, WY |
|  | LeConte's Sparrow | *Ammospiza leconteii* | 98 | 1998-2012 (2002, 5) | MB, MN, ND |
|  | Savannah Sparrow | *Passerculus sandwichensis* | 5,524 | 1991-2023 (2006, 7) | AB, IA, IL, KS, MB, MN, MT, ND, NY, ON, PA, SD, SK, VT, WI |
|  | Baird's Sparrow | *Centronyx bairdii* | 649 | 1996-2022 (2006, 8) | AB, MT, ND, SK |
|  | Henslow's Sparrow | *Centronyx henslowii* | 414 | 1992-2022 (2003, 6) | IA, IL, IN, KS, KY, OK, PA, WI |
|  | Bobolink | *Dolichonyx oryzivorus* | 2,859 | 1992-2023 (2008, 8) | IA, IL, MN, ND, NY, ON, PA, SD, SK, VT, WI |
|  | Western Meadowlark | *Sturnella neglecta* | 1,162 | 1981-2023 (2001, 11) | AB, CO, IA, IL, MB, MN, MT, ND, NE, SD, SK, TX, WI |
|  | Eastern Meadowlark | *Sturnella magna* | 1,895 | 1992-2023 (2002, 7) | IA, IL, IN, KS, KY, NY, OK, ON, PA, VT, WI |
|  | Dickcissel | *Spiza americana* | 4,962 | 1991-2023 (2005, 8) | IA, IL, KS, KY, MN, ND, NE, OK, WI |

Table S2. Summary of predictor variables used in quantile regression models for nest initiation dates including temperature anomaly (°C, deviation from long-term average), precipitation anomaly (mm; deviation from long-term average), year, mid green-up dates (day of year; January 1 = day 1), latitude, and longitude.

| Predictor variable | Range | Mean (SD) |
| --- | --- | --- |
| Temperature | (-2.8, 3.3) | -0.09 (1.1) |
| Precipitation | (-44.4, 50.1) | 0.9 (10.0) |
| Year | (1969, 2023) | 1999 (12) |
| Mid green-up | (52.5, 189.0) | 149.0 (15.3) |
| Latitude | (26.9, 54.0) | 47.0 (3.6) |
| Longitude | (-117.0, -73.2) | -101.0 (6.1) |

Table S3. Posterior estimates (mean and 90% credible intervals) of standardized global model parameters across all species for seven quantile regression models. The intercept represents average nest initiation dates (day of year; January 1 = day 1) at varying percentiles and estimates for year represent days per decade.

| Parameter | Quantile model | | | | | | |
| --- | --- | --- | --- | --- | --- | --- | --- |
|  | 5% | 10% | 25% | 50% | 75% | 90% | 95% |
| Intercept | 129.87 (126.23, 133.17) | 133.66 (130.36, 136.84) | 140.89 (137.60, 144.36) | 151.22 (147.65, 154.93) | 163.94 (159.88, 167.86) | 174.97 (170.74, 179.43) | 180.01 (175.92, 184.51) |
| Temperature | -1.65 (-2.00, -1.33) | -1.55 (-1.90, -1.18) | -1.43 (-1.87, -0.99) | -1.51 (-2.05, -0.95) | -1.33 (-1.89, -0.76) | -1.18 (-1.73, -0.63) | -0.96 (-1.58, -0.32) |
| Precipitation | -0.48 (-0.79, -0.17) | -0.48 (-0.79, -0.18) | -0.54 (-0.86, -0.22) | -1.02 (-1.51, -0.56) | -0.90 (-1.55, -0.29) | -0.84 (-1.50, -0.21) | -0.68 (-1.32, -0.07) |
| Year | -1.64 (-2.50, -0.81) | -0.81 (-1.68, -0.03) | -0.57 (-1.25, 0.06) | 0.10 (-0.56, 0.75) | 0.65 (-0.23, 1.53) | 0.12 (-0.77, 1.02) | 0.62 (-0.31, 1.56) |
| Mid green-up | 0.96 (0.38, 1.62) | 1.16 (0.54, 1.88) | 0.95 (0.32, 1.61) | 0.84 (0.11, 1.56) | 0.07 (-1.00, 1.01) | -0.16 (-1.34, 0.89) | -0.01 (-1.28, 1.19) |
| Latitude | 5.95 (4.86, 7.07) | 4.95 (4.00, 5.97) | 3.96 (3.12, 4.80) | 3.36 (2.41, 4.29) | 1.88 (0.67, 3.13) | 0.89 (-0.49, 2.15) | -1.36 (-3.16, 0.37) |
| Longitude | 1.02 (0.34, 1.68) | 0.93 (0.19, 1.71) | 0.93 (0.04, 1.87) | 1.14 (0.08, 2.23) | -0.02 (-1.06, 1.03) | 0.09 (-0.86, 1.04) | -0.95 (-2.07, 0.19) |
| $\boldsymbol{\sigma}$ (scale parameter) | 1.26 (1.25, 1.26) | 2.24 (2.23, 2.25) | 4.41 (4.40, 4.43) | 6.02 (6.00, 6.05) | 4.96 (4.94, 4.98) | 2.72 (2.71, 2.73) | 1.57 (1.56, 1.57) |

Table S4. Posterior estimates (mean and 90% credible intervals) of standardized global model parameters across non-waterfowl species for three quantile regression models. The intercept represents average nest initiation dates (day of year; January 1 = day 1) at varying percentiles and estimates for year represent days per decade.

| Parameter | Quantile model | | |  |
| --- | --- | --- | --- | --- |
|  | 5% | 50% | 95% | |
| Intercept | 126.77 (122.69, 130.64) | 148.87 (144.65, 152.96) | 180.99 (175.29, 186.84) | |
| Temperature | -1.41 (-1.80, -1.01) | -1.28 (-1.84, -0.70) | -0.68 (-1.30, -0.03) | |
| Precipitation | -0.49 (-0.98, -0.01) | -1.47 (-2.14, -0.83) | -1.04 (-1.85, -0.22) | |
| Year | -2.01 (-3.01, -0.99) | 0.06 (-0.75, 0.85) | 0.63 (-0.50, 1.77) | |
| Mid green-up | 1.37 (0.59, 2.21) | 1.02 (0.01, 2.04) | -0.56 (-2.27, 1.02) | |
| Latitude | 7.93 (6.40, 9.56) | 3.94 (2.32, 5.51) | -3.17 (-5.73, -0.76) | |
| Longitude | 1.39 (0.08, 2.70) | 1.44 (-0.58, 3.48) | -2.87 (-4.69, -1.06) | |
| $\boldsymbol{\sigma}$ (scale parameter) | 1.31 (1.30, 1.32) | 6.79 (6.73, 6.84) | 1.81 (1.80, 1.82) | |

Table S5. Posterior estimates (mean and 90% credible intervals) of standardized species-specific intercepts (day of year) and slopes associated with effects of year (days per decade), latitude, longitude, mid green-up dates, precipitation, and temperature for seven quantile regression models (τ = 0.05, 0.10, 0.25, 0.50, 0.75, 0.90, and 0.95). Because quantile-specific parameters were estimated independently, a small number of species exhibited minor deviations from strictly ordered intercepts across quantiles (e.g., higher quantiles occurring slightly earlier than adjacent lower quantiles); these were rare and did not affect interpretation, and predicted nesting dates in the covariate space retained the expected monotonic ordering across quantiles. Species are arranged alphabetically by common names. Species with less than 200 nest records contributed to global responses, but species-specific slopes were not estimated for American Bittern, Gray Partridge, Short-eared Owl, Sedge Wren, LeConte’s Sparrow, Northern Harrier, and Marbled Godwit. Species are listed in taxonomic order (Chesser et al., 2025).

| **Species** | **Parameter** | **5%** | **10%** | **25%** | **50%** | **75%** | **90%** | **95%** |
| --- | --- | --- | --- | --- | --- | --- | --- | --- |
| Blue-winged Teal | Intercept | 126.92 (126.83, 127.01) | 141.31 (139.77, 142.80) | 150.30 (143.52, 156.89) | 153.08 (150.91, 155.32) | 144.18 (141.82, 146.31) | 162.67 (158.41, 166.75) | 183.18 (182.57, 183.83) |
| Blue-winged Teal | Year | -0.97 (-1.02, -0.91) | -0.84 (-0.91, -0.76) | -0.38 (-0.46, -0.31) | 0.06 (-0.03, 0.15) | 0.76 (0.65, 0.87) | 1.46 (1.36, 1.56) | 1.31 (1.22, 1.42) |
| Blue-winged Teal | Latitude | 3.75 (3.54, 3.96) | 3.94 (3.71, 4.17) | 4.39 (4.11, 4.67) | 4.98 (4.66, 5.30) | 5.88 (5.52, 6.23) | 5.91 (5.57, 6.24) | 5.13 (4.86, 5.41) |
| Blue-winged Teal | Longitude | -1.49 (-1.72, -1.26) | -1.49 (-1.75, -1.22) | -1.06 (-1.35, -0.77) | -0.69 (-1.03, -0.34) | 0.25 (-0.11, 0.60) | 0.67 (0.31, 1.02) | 1.25 (0.97, 1.54) |
| Blue-winged Teal | Mid green-up | -0.23 (-0.33, -0.13) | -0.08 (-0.20, 0.04) | 0.07 (-0.06, 0.21) | 0.24 (0.08, 0.40) | 0.56 (0.38, 0.75) | 0.62 (0.43, 0.80) | 0.86 (0.70, 1.02) |
| Blue-winged Teal | Precipitation | -0.31 (-0.39, -0.22) | -0.49 (-0.59, -0.39) | -0.46 (-0.56, -0.36) | -0.23 (-0.35, -0.11) | 0.70 (0.56, 0.83) | 1.29 (1.15, 1.43) | 1.65 (1.52, 1.78) |
| Blue-winged Teal | Temperature | -1.86 (-1.94, -1.77) | -1.75 (-1.84, -1.66) | -1.62 (-1.72, -1.53) | -1.75 (-1.86, -1.63) | -1.64 (-1.76, -1.51) | -1.62 (-1.76, -1.49) | -1.87 (-1.98, -1.75) |
| Northern Shoveler | Intercept | 142.88 (142.75, 143.02) | 173.52 (171.70, 175.29) | 104.69 (98.96, 109.93) | 141.90 (140.69, 143.11) | 190.30 (185.62, 195.29) | 203.67 (200.00, 207.56) | 145.63 (142.76, 148.86) |
| Northern Shoveler | Year | -0.62 (-0.77, -0.47) | -0.55 (-0.72, -0.38) | -0.28 (-0.46, -0.11) | 0.52 (0.32, 0.73) | 1.19 (1.00, 1.39) | 1.91 (1.69, 2.12) | 1.89 (1.68, 2.10) |
| Northern Shoveler | Latitude | 3.48 (2.96, 4.03) | 3.29 (2.86, 3.73) | 4.15 (3.67, 4.66) | 5.66 (5.07, 6.22) | 6.11 (5.48, 6.74) | 5.72 (5.10, 6.33) | 5.04 (4.48, 5.62) |
| Northern Shoveler | Longitude | 0.80 (0.37, 1.22) | 0.02 (-0.42, 0.46) | -0.08 (-0.53, 0.40) | 0.90 (0.38, 1.42) | 1.43 (0.90, 1.97) | 2.07 (1.49, 2.64) | 2.56 (2.04, 3.09) |
| Northern Shoveler | Mid green-up | 0.18 (-0.11, 0.46) | 0.11 (-0.15, 0.37) | 0.55 (0.25, 0.84) | 0.77 (0.44, 1.11) | 0.93 (0.59, 1.25) | 1.26 (0.89, 1.62) | 0.99 (0.64, 1.34) |
| Northern Shoveler | Precipitation | -0.71 (-0.91, -0.52) | -0.78 (-1.00, -0.56) | -0.79 (-1.01, -0.58) | -0.64 (-0.91, -0.39) | -0.01 (-0.28, 0.26) | 0.89 (0.61, 1.16) | 1.03 (0.78, 1.28) |
| Northern Shoveler | Temperature | -2.12 (-2.27, -1.96) | -2.26 (-2.43, -2.09) | -2.42 (-2.61, -2.24) | -2.68 (-2.90, -2.46) | -2.48 (-2.73, -2.25) | -2.03 (-2.27, -1.77) | -1.89 (-2.10, -1.70) |
| Gadwall | Intercept | 135.47 (135.35, 135.60) | 162.33 (160.61, 164.08) | 174.21 (168.07, 180.49) | 137.12 (135.94, 138.23) | 156.81 (153.35, 160.93) | 191.36 (186.56, 196.16) | 143.36 (140.59, 145.72) |
| Gadwall | Year | 0.27 (0.16, 0.38) | 0.14 (0.03, 0.24) | 0.21 (0.11, 0.31) | 0.18 (0.05, 0.32) | 0.43 (0.29, 0.57) | 0.48 (0.35, 0.60) | 0.30 (0.19, 0.41) |
| Gadwall | Latitude | 5.20 (4.91, 5.50) | 5.32 (5.01, 5.61) | 5.02 (4.69, 5.36) | 4.60 (4.21, 4.98) | 4.32 (3.87, 4.79) | 3.81 (3.36, 4.26) | 3.08 (2.74, 3.42) |
| Gadwall | Longitude | 1.80 (1.56, 2.07) | 2.29 (1.99, 2.62) | 1.87 (1.53, 2.20) | 1.65 (1.31, 1.98) | 2.74 (2.38, 3.11) | 3.68 (3.27, 4.09) | 3.59 (3.28, 3.90) |
| Gadwall | Mid green-up | -0.26 (-0.42, -0.09) | -0.23 (-0.41, -0.06) | -0.30 (-0.48, -0.13) | -0.21 (-0.43, 0.01) | -0.19 (-0.43, 0.04) | -0.48 (-0.69, -0.28) | -0.35 (-0.53, -0.17) |
| Gadwall | Precipitation | -0.63 (-0.75, -0.52) | -0.74 (-0.86, -0.61) | -0.72 (-0.85, -0.58) | -0.54 (-0.69, -0.40) | -0.19 (-0.36, -0.01) | 0.42 (0.28, 0.56) | 0.61 (0.48, 0.74) |
| Gadwall | Temperature | -1.70 (-1.81, -1.60) | -1.66 (-1.77, -1.56) | -1.92 (-2.02, -1.82) | -1.97 (-2.10, -1.84) | -2.03 (-2.17, -1.89) | -2.15 (-2.29, -2.01) | -1.97 (-2.11, -1.84) |
| American Wigeon | Intercept | 145.19 (145.05, 145.33) | 183.76 (175.66, 191.99) | 182.34 (177.59, 186.30) | 151.05 (148.34, 154.12) | 158.13 (156.11, 160.07) | 186.37 (179.91, 193.91) | 144.88 (144.18, 145.61) |
| American Wigeon | Year | -0.21 (-0.80, 0.38) | 0.97 (0.36, 1.57) | 1.71 (1.12, 2.29) | 1.72 (1.08, 2.35) | 2.06 (1.39, 2.70) | 1.90 (1.27, 2.55) | 1.03 (0.51, 1.56) |
| American Wigeon | Latitude | 4.25 (3.12, 5.41) | 4.13 (2.87, 5.36) | 3.86 (2.61, 5.15) | 3.86 (2.41, 5.37) | 5.60 (3.91, 7.30) | 5.53 (4.31, 6.78) | 6.39 (4.59, 7.93) |
| American Wigeon | Longitude | 0.81 (-0.26, 1.95) | 1.42 (0.34, 2.51) | 3.16 (2.01, 4.30) | 2.66 (1.45, 3.90) | 5.21 (3.82, 6.52) | 5.96 (4.97, 6.94) | 6.42 (5.16, 7.52) |
| American Wigeon | Mid green-up | 1.24 (0.56, 1.93) | 0.63 (-0.13, 1.40) | 0.52 (-0.27, 1.33) | 0.94 (0.05, 1.83) | 0.31 (-0.66, 1.29) | 1.24 (0.51, 1.93) | 0.46 (-0.32, 1.35) |
| American Wigeon | Precipitation | -1.65 (-2.40, -0.89) | -0.80 (-1.50, -0.13) | -0.09 (-0.82, 0.65) | 0.47 (-0.29, 1.25) | 1.05 (0.30, 1.81) | 1.18 (0.39, 2.00) | 1.26 (0.69, 1.88) |
| American Wigeon | Temperature | -1.99 (-2.48, -1.52) | -1.50 (-2.07, -0.96) | -1.97 (-2.49, -1.44) | -0.69 (-1.28, -0.09) | -0.88 (-1.46, -0.28) | -1.30 (-1.89, -0.74) | -1.62 (-2.12, -1.13) |
| Mallard | Intercept | 129.99 (129.87, 130.11) | 152.50 (151.04, 153.96) | 157.82 (151.65, 164.12) | 133.32 (131.77, 134.79) | 148.60 (146.29, 150.92) | 180.19 (175.65, 184.54) | 189.53 (188.92, 190.13) |
| Mallard | Year | -1.12 (-1.26, -0.98) | -1.25 (-1.39, -1.12) | -1.19 (-1.33, -1.05) | -0.16 (-0.31, 0.00) | 1.15 (1.01, 1.29) | 1.99 (1.86, 2.13) | 1.85 (1.72, 1.98) |
| Mallard | Latitude | 3.00 (2.65, 3.34) | 2.08 (1.66, 2.48) | 0.69 (0.26, 1.11) | 2.60 (2.13, 3.06) | 2.77 (2.30, 3.24) | 3.81 (3.41, 4.22) | 3.96 (3.56, 4.35) |
| Mallard | Longitude | 1.88 (1.53, 2.21) | 1.92 (1.51, 2.33) | 1.07 (0.65, 1.48) | 1.60 (1.16, 2.05) | 2.10 (1.63, 2.56) | 3.44 (3.02, 3.85) | 3.81 (3.43, 4.19) |
| Mallard | Mid green-up | -0.29 (-0.47, -0.10) | -0.22 (-0.42, -0.02) | 0.76 (0.55, 0.98) | 0.70 (0.44, 0.97) | 0.32 (0.10, 0.54) | -0.04 (-0.26, 0.17) | -0.35 (-0.56, -0.14) |
| Mallard | Precipitation | 0.33 (0.19, 0.47) | 0.26 (0.12, 0.41) | 0.44 (0.29, 0.60) | 0.68 (0.48, 0.87) | 0.94 (0.77, 1.12) | 1.45 (1.30, 1.61) | 1.92 (1.76, 2.07) |
| Mallard | Temperature | -1.15 (-1.25, -1.05) | -0.92 (-1.05, -0.80) | -0.94 (-1.08, -0.81) | -1.08 (-1.24, -0.92) | -1.39 (-1.53, -1.24) | -1.79 (-1.92, -1.66) | -1.92 (-2.06, -1.78) |
| Northern Pintail | Intercept | 152.77 (152.60, 152.93) | 178.30 (176.55, 180.08) | 118.43 (111.50, 124.84) | 149.43 (148.00, 150.88) | 203.41 (199.64, 207.09) | 148.63 (139.49, 158.20) | 151.57 (147.99, 155.15) |
| Northern Pintail | Year | -0.37 (-0.52, -0.21) | -0.26 (-0.43, -0.09) | -0.40 (-0.62, -0.18) | -0.81 (-1.06, -0.57) | -0.05 (-0.28, 0.17) | 0.77 (0.56, 0.97) | 0.97 (0.80, 1.15) |
| Northern Pintail | Latitude | 0.83 (0.34, 1.36) | 0.61 (0.09, 1.14) | -0.31 (-1.00, 0.38) | 0.72 (-0.23, 1.64) | 2.60 (1.69, 3.49) | 3.34 (2.49, 4.21) | 4.51 (3.79, 5.22) |
| Northern Pintail | Longitude | 1.41 (0.94, 1.92) | 2.07 (1.62, 2.52) | 3.25 (2.66, 3.85) | 3.84 (3.05, 4.65) | 3.77 (3.04, 4.47) | 3.85 (3.17, 4.52) | 4.40 (3.80, 5.00) |
| Northern Pintail | Mid green-up | 0.58 (0.32, 0.84) | 0.88 (0.60, 1.15) | 1.70 (1.33, 2.07) | 1.61 (1.18, 2.06) | 1.21 (0.76, 1.65) | 0.44 (0.05, 0.82) | 0.28 (-0.09, 0.63) |
| Northern Pintail | Precipitation | 0.86 (0.63, 1.07) | 0.54 (0.33, 0.75) | 0.80 (0.49, 1.11) | 1.24 (0.89, 1.60) | 1.41 (1.10, 1.73) | 1.46 (1.14, 1.79) | 1.73 (1.48, 1.97) |
| Northern Pintail | Temperature | -1.63 (-1.78, -1.49) | -1.71 (-1.88, -1.53) | -1.59 (-1.85, -1.33) | -2.38 (-2.68, -2.08) | -2.68 (-2.96, -2.41) | -2.71 (-2.99, -2.43) | -2.37 (-2.62, -2.13) |
| Green-winged Teal | Intercept | 162.26 (161.08, 163.47) | 139.42 (137.17, 141.71) | 137.05 (136.51, 137.57) | 141.97 (140.20, 143.90) | 175.57 (162.26, 188.77) | 130.48 (129.87, 131.09) | 141.80 (140.80, 142.75) |
| Green-winged Teal | Year | 1.60 (0.83, 2.29) | 1.37 (0.63, 2.04) | 0.88 (0.09, 1.66) | 1.07 (0.24, 1.91) | 0.73 (-0.21, 1.61) | 1.00 (0.23, 1.73) | 1.40 (0.61, 2.18) |
| Green-winged Teal | Latitude | 0.35 (-0.75, 1.46) | 0.13 (-1.44, 1.76) | 2.10 (0.12, 4.15) | 3.09 (0.95, 5.25) | 6.94 (4.65, 9.06) | 3.59 (1.57, 5.54) | 1.69 (-0.49, 3.94) |
| Green-winged Teal | Longitude | 1.96 (0.90, 2.98) | 2.24 (0.92, 3.62) | 3.31 (1.53, 5.15) | 4.22 (2.44, 5.99) | 7.01 (5.29, 8.60) | 4.50 (2.94, 5.98) | 3.65 (2.27, 5.06) |
| Green-winged Teal | Mid green-up | -0.26 (-1.34, 0.71) | 0.26 (-0.68, 1.17) | 0.18 (-0.99, 1.30) | -0.05 (-1.28, 1.22) | -0.56 (-1.83, 0.74) | -0.50 (-1.50, 0.57) | -0.54 (-1.78, 0.68) |
| Green-winged Teal | Precipitation | -0.56 (-1.21, 0.05) | -0.58 (-1.30, 0.15) | 0.28 (-0.64, 1.20) | 0.90 (-0.27, 2.05) | 2.01 (0.76, 3.27) | 1.13 (0.34, 1.95) | 0.99 (0.03, 1.92) |
| Green-winged Teal | Temperature | -0.74 (-1.32, -0.16) | -0.66 (-1.38, 0.05) | -0.36 (-1.17, 0.42) | -0.50 (-1.42, 0.37) | -0.59 (-1.55, 0.36) | -2.35 (-3.08, -1.59) | -2.46 (-3.20, -1.74) |
| Lesser Scaup | Intercept | 159.30 (158.96, 159.63) | 143.45 (138.12, 148.78) | 129.77 (126.88, 132.77) | 154.51 (150.96, 158.14) | 188.33 (183.97, 192.28) | 144.52 (141.70, 147.93) | 168.26 (159.06, 177.24) |
| Lesser Scaup | Year | 0.56 (0.02, 1.08) | 0.61 (0.12, 1.11) | 0.64 (0.23, 1.04) | 0.94 (0.54, 1.32) | 0.51 (0.09, 0.92) | 0.27 (-0.17, 0.69) | -0.22 (-0.63, 0.18) |
| Lesser Scaup | Latitude | 10.32 (9.39, 11.27) | 11.05 (9.99, 12.02) | 9.06 (8.08, 10.05) | 6.94 (5.84, 8.12) | 5.96 (4.80, 7.12) | 5.41 (4.25, 6.50) | 3.86 (2.37, 5.32) |
| Lesser Scaup | Longitude | 4.08 (3.28, 4.97) | 4.52 (3.57, 5.45) | 3.82 (2.82, 4.83) | 2.22 (1.23, 3.27) | 3.20 (2.22, 4.15) | 4.29 (3.37, 5.21) | 4.06 (2.74, 5.32) |
| Lesser Scaup | Mid green-up | -0.79 (-1.43, -0.13) | -0.61 (-1.25, 0.00) | -0.69 (-1.27, -0.11) | -0.94 (-1.62, -0.27) | -1.10 (-1.76, -0.43) | -0.49 (-1.23, 0.26) | 0.14 (-0.61, 0.90) |
| Lesser Scaup | Precipitation | -1.45 (-2.01, -0.90) | -2.12 (-2.57, -1.65) | -2.52 (-2.99, -2.04) | -2.21 (-2.70, -1.70) | -2.15 (-2.70, -1.58) | -2.29 (-2.84, -1.74) | -2.67 (-3.20, -2.14) |
| Lesser Scaup | Temperature | -0.76 (-1.25, -0.27) | -0.90 (-1.29, -0.48) | -0.36 (-0.77, 0.03) | -0.43 (-0.86, -0.01) | -0.61 (-1.09, -0.13) | -0.38 (-0.79, 0.03) | -0.86 (-1.34, -0.38) |
| Northern Bobwhite | Intercept | 152.18 (151.01, 153.38) | 136.90 (134.97, 138.82) | 134.72 (133.96, 135.45) | 135.13 (133.09, 137.01) | 167.67 (155.00, 180.56) | 190.44 (185.82, 196.45) | 132.23 (131.43, 133.05) |
| Northern Bobwhite | Year | 4.71 (2.90, 6.52) | 4.06 (2.46, 5.71) | 5.40 (3.39, 7.57) | 6.07 (3.22, 8.74) | 2.73 (-0.52, 5.93) | -5.28 (-7.93, -2.40) | -3.58 (-7.11, -0.17) |
| Northern Bobwhite | Latitude | 7.11 (6.31, 7.89) | 6.13 (5.34, 6.88) | 4.22 (3.25, 5.17) | 3.45 (2.15, 4.79) | -1.48 (-2.79, -0.13) | -1.44 (-2.46, -0.46) | -6.18 (-7.32, -4.98) |
| Northern Bobwhite | Longitude | -3.77 (-5.32, -2.27) | -3.48 (-4.52, -2.40) | -1.59 (-3.05, -0.14) | -0.85 (-2.87, 1.12) | 2.08 (-0.15, 4.21) | -0.63 (-2.27, 1.08) | -0.44 (-2.20, 1.29) |
| Northern Bobwhite | Mid green-up | 0.68 (-0.69, 2.20) | 1.48 (0.44, 2.46) | 1.98 (0.47, 3.48) | 3.08 (0.90, 5.23) | 1.25 (-0.55, 3.10) | 0.26 (-1.60, 2.21) | 0.61 (-0.55, 1.83) |
| Northern Bobwhite | Precipitation | 1.17 (0.61, 1.73) | 1.26 (0.81, 1.70) | 0.28 (-0.35, 0.94) | -1.51 (-2.32, -0.70) | -0.34 (-1.13, 0.47) | -0.34 (-1.11, 0.36) | -1.60 (-2.48, -0.71) |
| Northern Bobwhite | Temperature | -3.59 (-4.33, -2.81) | -3.36 (-4.39, -2.49) | -4.17 (-5.19, -3.07) | -4.44 (-6.31, -2.44) | -4.50 (-7.16, -2.04) | -5.23 (-7.20, -3.53) | -6.38 (-8.80, -3.72) |
| Greater Sage-Grouse | Intercept | 122.23 (121.99, 122.46) | 149.33 (148.74, 149.92) | 174.53 (169.31, 179.07) | 165.43 (164.28, 166.71) | 127.91 (125.15, 130.92) | 163.82 (152.31, 175.50) | 193.88 (189.73, 198.28) |
| Greater Sage-Grouse | Year | -1.27 (-1.75, -0.80) | -0.82 (-1.34, -0.29) | -1.06 (-1.56, -0.55) | -0.58 (-1.14, -0.04) | -0.40 (-1.05, 0.30) | 0.98 (0.31, 1.66) | 0.67 (-0.15, 1.48) |
| Greater Sage-Grouse | Latitude | 0.77 (0.26, 1.35) | 2.47 (1.89, 3.09) | 2.92 (2.41, 3.44) | 3.40 (2.72, 4.10) | 3.16 (2.32, 4.02) | 2.78 (2.11, 3.45) | 2.56 (1.88, 3.27) |
| Greater Sage-Grouse | Longitude | 3.73 (1.51, 5.95) | 2.76 (0.82, 4.69) | 0.96 (-0.85, 2.63) | -2.99 (-4.83, -1.15) | -4.49 (-5.71, -3.28) | -3.05 (-4.31, -1.80) | -5.62 (-6.86, -4.35) |
| Greater Sage-Grouse | Mid green-up | 1.79 (1.46, 2.13) | 1.39 (1.06, 1.73) | 1.26 (0.92, 1.59) | 1.42 (1.00, 1.83) | 1.59 (1.16, 2.02) | 2.16 (1.70, 2.61) | 2.06 (1.49, 2.62) |
| Greater Sage-Grouse | Precipitation | 0.42 (-0.18, 1.01) | 0.92 (0.44, 1.40) | 1.65 (1.14, 2.16) | 1.60 (1.08, 2.12) | 1.38 (0.76, 1.95) | 0.85 (0.30, 1.39) | 0.47 (-0.22, 1.16) |
| Greater Sage-Grouse | Temperature | -1.85 (-2.27, -1.42) | -1.70 (-2.12, -1.28) | -0.70 (-1.15, -0.23) | -0.44 (-0.96, 0.08) | -0.86 (-1.42, -0.30) | -2.25 (-2.75, -1.75) | -1.80 (-2.39, -1.22) |
| Sharp-tailed Grouse | Intercept | 152.50 (152.33, 152.67) | 214.75 (208.04, 221.31) | 124.41 (113.69, 134.46) | 155.88 (152.65, 158.85) | 175.22 (172.43, 177.87) | 190.91 (182.96, 199.70) | 147.33 (146.57, 148.08) |
| Sharp-tailed Grouse | Year | -4.04 (-4.66, -3.40) | -4.29 (-4.78, -3.79) | -4.11 (-4.74, -3.52) | -2.84 (-3.32, -2.37) | -3.18 (-3.80, -2.55) | -2.96 (-3.48, -2.39) | -1.71 (-2.31, -1.10) |
| Sharp-tailed Grouse | Latitude | -1.89 (-2.71, -1.07) | -2.40 (-3.07, -1.71) | -2.65 (-3.38, -1.95) | -1.89 (-2.76, -1.01) | 1.27 (0.21, 2.31) | 2.51 (1.45, 3.51) | 3.70 (2.73, 4.71) |
| Sharp-tailed Grouse | Longitude | -2.60 (-3.48, -1.69) | -2.42 (-3.22, -1.62) | 0.49 (-0.72, 1.69) | 2.66 (1.58, 3.72) | 2.24 (0.81, 3.66) | 0.07 (-1.29, 1.44) | 0.93 (-0.49, 2.39) |
| Sharp-tailed Grouse | Mid green-up | -0.83 (-1.54, -0.07) | -0.24 (-0.82, 0.32) | -0.84 (-1.53, -0.16) | -0.11 (-1.02, 0.78) | 0.43 (-0.69, 1.54) | 0.53 (-0.61, 1.67) | 1.13 (-0.23, 2.50) |
| Sharp-tailed Grouse | Precipitation | -0.70 (-1.45, 0.04) | -0.81 (-1.43, -0.21) | -1.43 (-2.13, -0.70) | -1.36 (-1.89, -0.84) | -0.69 (-1.45, 0.09) | 0.20 (-0.42, 0.82) | 0.33 (-0.44, 1.19) |
| Sharp-tailed Grouse | Temperature | -2.48 (-2.84, -2.11) | -2.38 (-2.73, -2.04) | -2.66 (-3.12, -2.18) | -2.48 (-3.17, -1.81) | -2.30 (-3.00, -1.58) | -1.45 (-2.16, -0.76) | -2.24 (-3.07, -1.33) |
| Greater Prairie-Chicken | Intercept | 130.16 (129.13, 131.23) | 117.52 (114.89, 119.90) | 138.74 (124.02, 153.78) | 179.57 (175.83, 185.27) | 141.63 (133.10, 150.33) | 158.57 (155.95, 161.22) | 174.66 (173.80, 175.51) |
| Greater Prairie-Chicken | Year | 1.42 (0.68, 2.32) | 1.73 (1.02, 2.59) | 1.71 (0.99, 2.48) | 1.31 (0.30, 2.29) | 1.03 (0.15, 1.86) | 1.15 (0.17, 2.03) | 1.52 (0.67, 2.52) |
| Greater Prairie-Chicken | Latitude | 3.92 (3.01, 4.81) | 2.94 (1.96, 3.85) | 1.58 (0.31, 2.89) | 0.15 (-1.60, 1.96) | -2.09 (-3.78, -0.28) | -3.25 (-4.84, -1.66) | -2.83 (-4.88, -0.95) |
| Greater Prairie-Chicken | Longitude | -1.33 (-2.28, -0.34) | -1.89 (-2.88, -0.84) | -2.60 (-3.87, -1.41) | -5.81 (-7.29, -4.31) | -5.16 (-7.16, -3.05) | -3.82 (-5.93, -1.44) | -0.21 (-2.04, 1.54) |
| Greater Prairie-Chicken | Mid green-up | 0.09 (-1.00, 1.28) | 1.17 (0.18, 2.26) | 2.37 (0.85, 3.86) | 5.44 (3.07, 7.65) | 6.63 (4.43, 8.67) | 6.04 (4.13, 7.97) | 4.94 (3.03, 7.38) |
| Greater Prairie-Chicken | Precipitation | 0.29 (-0.04, 0.61) | 0.18 (-0.22, 0.59) | 0.39 (0.03, 0.74) | 0.02 (-0.42, 0.50) | 0.53 (-0.03, 1.08) | -0.06 (-0.53, 0.43) | -0.23 (-0.74, 0.26) |
| Greater Prairie-Chicken | Temperature | -1.71 (-2.30, -1.12) | -1.84 (-2.43, -1.28) | -1.61 (-2.24, -0.95) | -1.87 (-2.72, -0.99) | -0.92 (-1.86, 0.00) | -2.31 (-3.33, -1.29) | -2.71 (-3.69, -1.71) |
| Lesser Prairie-Chicken | Intercept | 161.47 (161.28, 161.66) | 210.43 (202.95, 218.27) | 124.75 (114.97, 135.02) | 161.59 (158.42, 164.79) | 182.98 (181.51, 184.43) | 149.03 (147.35, 150.52) | 153.05 (152.15, 153.91) |
| Lesser Prairie-Chicken | Year | 2.23 (1.30, 3.15) | 2.43 (1.48, 3.44) | 4.38 (3.46, 5.30) | 5.25 (4.52, 5.98) | 5.28 (4.27, 6.20) | 3.36 (2.35, 4.40) | 1.86 (0.83, 2.91) |
| Lesser Prairie-Chicken | Latitude | 1.46 (-0.16, 3.08) | 3.62 (1.92, 5.53) | 6.38 (4.58, 8.23) | 5.31 (3.36, 7.26) | 6.37 (4.05, 8.67) | 4.22 (2.16, 6.34) | 6.49 (4.23, 8.77) |
| Lesser Prairie-Chicken | Longitude | -3.73 (-7.58, 0.13) | -8.69 (-13.24, -4.46) | -18.56 (-23.22, -14.13) | -19.58 (-24.59, -14.83) | -13.19 (-19.04, -7.95) | -7.83 (-13.04, -2.92) | -10.85 (-15.40, -6.44) |
| Lesser Prairie-Chicken | Mid green-up | 2.08 (1.13, 3.06) | 2.49 (1.60, 3.25) | 2.06 (0.91, 3.20) | 2.69 (1.71, 3.67) | 1.61 (0.22, 3.10) | 1.53 (-0.15, 3.07) | 3.74 (2.33, 5.18) |
| Lesser Prairie-Chicken | Precipitation | -2.04 (-2.50, -1.56) | -1.69 (-2.16, -1.22) | -1.18 (-1.63, -0.73) | -1.56 (-2.06, -1.07) | -1.38 (-2.11, -0.63) | -0.06 (-0.80, 0.66) | -0.39 (-1.02, 0.26) |
| Lesser Prairie-Chicken | Temperature | -0.35 (-1.11, 0.44) | 0.26 (-0.65, 1.16) | -1.29 (-2.19, -0.38) | -1.70 (-2.69, -0.71) | -3.87 (-4.97, -2.79) | -0.82 (-2.00, 0.31) | -1.00 (-2.13, 0.11) |
| Gray Partridge | Intercept | 131.28 (130.21, 132.24) | 130.04 (124.67, 135.41) | 143.12 (140.87, 145.32) | 178.55 (177.13, 180.10) | 148.11 (145.63, 150.62) | 156.02 (155.31, 156.69) | 214.61 (212.35, 216.90) |
| Gray Partridge | Year | -1.64 (-2.50, -0.81) | -0.81 (-1.68, -0.03) | -0.57 (-1.25, 0.06) | 0.10 (-0.56, 0.75) | 0.65 (-0.23, 1.53) | 0.12 (-0.77, 1.02) | 0.62 (-0.31, 1.56) |
| Gray Partridge | Latitude | 5.95 (4.86, 7.07) | 4.95 (4.00, 5.97) | 3.96 (3.12, 4.80) | 3.36 (2.41, 4.29) | 1.88 (0.67, 3.13) | 0.89 (-0.49, 2.15) | -1.36 (-3.16, 0.37) |
| Gray Partridge | Longitude | 1.02 (0.34, 1.68) | 0.93 (0.19, 1.71) | 0.93 (0.04, 1.87) | 1.14 (0.08, 2.23) | -0.02 (-1.06, 1.03) | 0.09 (-0.86, 1.04) | -0.95 (-2.07, 0.19) |
| Gray Partridge | Mid green-up | 0.96 (0.38, 1.62) | 1.16 (0.54, 1.88) | 0.95 (0.32, 1.61) | 0.84 (0.11, 1.56) | 0.07 (-1.00, 1.01) | -0.16 (-1.34, 0.89) | -0.01 (-1.28, 1.19) |
| Gray Partridge | Precipitation | -0.48 (-0.79, -0.17) | -0.48 (-0.79, -0.18) | -0.54 (-0.86, -0.22) | -1.02 (-1.51, -0.56) | -0.90 (-1.55, -0.29) | -0.84 (-1.50, -0.21) | -0.68 (-1.32, -0.07) |
| Gray Partridge | Temperature | -1.65 (-2.00, -1.33) | -1.55 (-1.90, -1.18) | -1.43 (-1.87, -0.99) | -1.51 (-2.05, -0.95) | -1.33 (-1.89, -0.76) | -1.18 (-1.73, -0.63) | -0.96 (-1.58, -0.32) |
| Ring-necked Pheasant | Intercept | 169.92 (169.74, 170.09) | 110.92 (107.65, 114.11) | 131.27 (121.98, 140.84) | 169.86 (165.61, 173.74) | 185.69 (184.46, 187.20) | 150.26 (148.38, 152.10) | 160.09 (159.28, 160.91) |
| Ring-necked Pheasant | Year | 1.08 (0.19, 1.87) | 1.08 (0.09, 2.11) | 0.35 (-0.58, 1.22) | 0.80 (-0.22, 1.84) | 0.71 (-0.62, 2.03) | -1.28 (-2.29, -0.21) | -4.53 (-5.73, -2.94) |
| Ring-necked Pheasant | Latitude | 6.73 (4.44, 9.37) | 6.78 (4.61, 8.99) | 2.64 (-0.67, 6.32) | 1.31 (-1.91, 4.30) | -5.95 (-9.74, -2.46) | 0.05 (-3.34, 3.45) | -8.02 (-12.35, -4.11) |
| Ring-necked Pheasant | Longitude | 1.75 (-0.66, 4.24) | 2.71 (0.28, 5.34) | 0.91 (-2.58, 4.47) | -0.86 (-4.28, 2.43) | -2.61 (-6.22, 1.13) | -3.93 (-7.21, -0.73) | -12.39 (-16.42, -8.09) |
| Ring-necked Pheasant | Mid green-up | -1.43 (-1.97, -0.86) | -1.56 (-2.34, -0.77) | -0.77 (-1.52, -0.01) | 0.00 (-0.75, 0.73) | 0.26 (-0.65, 1.20) | 0.97 (-0.09, 2.01) | 1.87 (1.06, 2.69) |
| Ring-necked Pheasant | Precipitation | 2.29 (1.79, 2.79) | 1.35 (0.81, 1.90) | 1.25 (0.54, 1.91) | 1.05 (0.47, 1.64) | 2.06 (1.11, 3.00) | 1.70 (0.98, 2.39) | 1.93 (0.99, 2.96) |
| Ring-necked Pheasant | Temperature | -0.95 (-1.57, -0.31) | -1.00 (-1.65, -0.39) | -1.16 (-1.90, -0.38) | -1.47 (-2.20, -0.77) | -3.33 (-4.13, -2.43) | -0.99 (-1.88, -0.02) | 0.20 (-0.76, 1.02) |
| Mountain Plover | Intercept | 167.63 (167.43, 167.83) | 116.19 (114.05, 117.97) | 139.32 (132.86, 145.87) | 162.96 (156.90, 167.84) | 140.59 (139.76, 141.40) | 146.39 (144.13, 148.55) | 183.46 (179.40, 187.54) |
| Mountain Plover | Year | 8.20 (4.92, 11.94) | 7.09 (3.74, 10.42) | 4.70 (1.84, 7.72) | 7.67 (3.71, 11.94) | 14.49 (8.69, 20.71) | 8.00 (3.78, 12.40) | 12.66 (7.22, 18.11) |
| Mountain Plover | Latitude | 10.05 (5.15, 14.76) | 8.08 (3.53, 12.93) | 7.38 (3.29, 11.58) | 7.92 (3.08, 13.00) | 8.50 (2.31, 15.03) | 8.07 (1.29, 14.96) | 10.60 (2.28, 19.05) |
| Mountain Plover | Longitude | 1.37 (-3.77, 6.58) | 0.66 (-4.96, 6.31) | 0.39 (-7.03, 7.87) | 0.35 (-8.71, 8.93) | 1.61 (-5.89, 9.59) | 0.68 (-5.53, 6.76) | 0.82 (-7.99, 10.03) |
| Mountain Plover | Mid green-up | 1.13 (-2.42, 4.64) | 0.97 (-2.69, 4.73) | -0.28 (-3.85, 3.14) | -0.77 (-5.12, 3.51) | 0.19 (-5.73, 6.06) | -0.38 (-6.83, 6.05) | 0.56 (-6.35, 7.47) |
| Mountain Plover | Precipitation | -4.13 (-5.27, -3.01) | -3.28 (-4.64, -1.99) | -1.86 (-3.30, -0.58) | -3.38 (-5.22, -1.57) | -5.07 (-7.84, -2.35) | -5.02 (-8.16, -1.89) | -5.83 (-8.69, -3.13) |
| Mountain Plover | Temperature | -6.12 (-7.34, -4.67) | -5.83 (-7.22, -4.52) | -6.25 (-7.18, -5.28) | -3.92 (-5.70, -2.21) | -6.04 (-8.26, -3.75) | -4.71 (-6.31, -3.12) | -4.00 (-6.02, -2.13) |
| Upland Sandpiper | Intercept | 169.39 (168.28, 170.61) | 119.83 (119.16, 120.54) | 140.62 (139.91, 141.32) | 150.36 (148.66, 152.26) | 170.09 (156.23, 183.84) | 133.11 (132.42, 133.80) | 154.55 (153.40, 155.78) |
| Upland Sandpiper | Year | -3.81 (-4.31, -3.28) | -3.82 (-4.27, -3.35) | -3.54 (-4.08, -3.03) | -3.10 (-3.71, -2.51) | -2.33 (-3.10, -1.59) | -1.72 (-2.50, -0.95) | -0.74 (-1.62, 0.07) |
| Upland Sandpiper | Latitude | 5.19 (3.94, 6.76) | 4.88 (3.59, 6.17) | 4.17 (2.66, 5.64) | 2.36 (0.17, 4.27) | 0.16 (-2.01, 2.22) | 0.37 (-2.49, 2.85) | -0.90 (-2.74, 1.03) |
| Upland Sandpiper | Longitude | -2.00 (-3.17, -0.71) | -1.44 (-2.70, -0.21) | -1.38 (-2.91, 0.17) | -2.30 (-4.44, -0.25) | -3.00 (-5.14, -0.86) | -1.83 (-4.65, 0.66) | -4.22 (-6.10, -2.24) |
| Upland Sandpiper | Mid green-up | 1.27 (0.33, 2.16) | 1.52 (0.86, 2.12) | 1.21 (0.42, 2.01) | 1.17 (0.31, 2.04) | 0.40 (-0.66, 1.54) | 0.78 (-0.44, 1.98) | 0.88 (-0.10, 1.85) |
| Upland Sandpiper | Precipitation | -1.02 (-1.60, -0.48) | -0.48 (-0.97, -0.03) | -0.36 (-0.87, 0.14) | -0.33 (-0.87, 0.23) | -0.41 (-1.12, 0.30) | 0.34 (-0.35, 1.04) | 0.32 (-0.36, 1.00) |
| Upland Sandpiper | Temperature | -0.83 (-1.23, -0.43) | -0.83 (-1.20, -0.46) | -0.97 (-1.41, -0.54) | -1.48 (-2.01, -0.95) | -1.84 (-2.56, -1.11) | -1.70 (-2.44, -0.97) | -0.75 (-1.56, 0.12) |
| Long-billed Curlew | Intercept | 112.39 (112.17, 112.62) | 133.00 (130.67, 135.45) | 145.83 (138.44, 154.41) | 118.52 (117.08, 119.85) | 149.04 (147.98, 150.09) | 171.79 (167.98, 175.07) | 188.53 (184.25, 193.25) |
| Long-billed Curlew | Year | 3.19 (0.04, 6.35) | 1.30 (-1.37, 4.10) | -0.83 (-3.23, 1.77) | -1.44 (-4.06, 1.14) | -2.78 (-5.96, 0.22) | -2.01 (-5.55, 1.00) | -1.80 (-6.17, 1.48) |
| Long-billed Curlew | Latitude | 2.40 (0.88, 4.09) | 2.69 (1.18, 4.19) | 2.28 (0.79, 3.69) | 2.42 (1.00, 3.82) | 2.52 (0.38, 4.43) | 3.19 (0.74, 5.41) | 3.40 (0.15, 6.29) |
| Long-billed Curlew | Longitude | 4.08 (1.82, 6.35) | 4.09 (2.04, 6.22) | 5.51 (3.21, 7.75) | 4.43 (2.14, 6.78) | 0.52 (-2.08, 3.24) | -2.59 (-5.07, 0.15) | -3.72 (-6.19, -0.88) |
| Long-billed Curlew | Mid green-up | 2.06 (1.09, 3.06) | 2.18 (1.28, 3.02) | 1.35 (0.46, 2.26) | 0.90 (0.04, 1.73) | 0.75 (-0.37, 1.81) | -0.09 (-1.62, 1.60) | 0.52 (-0.55, 1.60) |
| Long-billed Curlew | Precipitation | -1.12 (-2.39, 0.06) | -0.88 (-2.05, 0.33) | -0.08 (-1.54, 1.32) | 0.62 (-0.76, 1.97) | 0.50 (-1.34, 2.33) | 1.12 (-1.03, 3.18) | 1.14 (-0.92, 3.43) |
| Long-billed Curlew | Temperature | -2.73 (-4.40, -1.16) | -2.53 (-4.01, -1.07) | -2.48 (-4.22, -0.76) | -2.51 (-4.42, -0.61) | -2.18 (-4.58, 0.15) | -1.60 (-4.07, 0.88) | -1.52 (-4.50, 1.39) |
| Marbled Godwit | Intercept | 121.25 (120.95, 121.55) | 144.63 (142.07, 147.18) | 148.08 (139.36, 159.18) | 123.15 (121.28, 125.19) | 159.76 (158.46, 161.06) | 181.07 (179.07, 183.03) | 179.50 (178.02, 180.96) |
| Marbled Godwit | Year | -1.64 (-2.50, -0.81) | -0.81 (-1.68, -0.03) | -0.57 (-1.25, 0.06) | 0.10 (-0.56, 0.75) | 0.65 (-0.23, 1.53) | 0.12 (-0.77, 1.02) | 0.62 (-0.31, 1.56) |
| Marbled Godwit | Latitude | 5.95 (4.86, 7.07) | 4.95 (4.00, 5.97) | 3.96 (3.12, 4.80) | 3.36 (2.41, 4.29) | 1.88 (0.67, 3.13) | 0.89 (-0.49, 2.15) | -1.36 (-3.16, 0.37) |
| Marbled Godwit | Longitude | 1.02 (0.34, 1.68) | 0.93 (0.19, 1.71) | 0.93 (0.04, 1.87) | 1.14 (0.08, 2.23) | -0.02 (-1.06, 1.03) | 0.09 (-0.86, 1.04) | -0.95 (-2.07, 0.19) |
| Marbled Godwit | Mid green-up | 0.96 (0.38, 1.62) | 1.16 (0.54, 1.88) | 0.95 (0.32, 1.61) | 0.84 (0.11, 1.56) | 0.07 (-1.00, 1.01) | -0.16 (-1.34, 0.89) | -0.01 (-1.28, 1.19) |
| Marbled Godwit | Precipitation | -0.48 (-0.79, -0.17) | -0.48 (-0.79, -0.18) | -0.54 (-0.86, -0.22) | -1.02 (-1.51, -0.56) | -0.90 (-1.55, -0.29) | -0.84 (-1.50, -0.21) | -0.68 (-1.32, -0.07) |
| Marbled Godwit | Temperature | -1.65 (-2.00, -1.33) | -1.55 (-1.90, -1.18) | -1.43 (-1.87, -0.99) | -1.51 (-2.05, -0.95) | -1.33 (-1.89, -0.76) | -1.18 (-1.73, -0.63) | -0.96 (-1.58, -0.32) |
| Wilson's Phalarope | Intercept | 108.37 (108.14, 108.61) | 120.78 (118.76, 122.84) | 143.45 (136.43, 150.88) | 170.57 (167.07, 175.37) | 143.17 (142.40, 143.94) | 153.69 (151.31, 155.88) | 184.97 (180.93, 188.85) |
| Wilson's Phalarope | Year | -4.30 (-5.22, -3.33) | -3.76 (-4.71, -2.81) | -3.88 (-5.02, -2.74) | -4.61 (-5.87, -3.40) | -5.11 (-6.42, -3.83) | -4.03 (-5.21, -2.74) | -2.96 (-4.30, -1.76) |
| Wilson's Phalarope | Latitude | 0.68 (-1.75, 3.64) | 1.45 (-1.19, 4.20) | 2.37 (-0.26, 5.12) | 4.93 (2.07, 7.77) | 5.94 (2.09, 9.58) | 4.34 (-0.15, 8.69) | 1.52 (-3.60, 6.64) |
| Wilson's Phalarope | Longitude | 0.44 (-2.45, 3.86) | 0.37 (-2.02, 3.22) | 0.08 (-2.24, 2.68) | -1.54 (-4.61, 1.49) | -0.92 (-4.75, 2.80) | -0.20 (-4.15, 3.26) | -1.85 (-6.47, 2.22) |
| Wilson's Phalarope | Mid green-up | 3.25 (1.75, 4.76) | 3.93 (2.65, 5.17) | 3.18 (1.97, 4.41) | 2.84 (1.36, 4.38) | 3.72 (1.38, 5.99) | 2.60 (0.44, 4.94) | 1.31 (-0.73, 3.54) |
| Wilson's Phalarope | Precipitation | -0.81 (-1.63, 0.00) | -0.81 (-1.80, 0.10) | -1.51 (-2.60, -0.38) | -2.23 (-3.65, -0.81) | -0.86 (-2.19, 0.45) | 0.73 (-0.67, 2.05) | 1.02 (-0.06, 2.13) |
| Wilson's Phalarope | Temperature | -0.48 (-1.35, 0.37) | -1.24 (-2.13, -0.33) | -1.35 (-2.44, -0.28) | -0.79 (-1.99, 0.36) | -0.44 (-1.61, 0.73) | -0.62 (-1.87, 0.60) | -0.65 (-1.70, 0.45) |
| Willet | Intercept | 151.37 (151.18, 151.56) | 165.79 (164.86, 166.75) | 126.48 (119.59, 133.08) | 131.75 (128.37, 135.35) | 189.80 (188.75, 190.88) | 138.16 (136.60, 139.61) | 143.97 (140.22, 147.83) |
| Willet | Year | -2.71 (-4.17, -1.09) | -2.26 (-3.62, -0.92) | -0.81 (-2.15, 0.54) | 2.06 (0.37, 3.76) | 6.47 (3.94, 8.92) | 10.86 (9.03, 12.70) | 12.78 (11.07, 14.50) |
| Willet | Latitude | 3.87 (1.45, 6.15) | 2.63 (0.01, 5.25) | 2.41 (-1.01, 5.70) | 1.65 (-2.23, 5.42) | -4.04 (-8.84, 1.07) | -4.34 (-8.79, -0.11) | -5.35 (-10.57, -0.78) |
| Willet | Longitude | 2.13 (0.61, 3.60) | 1.93 (0.31, 3.60) | 2.22 (-0.06, 4.47) | 1.11 (-1.19, 3.37) | -0.87 (-3.39, 1.76) | 2.08 (-0.28, 4.33) | 1.96 (-0.26, 4.03) |
| Willet | Mid green-up | -0.29 (-1.37, 0.89) | 1.28 (-0.64, 3.26) | 4.41 (2.17, 6.65) | 6.47 (3.66, 9.22) | 6.13 (3.30, 8.92) | 6.11 (3.52, 8.76) | 5.18 (3.00, 7.60) |
| Willet | Precipitation | -1.15 (-1.96, -0.35) | -1.37 (-2.20, -0.56) | -1.05 (-2.01, -0.04) | -0.36 (-1.62, 1.00) | 1.79 (0.09, 3.45) | 2.10 (0.37, 3.88) | 1.95 (0.49, 3.47) |
| Willet | Temperature | 0.04 (-0.68, 0.76) | -0.40 (-1.16, 0.40) | -0.85 (-2.01, 0.26) | -0.29 (-1.48, 0.92) | 0.59 (-0.91, 1.95) | 0.25 (-1.11, 1.58) | -0.17 (-1.18, 0.88) |
| American Bittern | Intercept | 134.49 (133.18, 135.70) | 134.45 (128.63, 140.12) | 149.62 (147.73, 151.59) | 141.08 (139.18, 143.11) | 150.72 (147.65, 153.74) | 173.18 (172.48, 173.87) | 220.00 (218.07, 221.89) |
| American Bittern | Year | -1.64 (-2.50, -0.81) | -0.81 (-1.68, -0.03) | -0.57 (-1.25, 0.06) | 0.10 (-0.56, 0.75) | 0.65 (-0.23, 1.53) | 0.12 (-0.77, 1.02) | 0.62 (-0.31, 1.56) |
| American Bittern | Latitude | 5.95 (4.86, 7.07) | 4.95 (4.00, 5.97) | 3.96 (3.12, 4.80) | 3.36 (2.41, 4.29) | 1.88 (0.67, 3.13) | 0.89 (-0.49, 2.15) | -1.36 (-3.16, 0.37) |
| American Bittern | Longitude | 1.02 (0.34, 1.68) | 0.93 (0.19, 1.71) | 0.93 (0.04, 1.87) | 1.14 (0.08, 2.23) | -0.02 (-1.06, 1.03) | 0.09 (-0.86, 1.04) | -0.95 (-2.07, 0.19) |
| American Bittern | Mid green-up | 0.96 (0.38, 1.62) | 1.16 (0.54, 1.88) | 0.95 (0.32, 1.61) | 0.84 (0.11, 1.56) | 0.07 (-1.00, 1.01) | -0.16 (-1.34, 0.89) | -0.01 (-1.28, 1.19) |
| American Bittern | Precipitation | -0.48 (-0.79, -0.17) | -0.48 (-0.79, -0.18) | -0.54 (-0.86, -0.22) | -1.02 (-1.51, -0.56) | -0.90 (-1.55, -0.29) | -0.84 (-1.50, -0.21) | -0.68 (-1.32, -0.07) |
| American Bittern | Temperature | -1.65 (-2.00, -1.33) | -1.55 (-1.90, -1.18) | -1.43 (-1.87, -0.99) | -1.51 (-2.05, -0.95) | -1.33 (-1.89, -0.76) | -1.18 (-1.73, -0.63) | -0.96 (-1.58, -0.32) |
| Northern Harrier | Intercept | 135.71 (135.35, 136.07) | 152.74 (150.22, 155.39) | 119.50 (117.80, 121.21) | 138.91 (136.41, 141.35) | 175.11 (173.52, 176.64) | 183.67 (181.63, 186.04) | 183.85 (182.52, 185.27) |
| Northern Harrier | Year | -1.64 (-2.50, -0.81) | -0.81 (-1.68, -0.03) | -0.57 (-1.25, 0.06) | 0.10 (-0.56, 0.75) | 0.65 (-0.23, 1.53) | 0.12 (-0.77, 1.02) | 0.62 (-0.31, 1.56) |
| Northern Harrier | Latitude | 5.95 (4.86, 7.07) | 4.95 (4.00, 5.97) | 3.96 (3.12, 4.80) | 3.36 (2.41, 4.29) | 1.88 (0.67, 3.13) | 0.89 (-0.49, 2.15) | -1.36 (-3.16, 0.37) |
| Northern Harrier | Longitude | 1.02 (0.34, 1.68) | 0.93 (0.19, 1.71) | 0.93 (0.04, 1.87) | 1.14 (0.08, 2.23) | -0.02 (-1.06, 1.03) | 0.09 (-0.86, 1.04) | -0.95 (-2.07, 0.19) |
| Northern Harrier | Mid green-up | 0.96 (0.38, 1.62) | 1.16 (0.54, 1.88) | 0.95 (0.32, 1.61) | 0.84 (0.11, 1.56) | 0.07 (-1.00, 1.01) | -0.16 (-1.34, 0.89) | -0.01 (-1.28, 1.19) |
| Northern Harrier | Precipitation | -0.48 (-0.79, -0.17) | -0.48 (-0.79, -0.18) | -0.54 (-0.86, -0.22) | -1.02 (-1.51, -0.56) | -0.90 (-1.55, -0.29) | -0.84 (-1.50, -0.21) | -0.68 (-1.32, -0.07) |
| Northern Harrier | Temperature | -1.65 (-2.00, -1.33) | -1.55 (-1.90, -1.18) | -1.43 (-1.87, -0.99) | -1.51 (-2.05, -0.95) | -1.33 (-1.89, -0.76) | -1.18 (-1.73, -0.63) | -0.96 (-1.58, -0.32) |
| Short-eared Owl | Intercept | 165.44 (165.16, 165.72) | 119.76 (114.80, 125.17) | 133.01 (131.17, 134.92) | 175.02 (173.07, 177.06) | 143.53 (140.04, 146.50) | 144.15 (143.54, 144.75) | 209.86 (207.56, 212.14) |
| Short-eared Owl | Year | -1.64 (-2.50, -0.81) | -0.81 (-1.68, -0.03) | -0.57 (-1.25, 0.06) | 0.10 (-0.56, 0.75) | 0.65 (-0.23, 1.53) | 0.12 (-0.77, 1.02) | 0.62 (-0.31, 1.56) |
| Short-eared Owl | Latitude | 5.95 (4.86, 7.07) | 4.95 (4.00, 5.97) | 3.96 (3.12, 4.80) | 3.36 (2.41, 4.29) | 1.88 (0.67, 3.13) | 0.89 (-0.49, 2.15) | -1.36 (-3.16, 0.37) |
| Short-eared Owl | Longitude | 1.02 (0.34, 1.68) | 0.93 (0.19, 1.71) | 0.93 (0.04, 1.87) | 1.14 (0.08, 2.23) | -0.02 (-1.06, 1.03) | 0.09 (-0.86, 1.04) | -0.95 (-2.07, 0.19) |
| Short-eared Owl | Mid green-up | 0.96 (0.38, 1.62) | 1.16 (0.54, 1.88) | 0.95 (0.32, 1.61) | 0.84 (0.11, 1.56) | 0.07 (-1.00, 1.01) | -0.16 (-1.34, 0.89) | -0.01 (-1.28, 1.19) |
| Short-eared Owl | Precipitation | -0.48 (-0.79, -0.17) | -0.48 (-0.79, -0.18) | -0.54 (-0.86, -0.22) | -1.02 (-1.51, -0.56) | -0.90 (-1.55, -0.29) | -0.84 (-1.50, -0.21) | -0.68 (-1.32, -0.07) |
| Short-eared Owl | Temperature | -1.65 (-2.00, -1.33) | -1.55 (-1.90, -1.18) | -1.43 (-1.87, -0.99) | -1.51 (-2.05, -0.95) | -1.33 (-1.89, -0.76) | -1.18 (-1.73, -0.63) | -0.96 (-1.58, -0.32) |
| Horned Lark | Intercept | 137.55 (137.33, 137.76) | 160.09 (159.22, 160.99) | 120.93 (113.48, 128.21) | 119.30 (115.70, 123.13) | 180.24 (179.09, 181.34) | 190.45 (189.52, 191.38) | 130.07 (127.25, 132.94) |
| Horned Lark | Year | 4.95 (3.74, 6.34) | 6.39 (4.74, 8.06) | 5.84 (3.21, 8.51) | 2.98 (0.79, 5.26) | 2.45 (0.33, 4.50) | 2.60 (-0.01, 5.17) | 0.25 (-2.07, 2.51) |
| Horned Lark | Latitude | -0.01 (-1.25, 1.33) | 0.56 (-0.65, 1.86) | 3.86 (2.47, 5.14) | 1.33 (-0.39, 3.15) | -0.11 (-1.53, 1.21) | -1.49 (-2.71, -0.23) | -0.93 (-1.98, -0.02) |
| Horned Lark | Longitude | 1.72 (-0.13, 3.95) | 4.48 (1.74, 7.34) | 6.96 (3.31, 10.52) | 1.99 (-1.82, 6.08) | -1.68 (-5.56, 2.12) | -2.30 (-6.66, 1.84) | -7.22 (-12.24, -2.49) |
| Horned Lark | Mid green-up | -2.26 (-3.62, -1.03) | -2.97 (-4.78, -1.16) | -3.14 (-5.44, -0.76) | -0.83 (-2.44, 0.72) | 2.56 (0.72, 4.36) | 2.74 (0.16, 5.08) | 3.15 (1.36, 4.76) |
| Horned Lark | Precipitation | -0.47 (-1.42, 0.47) | -0.37 (-1.60, 0.88) | -0.40 (-1.76, 0.94) | -2.50 (-3.97, -1.04) | -5.14 (-6.53, -3.78) | -6.15 (-7.59, -4.65) | -5.70 (-6.83, -4.53) |
| Horned Lark | Temperature | -2.28 (-3.45, -1.15) | -1.76 (-3.01, -0.42) | 2.65 (0.62, 4.77) | 1.90 (0.16, 3.72) | 1.66 (0.06, 3.34) | -0.22 (-1.72, 1.35) | -0.76 (-1.93, 0.60) |
| Sedge Wren | Intercept | 160.11 (159.80, 160.41) | 110.88 (106.16, 115.71) | 126.30 (124.45, 128.20) | 165.11 (163.20, 166.96) | 210.17 (208.43, 212.24) | 138.22 (137.78, 138.66) | 199.05 (197.07, 201.07) |
| Sedge Wren | Year | -1.64 (-2.50, -0.81) | -0.81 (-1.68, -0.03) | -0.57 (-1.25, 0.06) | 0.10 (-0.56, 0.75) | 0.65 (-0.23, 1.53) | 0.12 (-0.77, 1.02) | 0.62 (-0.31, 1.56) |
| Sedge Wren | Latitude | 5.95 (4.86, 7.07) | 4.95 (4.00, 5.97) | 3.96 (3.12, 4.80) | 3.36 (2.41, 4.29) | 1.88 (0.67, 3.13) | 0.89 (-0.49, 2.15) | -1.36 (-3.16, 0.37) |
| Sedge Wren | Longitude | 1.02 (0.34, 1.68) | 0.93 (0.19, 1.71) | 0.93 (0.04, 1.87) | 1.14 (0.08, 2.23) | -0.02 (-1.06, 1.03) | 0.09 (-0.86, 1.04) | -0.95 (-2.07, 0.19) |
| Sedge Wren | Mid green-up | 0.96 (0.38, 1.62) | 1.16 (0.54, 1.88) | 0.95 (0.32, 1.61) | 0.84 (0.11, 1.56) | 0.07 (-1.00, 1.01) | -0.16 (-1.34, 0.89) | -0.01 (-1.28, 1.19) |
| Sedge Wren | Precipitation | -0.48 (-0.79, -0.17) | -0.48 (-0.79, -0.18) | -0.54 (-0.86, -0.22) | -1.02 (-1.51, -0.56) | -0.90 (-1.55, -0.29) | -0.84 (-1.50, -0.21) | -0.68 (-1.32, -0.07) |
| Sedge Wren | Temperature | -1.65 (-2.00, -1.33) | -1.55 (-1.90, -1.18) | -1.43 (-1.87, -0.99) | -1.51 (-2.05, -0.95) | -1.33 (-1.89, -0.76) | -1.18 (-1.73, -0.63) | -0.96 (-1.58, -0.32) |
| Sprague's Pipit | Intercept | 172.64 (171.58, 173.78) | 123.20 (122.56, 123.80) | 145.55 (144.72, 146.41) | 160.52 (158.03, 162.95) | 142.29 (141.60, 142.98) | 139.76 (138.86, 140.65) | 169.25 (168.24, 170.26) |
| Sprague's Pipit | Year | 2.59 (1.24, 3.82) | 1.46 (0.07, 2.74) | 0.33 (-0.90, 1.56) | 3.29 (1.29, 5.22) | 0.87 (-1.35, 3.08) | -2.25 (-4.63, -0.07) | -5.69 (-7.30, -3.80) |
| Sprague's Pipit | Latitude | 6.16 (4.17, 8.27) | 4.99 (2.52, 7.57) | 3.01 (-0.11, 6.14) | 5.94 (2.51, 9.46) | 9.74 (4.37, 15.34) | 9.68 (4.73, 14.96) | 8.86 (4.94, 12.91) |
| Sprague's Pipit | Longitude | -0.44 (-2.60, 1.65) | -0.65 (-2.77, 1.47) | 0.25 (-1.79, 2.28) | 5.07 (1.69, 8.64) | 1.78 (-2.33, 5.72) | -0.12 (-3.90, 3.63) | -2.00 (-5.37, 1.38) |
| Sprague's Pipit | Mid green-up | -1.48 (-2.94, -0.13) | -1.93 (-3.69, -0.26) | -1.90 (-3.78, 0.01) | -4.07 (-6.31, -1.89) | -3.92 (-7.37, -0.61) | -2.12 (-5.41, 0.88) | -2.77 (-5.24, -0.31) |
| Sprague's Pipit | Precipitation | -0.69 (-1.49, 0.13) | -0.61 (-1.40, 0.15) | -1.17 (-2.13, -0.21) | -5.13 (-6.71, -3.51) | -4.96 (-6.70, -3.11) | -4.12 (-6.40, -1.91) | -5.98 (-7.56, -4.35) |
| Sprague's Pipit | Temperature | -2.73 (-3.43, -2.03) | -2.77 (-3.46, -2.10) | -4.09 (-4.84, -3.33) | -8.45 (-9.58, -7.22) | -4.34 (-5.64, -3.03) | -1.40 (-2.61, -0.21) | 0.33 (-0.81, 1.57) |
| Chestnut-collared Longspur | Intercept | 149.12 (148.83, 149.42) | 177.65 (176.99, 178.36) | 122.71 (119.91, 125.38) | 142.97 (140.66, 145.33) | 180.84 (174.33, 187.13) | 140.58 (138.53, 142.75) | 147.00 (141.52, 152.64) |
| Chestnut-collared Longspur | Year | -0.56 (-1.10, -0.04) | -0.55 (-1.13, 0.03) | -0.59 (-1.22, 0.06) | -1.89 (-2.66, -1.12) | -2.20 (-2.99, -1.42) | -1.98 (-2.67, -1.32) | -1.40 (-1.99, -0.84) |
| Chestnut-collared Longspur | Latitude | 5.75 (3.29, 8.25) | 7.57 (5.00, 10.18) | 3.65 (1.30, 6.03) | 2.44 (0.03, 4.91) | -0.29 (-3.34, 2.83) | 0.78 (-1.62, 3.22) | 3.04 (1.03, 5.03) |
| Chestnut-collared Longspur | Longitude | 4.10 (2.71, 5.55) | 5.48 (4.15, 6.82) | 3.65 (2.03, 5.30) | 1.97 (0.19, 3.70) | 1.89 (0.03, 3.80) | 1.59 (-0.26, 3.45) | 1.02 (-0.40, 2.45) |
| Chestnut-collared Longspur | Mid green-up | -5.66 (-7.22, -4.10) | -6.29 (-7.84, -4.73) | -3.94 (-5.33, -2.61) | -1.20 (-2.48, 0.05) | -0.59 (-2.21, 0.97) | -2.20 (-3.25, -1.14) | -2.91 (-3.89, -1.87) |
| Chestnut-collared Longspur | Precipitation | -0.21 (-0.63, 0.22) | -0.60 (-1.09, -0.12) | -1.83 (-2.63, -1.04) | -1.51 (-2.28, -0.74) | -1.20 (-1.96, -0.44) | 0.42 (-0.29, 1.14) | 0.96 (0.32, 1.56) |
| Chestnut-collared Longspur | Temperature | -0.57 (-0.94, -0.21) | -0.28 (-0.68, 0.13) | -0.41 (-1.07, 0.26) | -0.05 (-0.77, 0.64) | 0.10 (-0.49, 0.69) | 0.00 (-0.56, 0.59) | -0.26 (-0.81, 0.31) |
| Thick-billed Longspur | Intercept | 124.28 (124.10, 124.47) | 150.38 (149.41, 151.35) | 166.97 (165.94, 167.98) | 109.22 (106.45, 112.49) | 166.74 (165.47, 167.97) | 181.99 (180.43, 183.58) | 127.63 (124.98, 130.16) |
| Thick-billed Longspur | Year | -2.66 (-6.55, 1.23) | -2.48 (-6.31, 1.15) | -2.41 (-6.32, 1.33) | -0.61 (-4.63, 3.40) | -0.65 (-6.20, 4.80) | 1.40 (-4.33, 7.38) | 5.37 (-0.41, 11.11) |
| Thick-billed Longspur | Latitude | 1.88 (0.24, 3.51) | 1.66 (-0.16, 3.52) | -1.30 (-3.72, 1.21) | -3.24 (-5.67, -0.70) | -5.45 (-7.83, -3.08) | -4.08 (-6.64, -1.61) | -7.32 (-9.85, -4.81) |
| Thick-billed Longspur | Longitude | 2.15 (-2.36, 6.60) | 2.20 (-2.74, 7.40) | 6.08 (-0.84, 13.20) | 4.91 (-2.67, 12.56) | 0.66 (-5.99, 7.32) | -1.52 (-7.49, 4.25) | -4.89 (-13.45, 3.32) |
| Thick-billed Longspur | Mid green-up | 0.65 (-0.81, 2.08) | 1.96 (0.33, 3.56) | 4.29 (2.04, 6.46) | 3.03 (0.59, 5.49) | 1.81 (-0.57, 4.17) | 1.80 (-0.86, 4.47) | 2.61 (-0.03, 5.31) |
| Thick-billed Longspur | Precipitation | -2.53 (-3.60, -1.51) | -2.12 (-3.40, -0.87) | -1.61 (-3.26, -0.03) | -3.32 (-5.07, -1.59) | -5.41 (-7.17, -3.76) | -3.80 (-6.05, -1.56) | -1.81 (-3.31, -0.30) |
| Thick-billed Longspur | Temperature | -0.61 (-1.49, 0.25) | -0.50 (-1.30, 0.32) | -0.49 (-1.91, 0.85) | -4.14 (-5.72, -2.54) | -5.26 (-6.54, -3.96) | -5.45 (-6.69, -4.21) | -5.83 (-6.87, -4.70) |
| Grasshopper Sparrow | Intercept | 131.10 (130.85, 131.34) | 165.02 (164.32, 165.71) | 187.26 (182.40, 192.77) | 129.61 (127.38, 131.73) | 145.27 (141.64, 149.07) | 180.63 (169.84, 190.90) | 135.79 (131.28, 140.38) |
| Grasshopper Sparrow | Year | 0.98 (0.50, 1.44) | 0.53 (0.08, 0.98) | -0.59 (-1.23, 0.05) | -0.23 (-1.10, 0.64) | -1.29 (-2.06, -0.52) | -0.74 (-1.51, 0.01) | 0.02 (-0.66, 0.66) |
| Grasshopper Sparrow | Latitude | 4.71 (4.37, 5.03) | 5.01 (4.69, 5.34) | 5.48 (4.97, 5.98) | 5.17 (4.53, 5.84) | 3.45 (2.82, 4.11) | 0.80 (0.07, 1.53) | -1.65 (-2.22, -1.03) |
| Grasshopper Sparrow | Longitude | -0.39 (-0.64, -0.15) | -0.28 (-0.51, -0.04) | -0.24 (-0.58, 0.08) | 0.13 (-0.33, 0.60) | 0.07 (-0.41, 0.55) | -0.60 (-1.02, -0.18) | -1.69 (-2.16, -1.18) |
| Grasshopper Sparrow | Mid green-up | 0.63 (0.32, 0.94) | 0.81 (0.47, 1.13) | 1.04 (0.67, 1.42) | 1.13 (0.57, 1.67) | 0.40 (-0.13, 0.91) | 0.58 (0.09, 1.04) | 1.12 (0.61, 1.61) |
| Grasshopper Sparrow | Precipitation | -0.65 (-0.92, -0.39) | -0.44 (-0.72, -0.16) | -0.18 (-0.47, 0.11) | 0.84 (0.47, 1.22) | 0.89 (0.47, 1.31) | 0.13 (-0.30, 0.54) | -0.22 (-0.63, 0.19) |
| Grasshopper Sparrow | Temperature | -0.63 (-1.27, 0.01) | -0.96 (-1.41, -0.48) | -0.14 (-0.72, 0.42) | -0.96 (-1.76, -0.16) | -2.15 (-2.83, -1.50) | -1.90 (-2.50, -1.23) | -1.09 (-1.79, -0.39) |
| Clay-colored Sparrow | Intercept | 125.41 (125.19, 125.64) | 157.07 (156.44, 157.72) | 180.64 (177.08, 184.71) | 126.44 (124.82, 127.90) | 135.87 (132.54, 139.25) | 165.87 (153.19, 178.78) | 195.20 (189.62, 201.29) |
| Clay-colored Sparrow | Year | 1.44 (0.98, 1.91) | 0.88 (0.40, 1.37) | 0.05 (-0.59, 0.70) | -0.13 (-1.04, 0.76) | -0.98 (-2.01, 0.02) | -4.21 (-5.13, -3.27) | -7.70 (-8.93, -6.55) |
| Clay-colored Sparrow | Latitude | 3.04 (1.68, 4.43) | 4.90 (3.47, 6.35) | 5.78 (3.83, 7.78) | 4.76 (2.20, 7.36) | 1.55 (-1.23, 4.45) | -11.08 (-14.03, -8.07) | -23.86 (-27.55, -20.46) |
| Clay-colored Sparrow | Longitude | -0.13 (-1.66, 1.47) | 1.64 (0.15, 3.16) | 2.86 (0.76, 4.95) | 2.49 (-0.03, 5.01) | 0.13 (-2.81, 3.20) | -3.97 (-7.30, -0.67) | -7.17 (-10.72, -3.79) |
| Clay-colored Sparrow | Mid green-up | 0.33 (-0.25, 0.90) | -0.27 (-0.75, 0.18) | -1.38 (-2.18, -0.57) | -3.50 (-4.62, -2.40) | -5.85 (-7.32, -4.46) | -6.31 (-7.79, -4.82) | -3.26 (-5.54, -1.14) |
| Clay-colored Sparrow | Precipitation | -0.29 (-0.57, -0.01) | -0.50 (-0.86, -0.14) | -1.13 (-1.56, -0.70) | -1.37 (-1.91, -0.79) | -0.23 (-0.86, 0.40) | 1.61 (0.98, 2.25) | 3.08 (2.37, 3.86) |
| Clay-colored Sparrow | Temperature | -1.42 (-1.69, -1.14) | -1.78 (-2.11, -1.46) | -2.61 (-2.99, -2.24) | -3.01 (-3.51, -2.52) | -1.02 (-1.59, -0.47) | 0.54 (0.07, 1.02) | 1.72 (1.19, 2.27) |
| Field Sparrow | Intercept | 139.47 (139.34, 139.59) | 169.97 (162.24, 177.76) | 174.02 (170.84, 177.14) | 149.05 (146.93, 151.18) | 140.75 (138.45, 143.00) | 180.70 (175.56, 185.96) | 209.27 (196.85, 220.81) |
| Field Sparrow | Year | -1.32 (-1.89, -0.74) | -2.24 (-2.90, -1.59) | -0.64 (-1.48, 0.15) | -1.11 (-2.09, -0.14) | -0.39 (-1.56, 0.76) | 0.84 (-0.14, 1.89) | 1.11 (0.37, 1.84) |
| Field Sparrow | Latitude | 1.05 (0.13, 2.05) | 0.95 (0.01, 1.93) | 0.37 (-1.04, 1.74) | -2.55 (-4.43, -0.74) | -3.31 (-5.36, -1.30) | -3.66 (-5.80, -1.45) | -1.07 (-2.50, 0.31) |
| Field Sparrow | Longitude | -4.89 (-6.38, -3.08) | -5.61 (-6.71, -4.45) | -3.43 (-4.90, -1.99) | -2.91 (-4.75, -0.98) | -3.75 (-6.09, -1.26) | -1.00 (-2.93, 0.80) | -2.04 (-4.04, -0.32) |
| Field Sparrow | Mid green-up | 2.80 (2.13, 3.48) | 2.52 (1.75, 3.32) | 2.52 (1.40, 3.64) | 4.17 (2.88, 5.47) | 3.76 (2.42, 5.13) | 5.87 (4.64, 7.11) | 5.46 (4.34, 6.59) |
| Field Sparrow | Precipitation | -0.31 (-0.52, -0.10) | -0.43 (-0.70, -0.17) | -0.99 (-1.41, -0.58) | -1.62 (-2.05, -1.17) | -2.21 (-2.71, -1.71) | -1.88 (-2.31, -1.52) | -2.40 (-2.68, -2.11) |
| Field Sparrow | Temperature | -4.13 (-4.77, -3.47) | -4.03 (-4.90, -3.15) | -4.44 (-5.48, -3.45) | -3.92 (-5.22, -2.65) | -4.48 (-5.88, -3.01) | -2.54 (-3.74, -1.24) | -2.72 (-3.72, -1.57) |
| Brewer's Sparrow | Intercept | 143.97 (142.91, 144.95) | 127.71 (125.67, 129.73) | 160.71 (143.23, 177.87) | 124.05 (121.66, 126.23) | 160.47 (149.49, 171.49) | 183.96 (178.61, 189.21) | 127.65 (127.04, 128.26) |
| Brewer's Sparrow | Year | -1.69 (-3.90, 0.34) | -0.53 (-2.70, 1.56) | 1.63 (-0.54, 3.89) | 1.31 (-1.47, 4.05) | -3.18 (-6.27, -0.03) | -5.57 (-8.06, -2.94) | -3.26 (-5.98, -0.63) |
| Brewer's Sparrow | Latitude | 6.52 (4.61, 8.45) | 6.04 (4.25, 7.96) | 4.76 (2.69, 6.76) | 4.45 (2.10, 6.86) | -0.05 (-2.46, 2.38) | -2.12 (-4.41, 0.00) | -3.25 (-4.97, -1.31) |
| Brewer's Sparrow | Longitude | 3.68 (0.06, 7.55) | 2.05 (-1.66, 5.92) | 0.40 (-3.59, 4.22) | 5.74 (1.34, 10.16) | -4.90 (-9.84, 0.03) | -2.06 (-6.30, 1.95) | -3.88 (-7.60, 0.04) |
| Brewer's Sparrow | Mid green-up | -0.77 (-2.34, 0.91) | 0.37 (-1.42, 2.14) | -0.32 (-2.28, 1.65) | -0.92 (-3.26, 1.43) | -1.54 (-4.18, 1.12) | -2.42 (-4.92, 0.03) | -3.22 (-5.05, -1.35) |
| Brewer's Sparrow | Precipitation | -0.86 (-2.13, 0.49) | -0.19 (-1.48, 1.04) | -1.18 (-2.50, 0.12) | -2.56 (-4.58, -0.59) | 1.03 (-0.80, 3.01) | 0.43 (-1.37, 2.19) | -2.09 (-3.46, -0.55) |
| Brewer's Sparrow | Temperature | -4.32 (-5.10, -3.50) | -3.33 (-4.23, -2.43) | -3.41 (-4.55, -2.30) | -7.56 (-9.26, -5.83) | -8.61 (-10.06, -7.16) | -8.50 (-10.13, -7.08) | -8.93 (-10.39, -7.57) |
| Lark Sparrow | Intercept | 115.49 (115.33, 115.64) | 137.63 (136.82, 138.48) | 161.07 (159.93, 162.24) | 105.45 (103.31, 107.40) | 154.78 (153.80, 155.77) | 165.22 (164.10, 166.37) | 186.35 (185.46, 187.22) |
| Lark Sparrow | Year | 3.29 (0.97, 5.43) | 2.11 (0.17, 4.06) | 0.95 (-1.16, 3.07) | -2.53 (-4.79, -0.30) | -2.70 (-5.82, 0.36) | 2.32 (-1.28, 5.65) | 1.37 (-2.97, 5.56) |
| Lark Sparrow | Latitude | 6.61 (5.80, 7.41) | 6.37 (5.70, 7.04) | 5.31 (4.52, 6.12) | 2.48 (1.55, 3.48) | 0.77 (-0.28, 1.93) | -0.59 (-1.78, 0.65) | -0.77 (-1.84, 0.45) |
| Lark Sparrow | Longitude | -3.11 (-4.42, -1.92) | -3.48 (-4.73, -2.22) | -3.94 (-5.62, -2.17) | -3.21 (-5.16, -1.20) | -1.88 (-4.49, 0.65) | 2.17 (-1.06, 5.20) | 0.77 (-2.75, 4.25) |
| Lark Sparrow | Mid green-up | -2.38 (-4.19, -0.61) | -1.85 (-3.78, 0.21) | 1.68 (-0.23, 3.92) | 3.51 (1.29, 5.71) | 5.06 (2.23, 7.93) | 5.00 (1.76, 8.57) | 4.52 (0.81, 8.44) |
| Lark Sparrow | Precipitation | -0.22 (-0.97, 0.61) | -0.17 (-1.04, 0.64) | -0.30 (-1.20, 0.59) | -0.47 (-1.36, 0.37) | -1.42 (-2.48, -0.34) | -1.12 (-2.17, -0.01) | -0.42 (-1.24, 0.48) |
| Lark Sparrow | Temperature | 1.18 (-0.03, 2.32) | 0.25 (-1.24, 1.79) | 1.92 (-0.01, 3.96) | 2.31 (0.21, 4.44) | 0.90 (-1.12, 2.88) | 0.95 (-1.31, 3.10) | 0.98 (-1.38, 3.32) |
| Lark Bunting | Intercept | 135.77 (134.59, 136.90) | 120.68 (118.07, 123.32) | 157.68 (143.83, 171.24) | 118.10 (116.53, 119.66) | 154.10 (144.25, 164.19) | 171.08 (166.43, 176.39) | 179.00 (178.16, 179.84) |
| Lark Bunting | Year | 3.94 (2.43, 5.30) | 3.76 (2.30, 5.11) | 2.44 (0.71, 4.05) | 3.20 (1.22, 5.26) | 11.99 (9.68, 14.27) | 11.37 (9.29, 13.41) | 7.36 (5.15, 9.71) |
| Lark Bunting | Latitude | 3.92 (2.59, 5.26) | 2.66 (1.32, 4.03) | 2.50 (0.86, 4.08) | 3.63 (1.52, 5.70) | 2.04 (0.02, 4.02) | 3.96 (1.49, 6.35) | 4.74 (2.03, 7.57) |
| Lark Bunting | Longitude | -0.81 (-4.61, 2.77) | -2.05 (-6.49, 2.11) | -8.57 (-13.60, -3.77) | -14.01 (-19.98, -8.22) | -10.55 (-17.37, -4.23) | -8.35 (-14.53, -2.83) | -18.69 (-25.07, -11.30) |
| Lark Bunting | Mid green-up | 2.89 (1.02, 4.74) | 3.59 (1.64, 5.47) | 3.83 (1.63, 6.12) | 0.88 (-1.99, 3.75) | 4.02 (0.57, 7.43) | 3.35 (-0.98, 7.69) | 6.00 (1.33, 10.70) |
| Lark Bunting | Precipitation | -0.88 (-1.61, -0.10) | -0.74 (-1.44, -0.07) | -1.89 (-2.78, -1.03) | -3.17 (-4.16, -2.17) | -4.64 (-5.74, -3.49) | -4.79 (-5.80, -3.72) | -4.52 (-5.24, -3.78) |
| Lark Bunting | Temperature | -3.61 (-4.53, -2.73) | -3.89 (-4.96, -2.85) | -3.12 (-4.30, -1.96) | -0.14 (-1.37, 1.06) | 1.27 (-0.05, 2.64) | 3.93 (2.52, 5.36) | 8.39 (6.82, 9.92) |
| Vesper Sparrow | Intercept | 136.05 (135.93, 136.18) | 149.82 (143.66, 155.81) | 148.18 (144.25, 152.22) | 173.63 (171.04, 176.46) | 129.59 (126.78, 132.39) | 167.21 (162.59, 171.87) | 213.64 (203.02, 223.77) |
| Vesper Sparrow | Year | 0.54 (-0.10, 1.28) | 0.88 (-0.02, 1.77) | -0.16 (-1.14, 0.84) | 0.05 (-1.05, 1.11) | 1.83 (0.36, 3.33) | -1.86 (-3.51, -0.21) | -4.04 (-5.46, -2.57) |
| Vesper Sparrow | Latitude | 3.92 (3.52, 4.30) | 3.27 (2.69, 3.82) | 1.52 (0.67, 2.36) | 0.62 (-0.70, 1.93) | 2.42 (1.19, 3.59) | 0.85 (-0.08, 1.86) | -0.29 (-1.43, 0.84) |
| Vesper Sparrow | Longitude | -0.30 (-0.70, 0.10) | -0.36 (-0.91, 0.19) | -0.59 (-1.43, 0.24) | 0.82 (-0.32, 1.92) | 3.16 (2.10, 4.25) | 2.00 (0.97, 3.07) | 0.88 (-0.17, 1.92) |
| Vesper Sparrow | Mid green-up | -3.29 (-3.87, -2.63) | -2.72 (-3.43, -2.03) | -3.16 (-3.92, -2.36) | -1.69 (-2.65, -0.73) | -1.20 (-2.25, -0.19) | -0.76 (-1.70, 0.20) | -0.40 (-1.20, 0.44) |
| Vesper Sparrow | Precipitation | -2.14 (-2.60, -1.68) | -1.91 (-2.36, -1.47) | -1.60 (-2.32, -0.91) | -1.73 (-2.48, -0.99) | -1.40 (-2.28, -0.52) | -3.65 (-4.72, -2.58) | -5.07 (-5.88, -4.26) |
| Vesper Sparrow | Temperature | -2.38 (-2.76, -1.99) | -2.12 (-2.53, -1.73) | -2.61 (-3.08, -2.15) | -3.73 (-4.46, -2.99) | -3.23 (-4.04, -2.43) | -3.17 (-3.83, -2.48) | -2.59 (-3.40, -1.83) |
| LeConte's Sparrow | Intercept | 149.38 (149.04, 149.72) | 157.33 (154.41, 160.25) | 122.37 (120.95, 123.86) | 152.73 (150.89, 154.62) | 194.97 (193.54, 196.32) | 134.94 (134.61, 135.27) | 193.56 (191.71, 195.40) |
| LeConte's Sparrow | Year | -1.64 (-2.50, -0.81) | -0.81 (-1.68, -0.03) | -0.57 (-1.25, 0.06) | 0.10 (-0.56, 0.75) | 0.65 (-0.23, 1.53) | 0.12 (-0.77, 1.02) | 0.62 (-0.31, 1.56) |
| LeConte's Sparrow | Latitude | 5.95 (4.86, 7.07) | 4.95 (4.00, 5.97) | 3.96 (3.12, 4.80) | 3.36 (2.41, 4.29) | 1.88 (0.67, 3.13) | 0.89 (-0.49, 2.15) | -1.36 (-3.16, 0.37) |
| LeConte's Sparrow | Longitude | 1.02 (0.34, 1.68) | 0.93 (0.19, 1.71) | 0.93 (0.04, 1.87) | 1.14 (0.08, 2.23) | -0.02 (-1.06, 1.03) | 0.09 (-0.86, 1.04) | -0.95 (-2.07, 0.19) |
| LeConte's Sparrow | Mid green-up | 0.96 (0.38, 1.62) | 1.16 (0.54, 1.88) | 0.95 (0.32, 1.61) | 0.84 (0.11, 1.56) | 0.07 (-1.00, 1.01) | -0.16 (-1.34, 0.89) | -0.01 (-1.28, 1.19) |
| LeConte's Sparrow | Precipitation | -0.48 (-0.79, -0.17) | -0.48 (-0.79, -0.18) | -0.54 (-0.86, -0.22) | -1.02 (-1.51, -0.56) | -0.90 (-1.55, -0.29) | -0.84 (-1.50, -0.21) | -0.68 (-1.32, -0.07) |
| LeConte's Sparrow | Temperature | -1.65 (-2.00, -1.33) | -1.55 (-1.90, -1.18) | -1.43 (-1.87, -0.99) | -1.51 (-2.05, -0.95) | -1.33 (-1.89, -0.76) | -1.18 (-1.73, -0.63) | -0.96 (-1.58, -0.32) |
| Savannah Sparrow | Intercept | 162.24 (162.08, 162.40) | 139.17 (138.51, 139.77) | 131.44 (127.45, 135.12) | 156.91 (155.46, 158.41) | 204.73 (200.54, 209.26) | 148.72 (139.54, 158.34) | 161.00 (156.90, 165.16) |
| Savannah Sparrow | Year | 0.49 (0.10, 0.87) | 0.18 (-0.25, 0.58) | -0.59 (-1.13, -0.06) | -2.33 (-2.92, -1.74) | -4.03 (-4.68, -3.36) | -4.69 (-5.31, -4.08) | -5.98 (-6.54, -5.40) |
| Savannah Sparrow | Latitude | 4.11 (3.60, 4.66) | 3.54 (2.84, 4.25) | 4.77 (3.70, 5.83) | 2.79 (1.51, 4.04) | 3.42 (2.21, 4.60) | 4.21 (3.12, 5.26) | 4.27 (3.30, 5.16) |
| Savannah Sparrow | Longitude | 0.17 (0.00, 0.36) | 0.10 (-0.13, 0.34) | 1.10 (0.73, 1.47) | 1.31 (0.93, 1.70) | 1.20 (0.79, 1.59) | 1.60 (1.24, 1.95) | 1.61 (1.32, 1.91) |
| Savannah Sparrow | Mid green-up | 0.97 (0.63, 1.29) | 1.37 (0.95, 1.78) | 0.83 (0.26, 1.40) | 0.16 (-0.50, 0.80) | -0.33 (-1.04, 0.38) | -2.24 (-2.89, -1.54) | -2.49 (-3.09, -1.87) |
| Savannah Sparrow | Precipitation | -0.40 (-0.63, -0.18) | -0.60 (-0.87, -0.32) | -0.80 (-1.18, -0.40) | -0.35 (-0.73, 0.03) | -0.79 (-1.19, -0.36) | -0.80 (-1.18, -0.44) | -1.03 (-1.37, -0.70) |
| Savannah Sparrow | Temperature | -3.16 (-3.38, -2.92) | -3.06 (-3.34, -2.78) | -2.69 (-3.12, -2.27) | -2.32 (-2.83, -1.80) | -1.86 (-2.32, -1.38) | -1.69 (-2.08, -1.30) | -2.05 (-2.50, -1.60) |
| Baird's Sparrow | Intercept | 110.56 (110.40, 110.73) | 129.24 (128.46, 130.03) | 152.45 (151.53, 153.44) | 167.36 (164.78, 169.70) | 146.10 (145.45, 146.77) | 151.44 (150.41, 152.41) | 180.44 (179.33, 181.49) |
| Baird's Sparrow | Year | 2.13 (1.17, 3.06) | 2.10 (1.27, 2.93) | 1.09 (-0.01, 2.17) | -0.30 (-1.99, 1.36) | -3.44 (-5.15, -1.73) | -2.50 (-4.19, -0.83) | -0.64 (-2.77, 1.35) |
| Baird's Sparrow | Latitude | 6.69 (4.04, 9.45) | 6.28 (3.32, 9.19) | 4.19 (0.80, 7.54) | 0.46 (-3.59, 4.52) | -1.96 (-7.05, 3.26) | 1.35 (-3.50, 5.96) | 3.71 (-2.19, 9.22) |
| Baird's Sparrow | Longitude | 7.25 (5.35, 9.26) | 5.70 (3.81, 7.65) | 4.00 (1.16, 6.80) | 0.80 (-2.76, 4.06) | -0.50 (-3.92, 2.79) | 3.28 (0.29, 6.36) | 3.62 (0.48, 6.61) |
| Baird's Sparrow | Mid green-up | -2.28 (-3.86, -0.85) | -1.00 (-2.72, 0.77) | 0.83 (-1.08, 2.76) | 2.58 (0.34, 4.87) | 7.77 (4.75, 10.60) | 5.60 (3.22, 8.20) | 2.85 (-0.46, 6.37) |
| Baird's Sparrow | Precipitation | 0.89 (0.12, 1.64) | 0.42 (-0.26, 1.12) | 0.92 (-0.08, 1.89) | 1.87 (0.43, 3.32) | 3.70 (1.99, 5.31) | 4.41 (2.84, 6.05) | 2.40 (0.44, 4.42) |
| Baird's Sparrow | Temperature | -1.49 (-2.31, -0.65) | -1.57 (-2.25, -0.88) | -1.44 (-2.33, -0.56) | -1.35 (-2.54, -0.14) | 1.74 (0.57, 2.87) | 1.94 (0.73, 3.18) | -0.04 (-1.03, 0.99) |
| Henslow's Sparrow | Intercept | 161.86 (161.68, 162.04) | 113.95 (111.74, 115.78) | 135.35 (128.72, 141.58) | 146.03 (142.46, 149.52) | 194.27 (193.28, 195.26) | 140.37 (138.62, 142.05) | 169.12 (165.21, 173.14) |
| Henslow's Sparrow | Year | 0.41 (-1.20, 1.80) | -0.65 (-2.39, 1.08) | -1.48 (-4.00, 0.94) | -4.05 (-7.04, -1.30) | -13.25 (-16.74, -10.00) | -18.13 (-21.54, -14.35) | -13.02 (-18.43, -8.18) |
| Henslow's Sparrow | Latitude | 2.32 (0.36, 4.34) | 2.22 (0.10, 4.30) | 0.71 (-2.20, 3.63) | -1.72 (-5.94, 2.41) | -5.04 (-10.07, -0.20) | -10.10 (-15.71, -4.71) | -19.79 (-27.73, -12.13) |
| Henslow's Sparrow | Longitude | 0.04 (-1.18, 1.26) | 0.55 (-0.68, 1.68) | -0.63 (-2.36, 1.18) | -1.52 (-3.88, 0.84) | -4.53 (-6.96, -2.07) | -1.07 (-4.50, 2.44) | -1.66 (-6.25, 4.26) |
| Henslow's Sparrow | Mid green-up | 1.96 (0.76, 3.10) | 2.63 (1.45, 3.73) | 4.15 (2.40, 5.99) | 7.79 (4.90, 10.77) | 11.55 (8.58, 14.87) | 15.43 (11.92, 18.97) | 19.75 (15.01, 24.77) |
| Henslow's Sparrow | Precipitation | -0.20 (-0.71, 0.35) | -0.27 (-0.81, 0.22) | -1.48 (-2.27, -0.72) | -3.77 (-4.57, -2.91) | -3.51 (-4.55, -2.52) | -1.12 (-1.80, -0.47) | -1.17 (-2.09, -0.13) |
| Henslow's Sparrow | Temperature | -1.19 (-2.79, 0.19) | -1.28 (-2.55, -0.03) | -1.07 (-3.00, 0.88) | -3.02 (-5.58, -0.46) | -0.99 (-4.11, 2.12) | 1.31 (-1.54, 4.49) | 2.10 (-0.65, 4.91) |
| Bobolink | Intercept | 139.21 (138.93, 139.52) | 172.56 (171.87, 173.30) | 118.14 (115.56, 121.04) | 135.48 (133.50, 137.55) | 161.97 (154.79, 168.97) | 178.83 (169.08, 189.33) | 138.60 (134.14, 143.19) |
| Bobolink | Year | 0.33 (0.04, 0.65) | -0.06 (-0.37, 0.27) | -0.51 (-0.93, -0.09) | -0.71 (-1.31, -0.12) | 0.10 (-0.72, 0.95) | 0.77 (0.11, 1.44) | 0.31 (-0.23, 0.90) |
| Bobolink | Latitude | 7.27 (6.66, 7.91) | 7.89 (7.20, 8.54) | 8.16 (7.36, 8.96) | 9.76 (8.97, 10.53) | 11.98 (11.13, 12.82) | 11.58 (10.65, 12.52) | 9.46 (8.45, 10.45) |
| Bobolink | Longitude | 0.41 (0.20, 0.62) | 0.41 (0.19, 0.62) | -0.16 (-0.44, 0.13) | -0.27 (-0.62, 0.09) | 1.21 (0.78, 1.64) | 1.29 (0.87, 1.73) | 0.98 (0.68, 1.28) |
| Bobolink | Mid green-up | 1.15 (0.87, 1.43) | 0.81 (0.46, 1.16) | 0.28 (-0.14, 0.72) | -0.77 (-1.30, -0.26) | -1.40 (-2.04, -0.75) | -2.18 (-2.89, -1.49) | -1.93 (-2.58, -1.29) |
| Bobolink | Precipitation | -0.06 (-0.27, 0.15) | -0.04 (-0.29, 0.20) | -0.21 (-0.46, 0.04) | -0.44 (-0.73, -0.14) | -0.14 (-0.50, 0.23) | 0.17 (-0.22, 0.57) | 0.34 (0.00, 0.65) |
| Bobolink | Temperature | -1.34 (-1.79, -0.83) | -1.00 (-1.50, -0.50) | -1.42 (-1.95, -0.88) | -1.60 (-2.24, -0.94) | -1.01 (-1.77, -0.26) | -1.88 (-2.41, -1.30) | -1.92 (-2.50, -1.37) |
| Western Meadowlark | Intercept | 174.50 (174.34, 174.65) | 113.47 (110.85, 116.13) | 132.71 (121.85, 143.99) | 175.51 (172.21, 179.31) | 138.11 (129.11, 147.11) | 153.29 (151.26, 155.19) | 167.37 (166.46, 168.30) |
| Western Meadowlark | Year | 1.72 (0.89, 2.50) | 2.13 (1.47, 2.78) | 1.98 (1.32, 2.66) | 3.05 (2.22, 3.87) | 2.52 (1.76, 3.26) | 0.36 (-0.27, 1.07) | 1.34 (0.47, 2.22) |
| Western Meadowlark | Latitude | 4.35 (2.80, 5.77) | 2.42 (0.96, 4.06) | 3.09 (1.59, 4.64) | 1.85 (-0.22, 3.86) | -0.69 (-2.44, 1.23) | -1.68 (-3.19, -0.20) | -1.57 (-3.31, -0.10) |
| Western Meadowlark | Longitude | 3.95 (2.63, 5.25) | 3.82 (2.30, 5.46) | 6.09 (4.63, 7.52) | 4.49 (2.50, 6.44) | 1.80 (-0.05, 3.67) | 0.62 (-1.13, 2.31) | 0.99 (-1.25, 3.07) |
| Western Meadowlark | Mid green-up | -0.70 (-1.57, 0.16) | -2.11 (-3.25, -0.95) | -2.86 (-4.01, -1.74) | -1.75 (-3.17, -0.27) | -1.05 (-2.34, 0.25) | 0.15 (-1.18, 1.47) | 0.45 (-0.87, 1.73) |
| Western Meadowlark | Precipitation | 0.67 (0.07, 1.26) | 0.57 (-0.09, 1.21) | -0.07 (-0.79, 0.69) | 0.25 (-0.59, 1.09) | -0.07 (-0.87, 0.76) | 0.36 (-0.39, 1.12) | -0.13 (-0.83, 0.60) |
| Western Meadowlark | Temperature | -1.93 (-2.61, -1.22) | -1.26 (-1.94, -0.58) | -0.46 (-1.14, 0.17) | -0.40 (-1.33, 0.53) | 0.73 (0.00, 1.47) | 0.50 (-0.31, 1.23) | -0.89 (-1.83, 0.03) |
| Eastern Meadowlark | Intercept | 165.34 (165.04, 165.63) | 147.13 (142.55, 151.59) | 139.29 (136.38, 142.14) | 169.47 (166.51, 172.33) | 126.09 (123.51, 128.62) | 154.85 (150.53, 159.81) | 201.67 (192.09, 211.74) |
| Eastern Meadowlark | Year | 1.27 (0.61, 1.97) | 1.99 (1.15, 2.83) | 3.44 (2.36, 4.46) | 0.87 (-0.14, 1.88) | -0.49 (-1.60, 0.62) | -1.88 (-2.88, -0.81) | -1.67 (-2.79, -0.56) |
| Eastern Meadowlark | Latitude | 0.22 (-0.76, 1.16) | -0.22 (-1.29, 0.89) | 0.57 (-0.73, 1.86) | 5.97 (4.62, 7.33) | 5.16 (3.77, 6.58) | 1.24 (-0.11, 2.58) | 1.28 (-0.10, 2.72) |
| Eastern Meadowlark | Longitude | -3.36 (-4.04, -2.69) | -3.31 (-4.16, -2.48) | -5.08 (-6.22, -3.96) | -5.51 (-6.64, -4.30) | -5.56 (-6.97, -4.14) | -2.70 (-3.89, -1.42) | -1.15 (-2.57, 0.10) |
| Eastern Meadowlark | Mid green-up | 3.09 (2.64, 3.53) | 2.94 (2.31, 3.60) | 3.29 (2.58, 3.99) | 1.12 (0.40, 1.85) | 0.50 (-0.20, 1.18) | 0.38 (-0.30, 1.07) | -0.13 (-0.89, 0.56) |
| Eastern Meadowlark | Precipitation | -0.05 (-0.31, 0.20) | 0.25 (-0.10, 0.59) | -0.19 (-0.54, 0.14) | -0.46 (-0.86, -0.04) | -0.81 (-1.27, -0.32) | -0.96 (-1.47, -0.41) | -0.42 (-1.04, 0.08) |
| Eastern Meadowlark | Temperature | -0.11 (-0.77, 0.53) | -0.55 (-1.36, 0.32) | 0.09 (-0.94, 1.14) | -0.02 (-1.03, 1.01) | -1.38 (-2.54, -0.21) | -2.67 (-3.82, -1.56) | -2.27 (-3.35, -1.26) |
| Dickcissel | Intercept | 167.72 (167.57, 167.87) | 142.29 (141.73, 142.84) | 151.89 (145.93, 157.58) | 162.85 (161.56, 164.08) | 124.27 (122.63, 125.76) | 146.66 (136.49, 156.57) | 183.21 (177.46, 188.59) |
| Dickcissel | Year | -3.73 (-4.09, -3.39) | -3.70 (-4.11, -3.31) | -3.21 (-3.68, -2.74) | -2.82 (-3.34, -2.32) | -3.10 (-3.62, -2.58) | -1.52 (-2.15, -0.93) | -0.62 (-1.08, -0.14) |
| Dickcissel | Latitude | 11.72 (11.16, 12.26) | 12.23 (11.70, 12.78) | 14.15 (13.48, 14.81) | 13.02 (12.23, 13.78) | 12.58 (11.74, 13.43) | 9.77 (8.85, 10.68) | 9.26 (8.44, 10.05) |
| Dickcissel | Longitude | -3.79 (-4.34, -3.20) | -3.31 (-3.83, -2.80) | -3.66 (-4.34, -2.96) | -2.61 (-3.44, -1.84) | -3.76 (-4.61, -2.92) | -3.70 (-4.57, -2.80) | -4.79 (-5.60, -4.01) |
| Dickcissel | Mid green-up | 0.62 (0.31, 0.95) | 0.88 (0.61, 1.14) | 0.07 (-0.25, 0.39) | -0.51 (-0.85, -0.17) | -1.79 (-2.18, -1.39) | -1.73 (-2.16, -1.31) | -1.97 (-2.34, -1.57) |
| Dickcissel | Precipitation | 0.50 (0.31, 0.69) | 0.17 (-0.01, 0.36) | 0.19 (-0.01, 0.40) | 0.17 (-0.09, 0.43) | 0.23 (-0.05, 0.52) | 0.15 (-0.08, 0.39) | 0.17 (-0.07, 0.38) |
| Dickcissel | Temperature | -0.01 (-0.49, 0.42) | -0.28 (-0.77, 0.19) | -1.44 (-1.99, -0.87) | -1.49 (-2.05, -0.88) | -3.24 (-3.79, -2.66) | -3.00 (-3.75, -2.28) | -3.12 (-3.73, -2.53) |

Supplemental references

Anteau, M. J., Ellis, K. S., MacDonald, G. J., Igl, L. D., Niemuth, N. D., & Vest, J. L. (2023). Climate-induced shifts in grassland bird nesting phenology have implications for grassland management. *Global Ecology and Conservation*, *48*, e02700.

Austin, J. E., Shaffer, T. L., Igl, L. D., Johnson, D. H., Krapu, G. L., Larson, D. L., Mech, L. D., Mushet, D. M., & Sovada, M. A. (2017). Northern Prairie Wildlife Research Center—celebrating 50 years of science: U.S. Geological Survey Circular 1434, 27 p., <https://doi.org/10.3133/cir1434>.

Boyle, W. A. (2023). CBS02 Nests of Grasshopper Sparrows on Konza Prairie ver 4. Environmental Data Initiative. <https://doi.org/10.6073/pasta/0f4cb15d8075d6d7a735cfe57a9603b8>.

Buhl, T. K., & Koford, R. R. (2023). Density and Fledging Success Data from Conservation Reserve Program Fields in North Dakota and Minnesota, 1991-1993: U.S. Geological Survey data release, <https://doi.org/10.5066/P9DRTDGU>.

Burr, P. C., Robinson, A. C., Larsen, R. T., Newman, R. A., & Ellis-Felege, S. N. (2017). Sharp-tailed grouse nest survival and nest predator habitat use in North Dakota’s Bakken oil field. *PLoS One*, *12*(1), e0170177.

Carpenter, B., Gelman, A., Hoffman, M. D., Lee, D., Goodrich, B., Betancourt, M., Brubaker, M., Guo, J., Li, P., & Riddell, A. (2017). Stan: A probabilistic programming language. *Journal of Statistical Software*, *76*, 1–32.

Chesser, R. T., Billerman, S. M., Burns, K. J., Cicero, C., Dunn, J. L., Hernández-Baños, B. E., Jiménez, R. A., Johnson, O., Mason, N. A., & Rasmussen, P. C. (2025). Sixty-sixth supplement to the American Ornithological Society’s Check-list of North American Birds. *Ornithology*, *142*(3), ukaf015.

DeVries, J. H., Rimer, S. O., & Walsh, E. M. (2010). Cropland nesting by Long-billed Curlews in southern Alberta. *The Prairie Naturalist*, *43*(3/4), 123–129.

Fidino, M. (2022). mfidino/bates_phenology: JAE revision (v1.0). Zenodo. <https://doi.org/10.5281/zenodo.6025222>.

Jensen, W., & Sandercock, B. K. (2020). PBG05 Response of bird abundance to the Patch-Burn Grazing experiment at Konza Prairie ver 3. Environmental Data Initiative. <https://doi.org/10.6073/pasta/00a8f9dc10f0086586fe3ac3bf6ef185>.

Klett, A. T., Shaffer, T. L., & Johnson, D. H. (1988). Duck nest success in the Prairie Pothole Region. *The Journal of Wildlife Management*, 431–440.

Ribic, C., & Herkert, J. (2023). Nest initiation and end dates for eight grassland bird species in Wisconsin and Illinois [Dataset]. Dryad. <https://doi.org/10.5061/dryad.cjsxksnb8>.

Rumble, M. A., Herman-Brunson, K. M., Jensen, K. C., Kaczor, N. W., Swanson, C. C., & Klaver, R. W. (2011). Data product containing nest site selection and survival data for "Nesting ecology of greater sage-grouse Centrocercus urophasianus at the eastern edge of their historic distribution". Fort Collins, CO: U.S. Department of Agriculture, Forest Service, Rocky Mountain Research Station. <https://doi.org/10.2737/RDS-2011-0004>.

Sinnott, E. A., Thompson, F. R., Hedges, K. R., Loncarich, F. L., Thompson, T. R., & Mosloff, A. R. (2023). Northern bobwhite adult breeding season and nest survival Missouri 2014-2018 [Dataset]. Dryad. <https://doi.org/10.5061/dryad.kkwh70s9f>.

Weigand, J. P. (1980). Ecology of the Hungarian Partridge in North-Central Montana. *Wildlife Monographs*(74), 3–106.

Yu, K., & Moyeed, R. A. (2001). Bayesian quantile regression. *Statistics & Probability Letters*, *54*(4), 437–447.
